# *Zymomonas* diversity and potential for biofuel production

**DOI:** 10.1186/s13068-021-01958-2

**Published:** 2021-05-01

**Authors:** Magdalena M. Felczak, Robert M. Bowers, Tanja Woyke, Michaela A. TerAvest

**Affiliations:** 1 Department of Biochemistry and Molecular Biology, Michigan State University, East Lansing, MI 48824 USA; 2 U.S. Department of Energy Joint Genome Institute, Lawrence Berkeley National Laboratory, Berkeley, CA 94720 USA

## Abstract

**Background:**

*Zymomonas mobilis* is an aerotolerant α-proteobacterium, which has been genetically engineered for industrial purposes for decades. However, a comprehensive comparison of existing strains on the genomic level in conjunction with phenotype analysis has yet to be carried out. We here performed whole-genome comparison of 17 strains including nine that were sequenced in this study. We then compared 15 available *Zymomonas* strains for their natural abilities to perform under conditions relevant to biofuel synthesis. We tested their growth in anaerobic rich media, as well as growth, ethanol production and xylose utilization in lignocellulosic hydrolysate. We additionally compared their tolerance to isobutanol, flocculation characteristics, and ability to uptake foreign DNA by electroporation and conjugation.

**Results:**

Using clustering based on 99% average nucleotide identity (ANI), we classified 12 strains into four clusters based on sequence similarity, while five strains did not cluster with any other strain. Strains belonging to the same 99% ANI cluster showed similar performance while significant variation was observed between the clusters. Overall, conjugation and electroporation efficiencies were poor across all strains, which was consistent with our finding of coding potential for several DNA defense mechanisms, such as CRISPR and restriction–modification systems, across all genomes. We found that strain ATCC31821 (ZM4) had a more diverse plasmid profile than other strains, possibly leading to the unique phenotypes observed for this strain. ZM4 also showed the highest growth of any strain in both laboratory media and lignocellulosic hydrolysate and was among the top 3 strains for isobutanol tolerance and electroporation and conjugation efficiency.

**Conclusions:**

Our findings suggest that strain ZM4 has a unique combination of genetic and phenotypic traits that are beneficial for biofuel production and propose investing future efforts in further engineering of ZM4 for industrial purposes rather than exploring new *Zymomonas* isolates.

**Supplementary Information:**

The online version contains supplementary material available at 10.1186/s13068-021-01958-2.

## Background

*Zymomonas mobilis* is becoming a popular bacterial host for biofuel research due to its fast growth, tolerance of ethanol and toxins found in lignocellulosic hydrolysates (lignotoxins), and high specificity of ethanol production [1]. However, there are also key challenges for working with *Z. mobilis* that differ significantly from working with other biofuel producers, such as yeast. Specifically, its use of the Entner–Doudoroff glycolytic pathway is poorly understood, it lacks a well-developed genetic toolbox, and it does not have a long history of industrial use [1]. As a result of these challenges, it has been difficult to convert *Z. mobilis* from an efficient ethanologen into an advanced biofuel platform. Moving forward, biofuel production efforts must focus on advanced fuels and improve microbe capabilities to use the wide range of substrates available in lignocellulosic hydrolysates. Significant investment is thus necessary to build the tools and knowledge required to enable rapid and effective engineering of *Z. mobilis* for these features.

One approach to improve biofuel-producing organisms is to take advantage of naturally occurring genetic diversity within a species or genus, as has been successful in yeast [2]. Comparative genomics is now starting to be utilized in *Zymomonas* and several *Z. mobilis* genomes were compared in a recent analysis [3]. This comparison showed a high degree of similarity between *Zymomonas* genomes, although different strains had different plasmid sets. This is in line with an earlier phenotypic characterization which showed relatively low diversity, and only one species (*mobilis*) within the genus *Zymomonas* [4]. However, the previous studies did not combine genome sequence analysis with phenotypic analysis, therefore, it is difficult to make inferences about gene function from their datasets. We hypothesized that by expanding the set of available genomes in conjunction with performing physiological characterization of all accessible strains, new connections between specific genes, operons, and/or plasmids and phenotypic biofuel-relevant traits could be revealed.

To better understand *Z. mobilis* as a platform organism, we combined a comparative genomics approach with physiological characterization. Specifically, we compared the genomes of 17 strains, including 8 which had previously been sequenced [3, 5–11] and 9 which were sequenced at the Joint Genome Institute for this study (Table 1). Specific sequencing information, such as read coverage for each genome sequenced in this study is in Additional file 1: Table S1. Here, strains are defined as separate isolates or derivates that have been deposited to strain collections under unique identifiers. The strains were originally isolated from several locations around the world, across North and South America, Africa, and Europe (Additional file 1: Table S2). The strains were primarily obtained from fermentations intended to produce alcohol for human consumption. One strain (PROIMI A1) was isolated specifically for research purposes [12]. In some cases, the *Zymomonas* was a normal component of the fermentation and in other cases (European isolates) it was considered a contaminant [13]. While *Zymomonas* strains have previously been found in environmental samples, including bees, we were not able to obtain any such isolates [14]. Future studies may be able to expand knowledge of the *Zymomonas* genus by focusing on strains not isolated from human-associated fermentations. We compared the strains for their growth rates in rich media, tolerance of lignocellulosic hydrolysate and isobutanol, conjugation and transformation efficiencies, and flocculation characteristics.

**Table 1:** Information on Zymomonas genomes

Common	IMG.taxID	NCBI accession #	SRA accession #	Gene count	Scaffold count	hierBAPS cluster	ANI_99 cluster	Genome reference
ZM4	2834884446	NZ_CP023715		2008	5	1	7	[5]
B-1960	2834983939	NZ_CP021053		1837	1	1	4: CP1	[6]
CP1	2811994965	NZ_VIVG01000001.1	SRP196443	1823	4	1	4: CP1	This study
CP3	2818991419	NZ_VIVT01000001.1	SRP196448	1670	22	1	2: CP4	This study
CP4	2545555880	NC_022900		1933	6	1	2: CP4	[7]
B-12526	2558860209	NZ_CP003709		1916	6	1	2: CP4	[3]
Z6	2634166410	NC_018145		1847	4	4	3: Z6	[8]
B-23394	2811994876	NZ_VFNW01000001.1	SRP196447	1869	4	4	3: Z6	This study
B-4492	2811994895	NZ_VFOD01000001.1	SRP196445	1873	5	4	3: Z6	This study
ATCC 10988	645058785	NZ_ACQU01000001		1894	24	2	1: Drainas	[9]
CU1	2811994958	NZ_VFOH01000001.1	SRP196503	1880	3	2	1: Drainas	This study
CU1rif2	2811994957	NZ_VIVF01000001.1	SRP196507	1896	4	2	1: Drainas	This study
uvs51	2811994963	NZ_VFOI01000001.1	SRP196501	1903	5	2	1: Drainas	This study
PROIMI A1	2811994956	NZ_VFOG01000001.1	SRP196506	1877	4	1	6	This study
NCIMB11163	646311969	NC_013355		1978	4	1	8	[10]
*francensis*	2811994955	NZ_VFOF01000001.1	SRP196505	1882	7	3	5	This study
*pomaceae*	650716107	NC_015709		1838	3	3	9	[11]

Common strain names were used in the table and in the text. Other names used by different strain collections are listed in Additional file 1: Table S2 for cross-reference. For strains with multiple scaffolds, NCBI Acc # for the largest contig is shown; smaller contigs have consecutive numbers. Contigs recognized as plasmids are listed in Additional file 1: Table S3 with specific accession numbers. SRA accession numbers are provided for the genomes sequenced for this study

We obtained and physiologically characterized as many of the *Zymomonas* strains as possible using three publicly available culture collections; the American Type Culture Collection (ATCC), the German Collection of Microorganisms and Cell Cultures (DSMZ), and the USDA Agricultural Research Service Culture Collection (NRRL). Two strains, NCIMB11163 and B-12526, were not readily available from culture collections, resulting in a total of 15 strains for phenotyping.

## Results and discussion

### Phylogenetic analysis

**Fig. 1:**
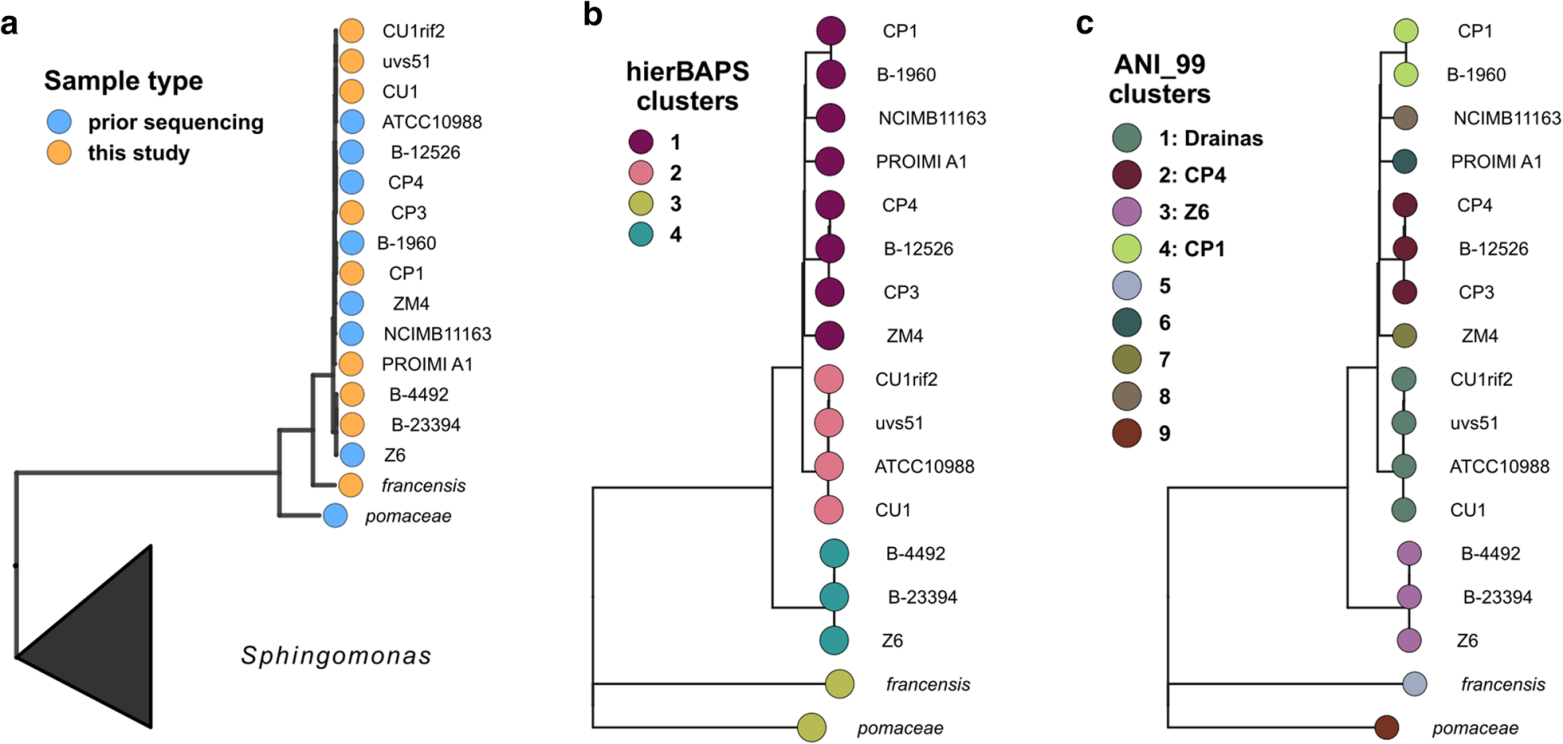
Strain-level resolution across 17 *Zymomonas* genomes. **a** Whole genome trees based on the alignment and concatenation of 56 universal marker genes. **b**, **c** SNP trees of the same 17 *Zymomonas* where genomes are colored by either a hierarchical Bayesian clustering of the whole-genome alignment (**b**) or by the groups identified as a result of 99% ANI clusering (alignment fraction greater than 70%)

We explored the phylogenetic relationships of all 17 *Zymomonas* strains by first constructing a concatenated marker gene tree using a set of 56 universal single copy markers [15]. Based on this tree, most *Zymomonas* isolates were highly similar, with a slight branching of the *pomaceae* and *francensis* subspecies in the marker gene tree (Fig. 1a). To increase resolution, a single nucleotide polymorphism (SNP) tree was constructed, which showed increased branching patterns between the genomes [16]. We analyzed the branching pattern together with two separate clustering approaches, hierarchical Bayesian clustering (hierBAPS) [17, 18] and 99% average nucleotide identity (ANI) clustering (Fig. 1b, c). The hierBAPS approach placed the isolates into 4 distinct genome clusters, while 9 clusters resulted from the 99% ANI clustering. Based on 99% ANI clustering, most of the 17 sequenced *Zymomonas* strains can be classified into four multi-strain clusters, which we refer to as CP1 (ANI_99 cluster 4), CP4 (ANI_99 cluster 2), Z6 (ANI_99 cluster 3), and Drainas (ANI_99 cluster 1) (Table 1; Fig. 1). Each group was named for the most commonly studied strain within the group, except for the Drainas group, which was named for the research group that generated three of the four strains. Five strains did not cluster with any other strains based on 99% ANI, including *Z. mobilis* subsp. *pomaceae*, *Z. mobilis* subsp. *francensis*, PROIMI A1, NCIMB11163, and ZM4. Throughout this work, we use ZM4 as the comparator strain, and group PROIMI A1, *francensis*, *pomaceae* and NCIMB11163 together in a ‘none’ group. In general, the clusters align with our expectations based on the history of each strain as described below.

The CP1 group consists of two strains, CP1 and B-1960. Strain CP1 was isolated from fermenting sugarcane juice in Recife, Brazil [19] and strain B-1960 was isolated from spoiled beer by H.J. Bunker of Barclay Perkins & Co., Ltd (Table 1). The CP4 group includes two strains that were isolated from the same market as CP1 (CP3 and CP4), and a flocculating variant of CP4 developed at Oak Ridge National Lab, B-12526 [20]. Three additional strains fall within this large branch but do not fall within one of the clustering groups, PROIMI A1, NCIMB11163, and ZM4. Strain PROIMI A1 was isolated in Argentina from fermenting sugarcane juice. PROIMI A1 has not been widely studied, but previous work shows that it flocculates readily and has plasmids that can be repurposed for genetic modification purposes [12]. NCIMB11163 was isolated from ale in England in the 1970s [21]. The origin of strain ZM4 is somewhat unclear, because it is either a derivative or contaminant of CP4. After several decades of research on CP4, research groups realized that not all strains designated CP4 were identical and designated ZM4 as a separate group because of differing plasmid profiles [22]. Despite the history of the two strains, there are significant differences in their genome sequences [7]. In our analysis, ZM4 did not cluster with either the CP1 or CP4 groups, despite the expectation that they would be close relatives based on strain history.

The Z6 group is formed by three strains; two that were isolated from fermenting palm sap in Zaire (Z6 and B-23394), and a third, B-4492, which is listed in the NRRL catalog as identical to strain B-1960 (from the CP1 group) [23]. We observed that strain B-4492 is phenotypically and genetically much more similar to the strains originating in Zaire than to the CP1 group. We suggest that strain B-4492 is likely an isolate from Zaire and therefore include it with the Z6 group.

The Drainas group is formed by the type strain, ATCC10988, and three derivative strains created by mutagenesis and selection by Dr. Constantin Drainas and coauthors. ATCC10988 was isolated from fermenting agave sap in Mexico in 1924 [4]. Strain CU1 was developed by incubating ATCC10988 with acridine orange and was reported to have a decreased tolerance to glucose concentrations ≥ 20% [24]. Strain CU1rif2 is a derivative of CU1 developed by further treatment with acridine orange and screening for rifampicin resistance [25]. Strain uvs51 is a derivative of CU1rif2 and was developed by mutagenizing with MNNG and screening for UV sensitivity [26]. Based on the relationships between these strains, we expect all three derivative strains to be sensitive to high glucose concentrations and CU1rif2 and uvs51 to be resitant to rifampicin. All strains showed the expected phenotypes (Additional file 1: Figure S1).

*Z. mobilis* subsp. *pomaceae* and *Z. mobilis* subsp. *francensis* were the most genetically distinct from the rest of the *Zymomonas* strains. *Z. mobilis* subsp. *francensis* strains were isolated from contaminated cider in France in 2001 [27]. These strains were designated as a separate subspecies based on differences in proteome (as observed by SDS-PAGE) and differences from subsp. *mobilis* and subsp. *pomaceae* in sucrose fermentation and tolerance of bile salts. Similarly, *Z. mobilis* subsp. *pomaceae* strains were isolated from contaminated cider in England [13].

### Zymomonas mobilis pangenome

**Fig. 2:**
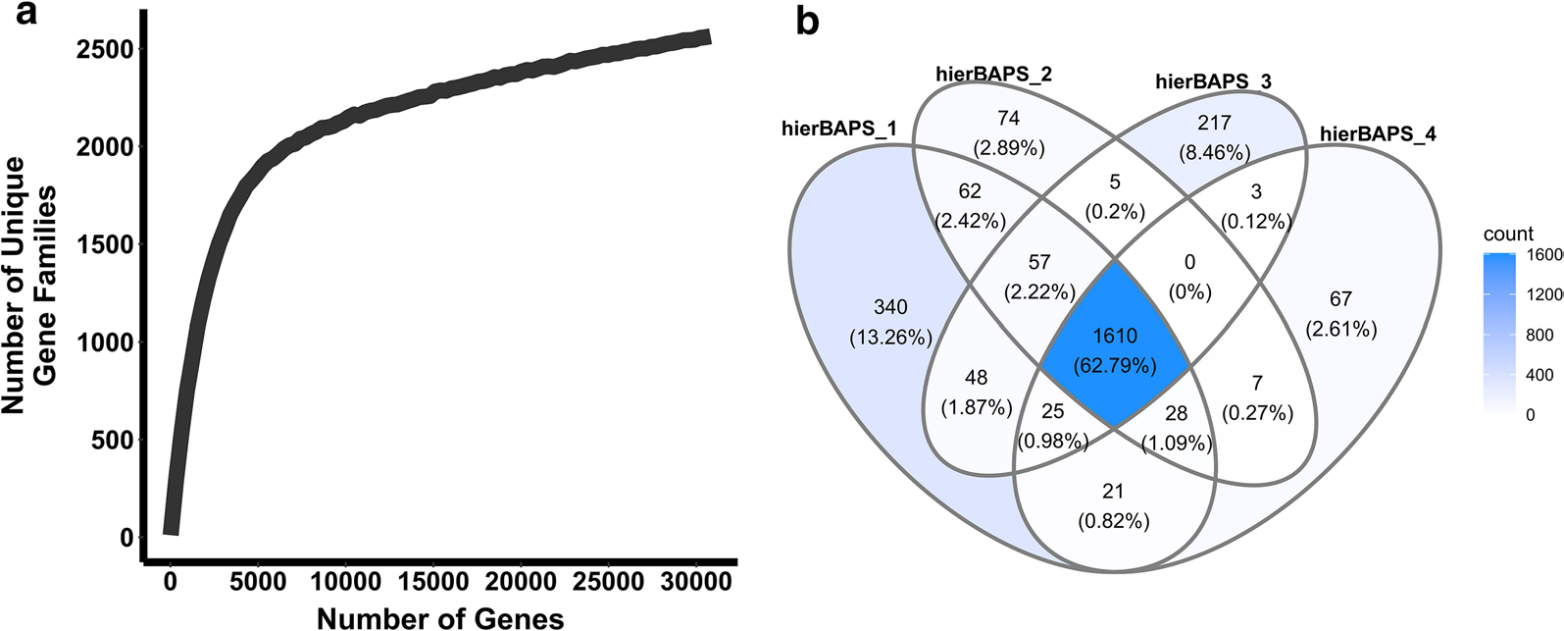
Pangenome of 17 Zymomonas genomes as determined based on the number of unique gene families/orthologues observed in each genome. **a** Rarefaction curve of the number of unique gene families compared to the total sampled. **b** Venn diagram grouped into 4 ellipses where each ellipse corresponds to a dereplicated set of orthologue ids (a.k.a. genes) from genome sets corresponding to the hierBAPs SNP genome clusters

The pangenome of the *Zymomonas* strains was assessed by assigning genes to ortholog clusters and generating a rarefaction curve by plotting the number of unique orthologue groups as a function of total gene content, with a step size of 100 genes (Fig. 2a). A Venn diagram was also generated to visualize the number of ortholog clusters shared or unique among the four hierBAPS clusters (Fig. 2b). There were 2,564 unique gene families and while the rarefaction curve appears to approach an asymptote, the number of unique gene families is still increasing, suggesting that there are more unique gene families to be discovered when increasing the number of sampled genomes, i.e., the pangenome may be open. The Venn diagram does display however, that most unique gene families are observed in the core (62%, dark blue), suggesting that while we do not yet have a closed pangenome, these *Zymomonas* genomes have a relatively small accessory component.

### Zymomonas mobilis plasmids

Beyond chromosome sequences, PacBio sequencing revealed multiple circular plasmids in 6 of 9 genomes analyzed. In two strains (CP1 and CU1), only small linear contigs were found, which may represent incompletely sequenced or linear plasmids. CP3 was assembled in 22 linear contigs, some of which may represent plasmids. The new and previously identified plasmids and linear contigs are summarized in Table 2 and accession numbers for all contigs and topology determination are included in Additional file 1: Table S3. Each group of strains appears to have a common set of plasmids, which share over 99.97% ANI. There is little homology between plasmids from different groups. Interestingly, three out of four plasmids of ZM4 share partial homolgy with plasmids from two other groups: CP4 and Drainas. Namely, 64% of p32.8 has 96% ANI to p32.3 of ATCC10988 and 78 and 61% of p36.5 and p39.3 share 98% ANI with p36.9 and p32.4 of the CP4 group, respectively. It is possible that ZM4 evolved from CP4 but developed a unique set of plasmids through conjugation with other *Zymomonas* strains during serial passage in the laboratory, which would explain its unusual history.

**Table 2:**
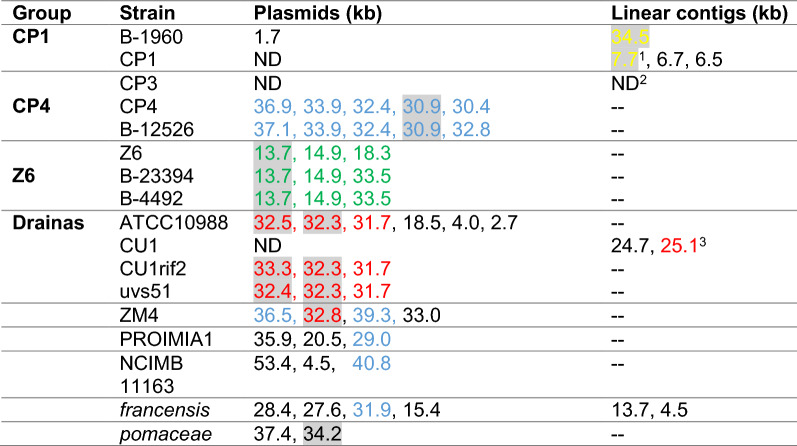
Summary of *Zymomonas mobilis* plasmids and their Type I R–M systems (*hsdRMS* systems)

Plasmids are named by size as determined in NCBI nucleotide database. Full names and NCBI accession numbers of all plasmids are listed in Additional file 1: Table S3. BLASTn of whole plasmids sequences was used to determine their ANI. Plasmids shared by more than one strain in the group were assigned the same colors, i.e., all blue-labeled plasmid were shared by multiple strains in the CP4 group. Homologous plasmids (> 99% ANI) are vertically aligned, i.e., the blue-labeled plasmids in the ZM4 row are homologous to the corresponding blue-labeled plasmids in the CP4 row. Plasmids with no significant homology to other plasmids are shown in black. Plasmids with annotated *hsdRMS* genes are highlighted (see details in text). ND-not detected

^1^*hsdMS* and truncated *hsdR* with 100% aa identity to *hsdRMS* on linear contig 34.5 of B-1960

^2^The CP3 chromosome could not be assembled into a circular contig; the genome could only be assembled into 22 linear contigs. Therefore, we could not distinguish between putative chromosomal and plasmid contigs.

^3^99% nucleotide identity to p32.3 of ATCC10988 but no *hsdRMS* genes

One of the most important features found on extrachromosomal elements is the Type I restriction–modification (R–M) system, encoded by *hsdRMS* genes. At least one such system was found on plasmids in all four groups and in ZM4 and *pomaceae* (highlighted in Table 2). Consistent with the ANI between the plasmids, BLASTp confirmed almost 100% amino acid identity between the R-M subunits (Restriction, Methylation, and Specificity) within the same group. Interestingly, although R and M subunits encoded on p32.8 of ZM4 show 99% amino acid identity to respective proteins encoded on p32.3 of ATCC10988, the putative S subunit is annotated as a hypothetical protein and has only 39% homology to respective subunit of ATCC10988. Similarly, although R and M subunits of the R–M system in *pomaceae* show 95% amino acid identity to subunits on CP4′s p30.9, the S subunit has only 38% identity to the same subunit of CP4. Hence, the specificity factor of Type I R–M system seems to evolve faster than endonuclease and methylase, possibly as a result of adaptation to new foreign DNA sequences present in a changing environment. Except for those in ZM4 and *pomaceae,* no *hsdRMS* genes were found on plasmids present in strains outside of the four groups. It is noteworthy that ZM4 was the only strain with *hsdMS* genes found on the chromosome without a corresponding *hsdR* identified.

### Comparison of growth in rich medium

**Fig. 3:**
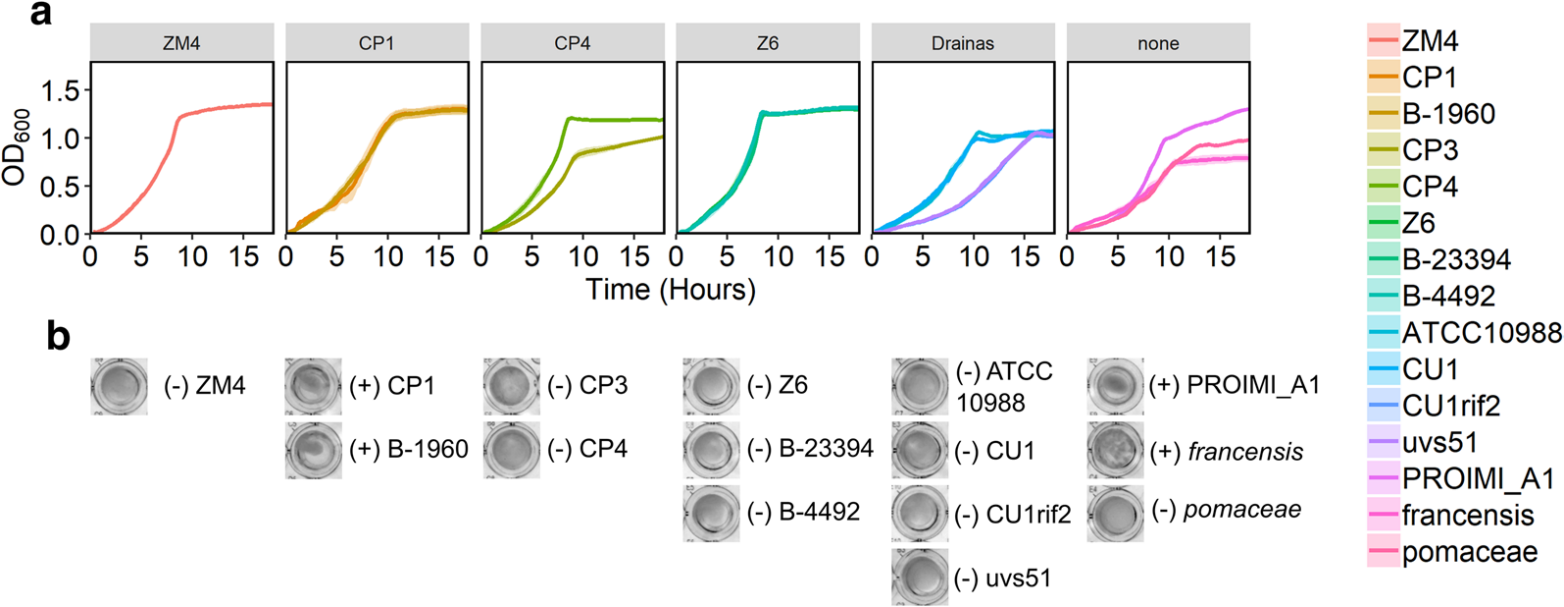
Growth curves of *Zymomonas* strains. Strains were grown in rich medium in 96-well plates with shaking, in anoxic conditions. Represented by **a** OD_600_ versus time and **b** representative images of wells after 45 h of growth showing varying levels of flocculation with a ( +) or (−) indicating whether flocs were present or absent based on visual inspection. In A, lines represent the average of three biological replicates and ribbons represent standard error

We compared growth of all strains in rich medium in anoxic conditions to determine growth rates (Fig. 3). Grouping the growth curves based on the phylogenetic tree reveals similarities within the groups. ZM4 and strains in the Z6 group have the fastest growth rates. Strains in the CP1 and CP4 groups grew more slowly than ZM4 and Z6. The type strain and its derivatives in the Drainas group show that two of the mutants have a growth defect compared with the parent strain. Of the strains that could not be classified, the *francensis* subspecies grew more slowly than all other strains. The *pomaceae* subspecies also grew slowly.

The exponential growth rates and doubling times for the growth curves were calculated by fitting logistic curves to the data in Fig. 3 and are reported in Table 3. Strains in the Z6 group along with ZM4 and CP4 showed the highest growth rates and shortest doubling times. Flocculation of some strains (Fig. 3b) caused instability in the apparent growth rates, making the doubling time an unreliable summary statistic for some of these growth curves. Therefore, we also report the area under the curve after 20 h (AUC_20_) as a summary of growth, which takes into account both the growth rate and the final density [28]. ZM4 and Z6 group strains showed the highest growth as measured by AUC_20_, the CP1, CP4 and Drainas groups showed intermediate growth, and the unclassified strains showed variable growth with *francensis* showing the lowest growth (Table 3).

**Table 3:** Growth statistics for *Zymomonas* strains calculated from data in Fig. 3

Group	Strain	r (h^−1^)	Doubling time (h)	AUC_20_
CP1	B-1960	0.53 ± 0.03	1.30 ± 0.07	16.55 ± 0.93
	CP1	0.58 ± 0.02	1.19 ± 0.04	16.52 ± 1.00
	CP3	0.55 ± 0.02	1.27 ± 0.06	12.00 ± 0.43
CP4	CP4	0.79 ± 0.06	0.88 ± 0.06	16.20 ± 0.39
	Z6	0.74 ± 0.04	0.95 ± 0.06	17.5 ± 0.40
Z6	B-23394	0.71 ± 0.03	0.98 ± 0.04	17.40 ± 0.15
	B-4492	0.79 ± 0.09	0.89 ± 0.09	17.62 ± 0.35
Drainas	ATCC10988	0.64 ± 0.02	1.09 ± 0.02	13.25 ± 0.50
	CU1	0.53 ± 0.02	1.32 ± 0.04	13.2 ± 0.35
	CU1rif2	0.40 ± 0.01	1.75 ± 0.06	9.43 ± 0.13
	uvs51	0.39 ± 0.03	1.78 ± 0.12	9.68 ± 0.12
	ZM4	0.67 ± 0.01	1.03 ± 0.01	17.59 ± 0.25
	PROIMIA1	0.58 ± 0.01	1.20 ± 0.03	14.43 ± 0.23
	*francensis*	0.47 ± 0.02	1.46 ± 0.05	9.97 ± 0.68
	*pomaceae*	0.50 ± 0.02	1.38 ± 0.04	10.80 ± 0.26

Logistic curves were fit to the measured growth curves using the R package ‘growthcurver’. The calculated exponential growth rate (r), doubling time, and area under the curve after 20 h (AUC_20_) are reported as average ± standard error (N = 3)

### Comparison of growth in lignocellulosic hydrolysate

**Fig. 4:**
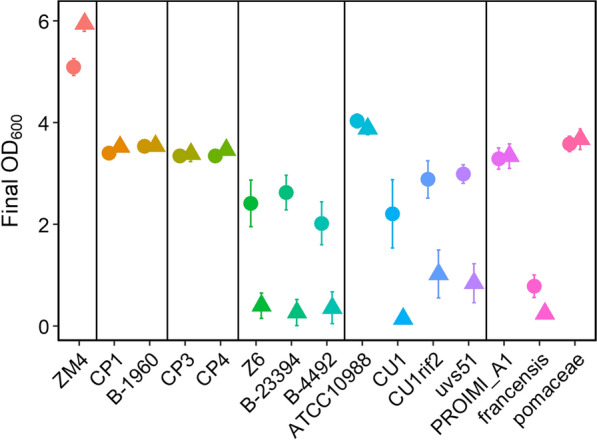
Growth of *Zymomonas* strains in lignocellulosic hydrolysate. The final OD_600_ after 55 h of growth is shown. Cultures were inoculated to a starting OD_600_ of 0.1 (triangles) or 0.5 (circles). Points represent the average of three biological replicates and error bars represent standard error

**Fig. 5:**
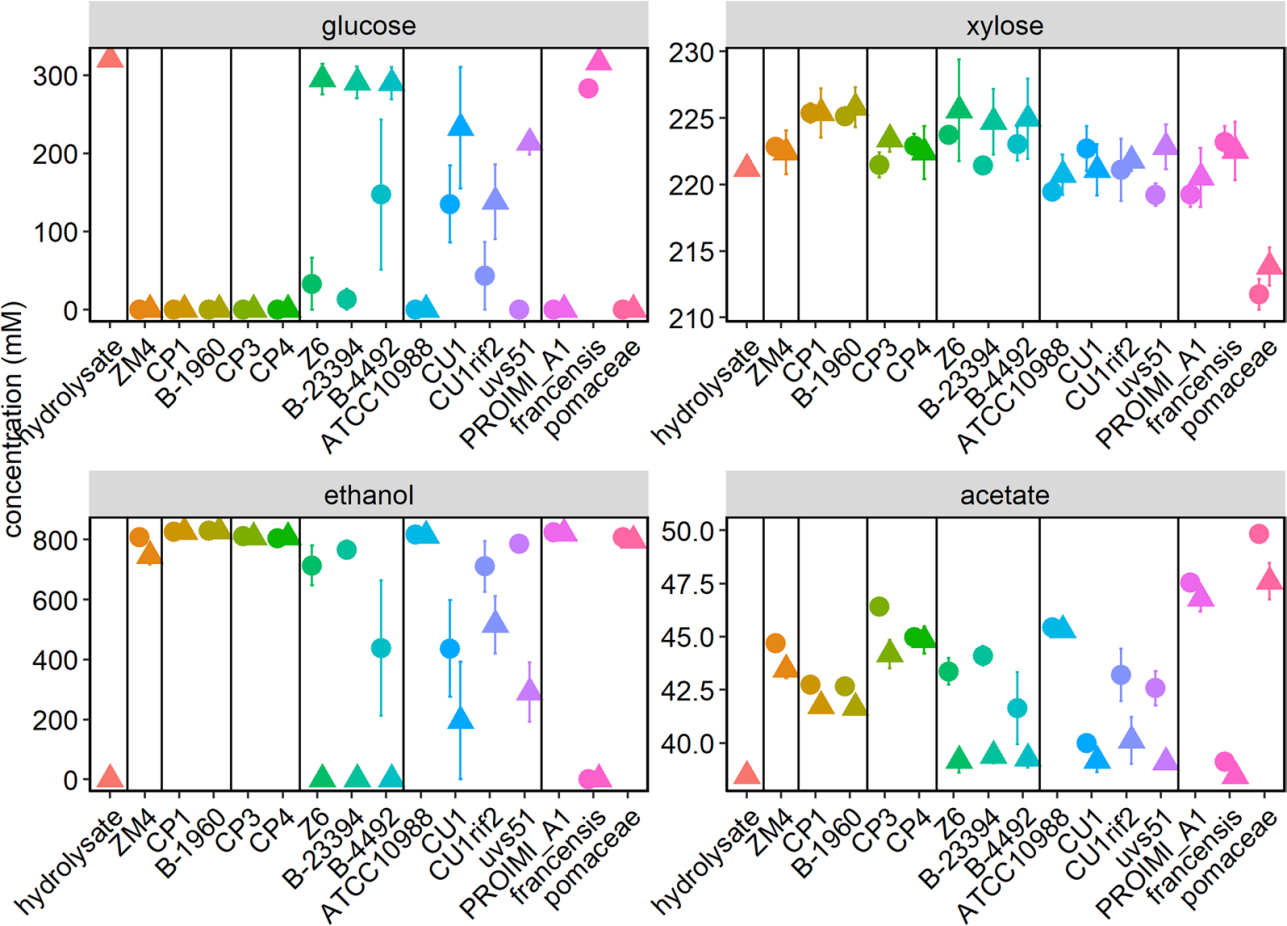
Metabolite analysis of *Zymomonas* grown in hydrolysate. Supernatants from the cultures described in Fig. 4 and the initial hydrolysate were analyzed by HPLC for a glucose, b xylose, c ethanol, and d acetate concentrations. Cultures were inoculated to a starting OD_600_ of 0.1 (triangles) or 0.5 (circles). Points represent the average of three biological replicates and error bars represent standard error

Because of significant costs associated with purifying sugars from lignocellulosic materials, ideal biofuel-producing strains should have the capability to grow directly in lignocellulosic hydrolysates. However, these hydrolysates can be stressful environments for microbes, due to high sugar concentrations, lignotoxins such as furfural or phenolic aldehydes, and low pH due to organic acids [29–31]. Therefore, we measured how well each *Zymomonas* strain tolerated lignocellulosic hydrolysate during anaerobic growth. All strains except the *francensis* subspecies were able to grow in lignocellulosic hydrolysate, although some required a relatively dense inoculum (initial OD_600_ = 0.5) to enable growth and ethanol production (Figs. 4 and 5). The requirement of some strains for a dense inoculum to grow may reflect higher sensitivity of these strains to toxins present in hydrolysate. Previous work with *Escherichia coli* demonstrated that increased inoculation density improved growth in the presence of furfural and phenolic aldehydes [32]. Strain ZM4 grew to a higher final OD_600_ than any of the other strains.

The Drainas group provides interesting comparisons to examine genes relevant to hydrolysate tolerance because of the variation in hydrolysate tolerance despite very similar genomes. Within the Drainas group, the derivatives of ATCC10988 did not grow well in hydrolysate and required a high inoculum, unlike their parent strain. CU1 and CU1rif2 could not consume all glucose in the hydrolysate even at the higher inoculum level. These strains’ previously documented intolerance of high glucose concentrations may explain the poor growth of the mutant strains on hydrolysate (6% glucose in the hydrolysate used here). Genome comparison between the four strains reveals that all three derivatives lost a set of 41 genes present in the parent strain (Additional file 1: Table S4). Of these, 37 were annotated as hypothetical proteins, 3 had general protein family annotations and one was annotated as a ribosomal protein. It seems likely that some of these 41 genes contribute to glucose tolerance, but at the current level of annotation of the *Zymomonas* genomes, it is not possible to propose a mechanism. These 41 genes are good candidates for future investigation of glucose tolerance in *Zymomonas.*

ZM4, the CP1 group, the CP4 group, ATCC10988, PROIMI A1, and *pomaceae* were able to consume all glucose in the hydrolysate and convert it to ethanol with high efficiency, regardless of inoculation density (Fig. 5). These strains also produced 2 to 8 mM acetate during growth on hydrolysate (initial hydrolysate contained 38.45 ± 0.05 mM acetate). Strains in the Z6 group consistently required a high inoculum to grow in hydrolysate and convert glucose to ethanol in this condition. One possible cause of the lower hydrolysate tolerance of strains in the Z6 group is that none of the strains contain a homolog of ZM4 ZM0101. This locus encodes a NAD-dependent epimerase/dehydratase and is important for hydrolysate tolerance in ZM4 [30]. The *francensis* subspecies appeared completely intolerant of hydrolysate and did not grow or significantly ferment glucose at either inoculum level.

All strains except the *pomaceae* subspecies were incapable of consuming xylose in the hydrolysate. The *pomaceae* subspecies consumed ~ 10 mM or ~ 4.7% of the xylose in the hydrolysate. However, it did not produce more ethanol than ZM4 and rather produced slightly more acetate. Significant efforts have been made to engineer ZM4 and CP4 to ferment xylose, therefore a native xylose fermentation pathway in the *Zymomonas* genus would be of significant interest in the field [33–35]. Although the *pomaceae* genome contains over 200 genes that do not have homologs in the other *Zymomonas* genomes, none of these could be clearly linked to xylose metabolism via current annotations. It is also possible that the xylose consumption phenotype results from changes in regulation of genes found in other organisms, considering that ZM4 can be evolved for xylose utilization without expression of heterologous genes [35]. Although the *pomaceae* subspecies in general does not have optimal characteristics as a biofuel producer, its native xylose utilization capability should be explored further.

### Isobutanol tolerance

**Fig. 6:**
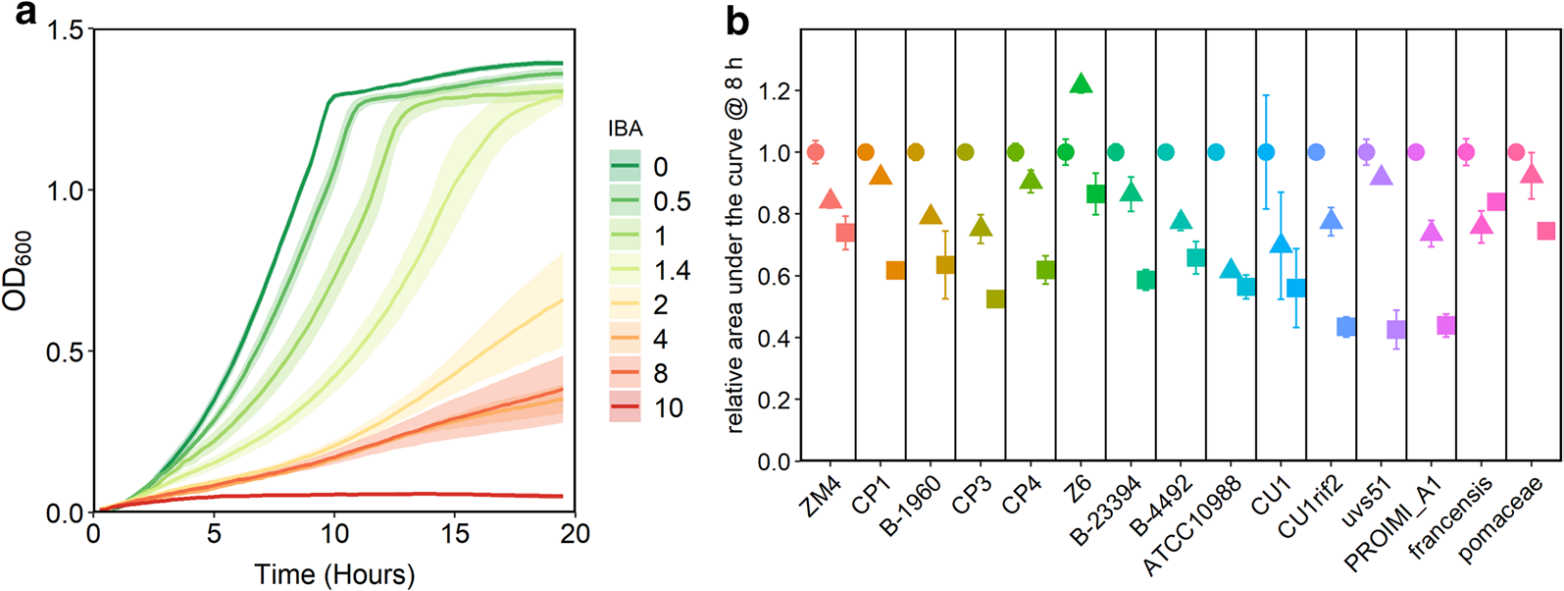
IBA tolerance of *Zymomonas* strains. **a** Growth of ZM4 in rich medium in the presence of 0 to 10% (v/v) IBA. **b** growth of *Z. mobilis* strains in rich medium in the presence of 0.0% (circles), 0.5% (triangles), or 1.0% (squares) IBA. AUC, calculated after 8 h of growth, with no IBA added is set to 1.0 for each strain and relative AUC is shown for conditions with IBA added. Points or lines represent the average of three biological replicates and error bars or ribbons represent standard error

Isobutanol (IBA) is an advanced biofuel being pursued for production in several platforms [36–38]. Therefore, we tested the tolerance of the *Z. mobilis* strains to this compound. We first determined the half maximal inhibitory concentration (IC_50_) of IBA with strain ZM4 to develop a protocol for assaying IBA tolerance. We grew ZM4 with a range of IBA concentrations and calculated area under the curve after 8 h of growth (AUC_8_). We used the AUC values to calculate an observed IC_50_ of ~ 1% for ZM4 grown in rich medium in anoxic conditions (Fig. 6a). We then compared the tolerance of all strains to 0.5% and 1.0% IBA by measuring the AUC at 8 h (Fig. 6b). All strains were able to grow in the presence of 1% IBA, with AUC_8_, ranging from ~ 40 to 80% of the control without isobutanol. Strains ZM4, Z6, *francensis*, and *pomaceae* showed the highest relative growth with 1% IBA, although the overall growth of the *francensis* and *pomaceae* strains was low at 8 h (Additional file 1: Figure S2). Strains in other groups showed similar tolerance to IBA with Drainas group being the least tolerant. Considering the relatively small differences in isobutanol tolerance, it was not possible to connect specific genes with improved isobutanol tolerance at this time. Because there was an unexpected increase in AUC_8_ for strain Z6 growth with 0.5% isobutanol, we repeated growth of ZM4 and Z6 with isobutanol in culture tubes. We observed that Z6 did not actually have increased growth with isobutanol and the result in the initial experiment was likely due to an inoculation error (Additional file 1: Figure S3).

### Genome methylation analysis and DNA defense systems

The new genomes presented here were sequenced using a PacBio platform that also detects methylation at each base. The methylation sites for the new genomes were analyzed to predict which types of DNA methylation machinery are present in each strain. All 9 of the newly sequenced genomes showed greater than 99% methylation at GANTC sites, characteristic of the CcrM (Cell cycle regulated Methyltransferase) system, which has been well-studied in *Caulobacter crescentus* and other α-proteobacteria. This methylation pattern is consistent with prior REBASE predictions for *Zymomonas* based on the presence of CcrM system genes [39]. The modification methylase (ZMO1005) has been found in all 17 sequenced genomes, with amino acid identity to the ZM4 gene of 99–100% in subspecies *mobilis*, 95% in *francensis*, and 82% in *pomaceae*. CcrM is involved in cell cycle regulation, including transcriptional regulation of cell-cycle control genes, and GANTC sites are overrepresented in intergenic regions in *C. crescentus* [40]. Further study of the *Z. mobilis* CcrM system is warranted, considering that recent analysis suggests that *Z. mobilis* may contain up to 100 copies of the genome per cell [30, 41].

Beyond the GANTC methylation site, several methylation patterns consistent with restriction–modification (R–M) systems were detected (Table 4). Sites that were methylated with a frequency greater than 99.9% were usually associated with Type I or Type II R–M systems. In some strains, a few highly similar sequences were detected. These are denoted as ‘variable’ in Table 4, and all sequence variants are provided in Additional file 1: Table S5. Methylated sequences were shared within the groups of strains (at least 4 within the Drainas group, and two within the Z6 group), probably resulting from the activity of *hsdM* methylases on the homologous plasmids described above. Some of the Type I methylation sequences were predicted earlier, but only for a few of the previously sequenced genomes (CP4, ZM4 and *pomaceae*, REBASE [39]).Thus, our methylation analysis together with annotation of Type I R–M genes to specific plasmids is an important addition to the current knowledge of restriction–modification systems in *Z. mobilis*.

**Table 4:** Methylation sites for R–M systems in newly sequenced *Zymomonas* strains

Sequence	Type	Strains	Group	% mod	Notes
cCwGg	m4C	CP1		99.6	
cAcnnnnnaTya	m6A	CP1		100	
rgAtcy	m6A	CP3		98.6	
craAnnnnnncTc	m6A	CP3		100	
rcgcAg	m6A	B-23394, B-4492	Z6	≥ 99.9	
gcAnnnnnncTga	m6A	B-23394, B-4492	Z6	100	
gcAgnnnnnnrta	m6A	CU1, CU1rif2, *uvs*51	Drainas	100	
tAynwnnnnctgc	m6A	CU1, CU1rif2, *uvs*51	Drainas	100	variable in CU1rif2
bgcAnnwnnntgct	m6A	CU1, CU1rif2, *uvs*51	Drainas	100	different in CU1rif2
agcAnnnnnntgc	m6A	CU1, CU1rif2, *uvs*51	Drainas	100	variable in *uvs*51
Ccggtgncar	m4C	PROIMI A1		39.1	
gagntCcnntnnnnnaw	m4C	PROIMI A1		92.9	
cTgcAg	m6A	*francensis*		100	PstI

Capital letters indicate the site of methylation. Methylated bases were detected during sequencing by correlating base incorporation kinetics with base modifications [42]. The methylation motifs were identified using motifMaker software (https://github.com/bioinfomaticsCSU/MultiMotifMaker)

Beyond the Type I and Type II R–M systems detected by methylation analysis, *Zymomonas* strains have additional DNA defense mechanisms in the form of Type IV restriction and CRIPSR/Cas systems. In 2011, Kerr et al*.* [43] identified a putative Type IV restriction endonuclease coding gene, *mrr* (ZMO0028) in ZM4, based on conserved domains with known Mrr proteins using BLASTp. Inactivation of *mrr* by insertion of a chloramphenicol resistance gene (*cam*) resulted in increased transformation efficiency in *Z. mobilis* ZM4, suggesting that the Mrr protein was responsible for cleaving foreign DNA [43, 44]. Mrr homologues were found in all sequenced genomes except subspecies *pomaceae* and are all chromosomally located. Sequence identity to the ZM4 gene was 100% with Drainas group, NCIMB 11163 and CP1, 99% with PROIMI A1, 97% with CP4 and 82% with *francensis*.

CRISPR systems have also been detected in *Zymomonas* previously. *Z. mobilis* ZM4 has a functional Type I-F CRISPR/Cas system, with Cas3 nuclease/helicase [3]. This CRISPR system has been successfully utilized for genome editing in ZM4 [45]. We observed Cas3 homologs on the chromosomes of all sequenced genomes of subspecies *mobilis*. Sequence identity with ZM4 Cas3 is 98% in the CP1 group, PROIMI A1 and NCIMB 11163, 97% with the CP4 and Drainas groups, and 95% with the Z6 group. *Z. mobilis pomaceae* appears to have Type I-E and I-C CRISPR systems on the chromosome and a 37 kb plasmid, respectively [3]. *Z. mobilis francensis* has many genes associated with CRISPR systems, but no homolog to the Cas3 of ZM4 has been found.

### Electroporation and conjugation efficiency

To begin to link the presence of DNA defense systems with physiology, we measured the efficiency of electroporation and conjugation of a plasmid into each of the *Z. mobilis* strains and normalized the efficiency to that of ZM4 (Table 5). We utilized a plasmid (pRL814) that was previously constructed from standard synthetic biology parts for use in *Z. mobilis* [46, 47]*.* pRL814 carries a spectinomycin resistance cassette and an IPTG-inducible *gfp*. We found that most strains were not easily transformed with this plasmid by electroporation. We successfully obtained colonies for only 5 strains, ZM4, Z6, B-4492, CP4, and CU1*rif*2. Two strains in the Z6 group had slightly higher electroporation efficiencies than ZM4, while CP4 and CU1*rif*2 had significantly lower electroporation efficiencies. Conjugation of the same plasmid from *E. coli* WM6026 into these strains was more successful. We obtained transconjugants carrying the plasmid for 12 out of 15 strains tested. In the case of conjugation, ZM4 clearly had the highest efficiency, with all other strains having a 10- to 1000-fold lower electroporation efficiencies. Overall, the ZM4 and Z6 groups were the most amenable to DNA uptake, with Z6 strains having slightly higher electroporation efficiency and ZM4 having significantly higher conjugation efficiency. We did not successfully introduce a plasmid into the *francensis* and *pomaceae* strains by either method. Specific method development for these subspecies is likely necessary to enable their engineering. Recent efforts to enhance the transformability of ZM4 by removing restriction–modification systems have been highly successful and a modified ZM4 strain has been developed with 3 R–M systems and the CRISPR–Cas system inactivated. This strain has a much higher conjugation efficiency than the parent strain with several plasmids tested (personal communication, Patricia Kiley, University of Wisconsin, Madison). Overall, the low conjugation and electroporation efficiencies we observed are consistent with the large number of genome defense systems detected in all genomes. Because a high level of DNA defense appears to be a common trait across the genus, further modification to improve ZM4 as a chassis appears to be a more promising strategy than screening for strains with a higher natural competency.

**Table 5:** Electroporation and conjugation efficiencies of each strain, relative to ZM4

Group	Strain	Electroporation	Conjugation
ZM4	ZM4	1	1
CP1	CP1	n.d	n.d
	B-1960	n.d	7 × 10^–2^
CP4	CP3	n.d	5 × 10^–3^
	CP4	0.01 ± 0.01	1 × 10^–2^
Z6	B-23394	n.d	3 × 10^–4^
	Z6	2.15 ± 0.77	9 × 10^–3^
	B-4492	4.78 ± 2.16	1 × 10^–3^
Drainas	ATCC 10988^ T^	n.d	1 × 10^–3^
	CU1	n.d	5 × 10^–3^
	CU1 *rif*2	0.25	4 × 10^–4^
	*uvs*51	n.d	2 × 10^–2^
None	PROIMI A1	n.d	2 × 10^–3^
	*francensis*	n.d	n.d
	*pomaceae*	n.d	n.d

Efficiency of electroporation for ZM4 was 15 CFU per 1 µg of pRL814 DNA. Efficiency of conjugation was 1.25 × 10^–4^ calculated as number of spectinomycin-resistant colonies divided by number of colonies on plates without spectinomycin

*n.d.* not detected

### Variability in cellulose synthase operon and promoter

**Fig. 7:**
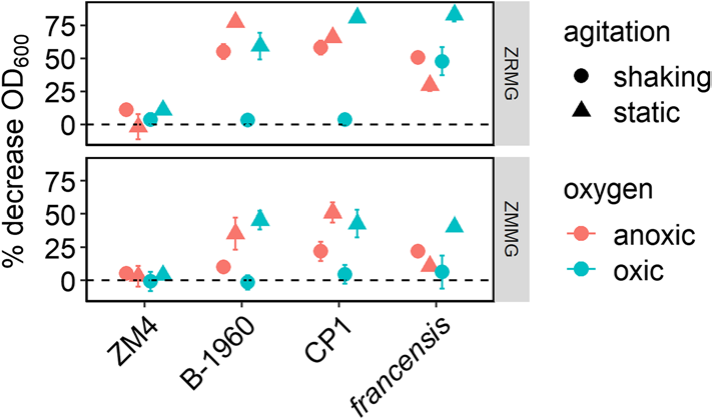
Flocculation of *Zymomonas* strains. Identical volume of the same colony resuspended in ZMMG was used to inoculate 5 ml of ZMMG or ZRMG. Cultures were grown for 48 h statically or with shaking in aerobic or anaerobic conditions as described in “Materials and methods”. After this time flocs were dispersed by vortexing, and OD_600_ was measured by removing 100 μl from the top of the culture to 0.9 ml of medium immediately and after 45 min of standing on a bench. Results shown are average of three independent experiments. Points represent the average of three biological replicates and error bars represent standard error

*Z. mobilis* strains have a tendency to flocculate, a trait that can be beneficial for bioprocessing [20, 48], but challenging for genetic modification and other laboratory protocols [49]. Floc formation by *Z. mobilis* ZM4, CP4 and their flocculating variants is driven by the production of extracellular cellulose fibrils, which become entangled and bind cells into flocs up to 4 mm in length [50, 51]. Several potential flocculation triggers have been reported, including high ethanol concentration, minimal medium, and oxygen, but overall, regulation of flocculation remains unclear [52, 53]. We identified three strains with naturally flocculating phenotypes: two strains in the CP1 group, and *Z. mobilis* subspecies *francensis* (Fig. 3c). To better understand the regulation of floc formation, we compared flocculating strains and ZM4 under a range of growth conditions, varying oxygen availability, shaking, and medium type (Fig. 7). The final OD_600_ of cultures grown in different conditions is shown in (Additional file 1: Figure S4. To measure flocculation, we fully resuspended stationary phase cultures by harsh vortexing, allowed them to re-flocculate and settle for 45 min, then measured the decrease in OD_600_ in the top 100 µL after settling. A greater % decrease in OD_600_ indicates more settling, and therefore, a greater degree of flocculation. Although PROIMI A1 was previously described as a flocculating strain, it formed only delicate, easy-to-disperse flocs under these experimental conditions, therefore it was not included in this analysis. We observed that ZM4 exhibited little flocculation under any tested condition, although flocculation has been observed for this strain previously [53]. Overall patterns of flocculation were similar between the other three strains, with increased flocculation in rich medium and static cultures. Oxygen did not appear to be a major driver of flocculation, except in the case of the *francensis* subspecies, for which oxygen exposure promoted flocculation. The greater effect of mechanical disruption than oxygen or medium suggested that the flocculating phenotypes were constitutive and probably determined by major changes in cellulose synthesis rather than more subtle changes at the regulatory level. To ensure that floc formation of all strains was due to cellulose fibril synthesis, we also treated cultures of CP1, B-1960, and *francensis* with cellulase and found that this completely dispersed the flocs, indicating that all strains tested here use the same flocculation mechanism as other *Zymomonas* strains (data not shown).

Cellulose synthesis is required for floc formation in *Z. mobilis* ZM4, CP4, and flocculating variants and production of cellulose fibrils is performed by the cellulose synthase complex encoded by the *bcs* operon [50, 51]. Strains bearing Tn5 insertions in *bcs* genes or with the entire operon deleted are not able to form flocs during aerobic growth in rich or minimal media [30, 49, 53]. Several highly flocculent strains have been described previously: PROIMI A1, ATCC 31822 (ZM401) and B-12526, a flocculating derivative of CP4. However, little work has been done to identify the genetic differences leading to the flocculating phenotype. Using Multiple Sequence Alignment (Clustal Ω), we compared *bcs* operons and their regulatory regions for 18 genomes to find differences between flocculating and non-flocculating strains. We added ZM401, which was not included in the general genome comparison, to this analysis.

Two transcription start sites (TSS) for the *bcs* operon at − 26 and − 23 have been identified upstream of ZMO1080 in ZM4 [54]. We identified and analyzed regulatory elements upstream of the TSS (Additional file 1: Figure S5) and found that a 177-nt region upstream of ZMO1080 was mostly invariable within *Z. mobilis mobilis* strains. Single nucleotide substitutions in the Z6 group seem irrelevant for flocculation and a single-nucleotide deletion in PROIMI A1 is unlikely to affect regulation (Additional file 1: Figure S5C). Subspecies *francensis* and *pomaceae* showed significant variability from *Z. mobilis mobilis* and each other within the region. TSSs of the *bcs* operon in these strains must be identified separately. Overall, it appears unlikely that differences in the promoter region contribute to differences in flocculation between strains.

There are 6 genes in the *bcs* operon of *Z. mobilis*: two coding for hypothetical proteins (ZMO1080, ZMO1081) and four genes with known functions (*bcsABCZ*, ZMO1083–1086). BcsA encodes a catalytic subunit anchored in the inner membrane with the catalytic center and regulatory PilZ domain in the cytosol. BcsA requires c-di-GMP bound to the PilZ domain for activity. BcsB is a periplasmic subunit anchored in the inner membrane. The BcsAB complex is found in all bacterial cellulose synthase operons and is indispensable for cellulose synthesis. BcsC is composed of an N-terminal periplasmic domain and C-terminal β-barrel forming a channel in the outer membrane for cellulose secretion. The N-terminal domain has multiple TPR (tetratricopeptide repeat) domains and is thought to organize function of the entire complex. Finally, BcsZ is a gluconase, which is required for proper formation of fibrils. ZMO1080 and ZMO1081 are highly conserved within *Zymomonas mobilis* and ZMO1081 shares homology with *bcsQ* from other Proteobacteria. ZMO1080 appears to be specific for *Z. mobilis*. Given their high conservation, these two genes are likely important for cellulose synthase function, but their roles have yet to be determined.

Unlike the promoter regions, *bcsA* genes were much more variable within *Zymomonas*. Clustal Ω alignment revealed significant length and sequence variability of Bcs proteins in *pomaceae* and *francensis* compared to *Z. mobilis mobilis* and the *bcsA* gene was not annotated in *pomaceae*. Thus, although we identified *francensis* as a flocculating strain, we were not able to attribute this phenotype to any specific gene within the *bcs* operon. Protein alignment of BcsA within the *mobilis* subspecies identified differences specific for one or more of the flocculating strains (Additional file 2: Figure S6). In PROIMI A1, a 465-nucleotide deletion at the junction of *bcsA* and *bcsB* results in truncated forms of both proteins. Adding the sequence of BcsA from *Rhodobacter sphaeroides* (for which the 3D crystal structure has been resolved [55]) to the alignment showed that the truncated BcsA lacks part of the PilZ domain that participates in binding c-di-GMP (Additional file 3: Figure S7). It seems likely that activity of cellulose synthase in PROIMI A1 has been deregulated by this deletion, possibly resulting in the constant but weak floc formation.

Sequence comparison of 26 genes differentially expressed in ZM401 compared to ZM4, did not reveal any differences in *bcsA* or *bcsB* genes or their upstream regions [56]. However, in independent research Xia et al. [50] found a single-nucleotide deletion (T) in *bcsA* of ZM401 as compared to ZM4 and suggested that *bcsA401* was fused to the upstream gene. This would result in a longer protein with an additional transmembrane helix compared with BcsA of ZM4. Our protein alignment of BcsA from ZM401 to other *Z. mobilis mobilis* strains showed a protein of the same length as in the other 12 strains (Additional file 2: Figure S6), revealing that rather than a T deletion in ZM401, there is a T insertion in ZM4. We confirmed one T insertion after A172 in *bcsA* from ZM4 by Sanger sequencing. Thus, ZM4 *bcsA* is a pseudogene as annotated in NCBI data base. It is uncertain why ZM4 can still flocculate in certain conditions. Because the additional T was inserted within a stretch of 8 thymidine nucleotides, it is possible that a ribosomal shift or RNA polymerase slippage can occasionally lead to synthesis of full-length BcsA in ZM4, thus providing some cellulose synthesis apparently sufficient to cause flocculation under specific conditions [53].

As described above, we found that two strains from subspecies *mobilis*, B-1960 and CP1, showed strong flocculation in all tested conditions. Protein alignment identified four single amino acid substitutions in BcsA that are specific for these two strains (Y408H, W452S, T519I, and F645V, Fig S5). The first three residues are conserved in *Zymomonas mobilis* and corresponding residues of *R. sphaeroides* BcsA (W418, L462, T528, Additional file 3: Figure S7) are located in transmembrane helices where they may assist in passage of cellulose through the inner membrane. It is possible that these mutations affect efficiency of cellulose export and result in increased flocculation. BcsB showed less variability across *Zymomonas* except that in B-12526 it is a pseudogene and in PROIMI A1 it is truncated from N-terminus (∆61 aa). As both these strains are naturally flocculating, it is possible that these changes contribute to flocculation.

In three flocculating strains (CP1, B-1960 and ZM401), we identified insertion of two serines in BcsC (S818–819), resulting in seven serines in a row versus four or five in other strains (Additional file 4: Figure S8). This stretch of serines is located within the periplasmic N-terminal part of BcsC. *P. aeruginosa* AlgK, a functional homolog of the BcsC N-terminal domain, organizes the function of the entire Bcs secretion complex [57]. It is possible that in *Zymomonas* the N-terminus of BcsC has a similar function and thus mutations within this region may lead to altered cellulose transport and subsequent flocculation.

Overall comparison of the Bcs operons and their promoter regions across 18 *Z. mobilis* strains showed that the promoter region is highly conserved while genes encoding cellulose synthase subunits display significant changes, including gene fusions, pseudogenes, and amino acid substitutions. Some changes within the *bcs* genes were specific to the flocculating strains, at least in one case affecting a regulatory domain, possibly leading to the flocculation phenotype appearing across all conditions. Our analysis suggests that flocculation is regulated primarily at the post-translational level in *Z. mobilis* and efforts to implement transcriptional control of this trait are not likely to be successful.

### Regulation of flocculation by diguanylate cyclases/phosphodiesterases

Bacterial Bcs complexes are positively regulated by cyclic di-GMP. Four GG(D/E)EF motif-containing diguanylate cyclases have been identified in ZM4 [53]. Two were previously linked to flocculation phenotypes; a Tn5 insertion in a diguanylate cyclase (ZMO0919) abolished flocculation of ZM4 in oxic minimal media while another diguanylate cyclase, ZMO1055 was upregulated in ZM401 as compared to ZM4. The single amino acid substitution A525V in the latter was proposed to cause flocculation [56]. We compared sequences of both genes and their promoter regions to see if there are any differences, which could lead to higher activation of BcsA in flocculating strains. There were two TSS reported at − 27 and − 38 of ZMO0919 gene and TSS at -119 of ZMO1055 [54]. Similar to the *bcs* operon, we found that the regulatory regions are highly conserved across subspecies *mobilis* with just one nucleotide deletion in two strains in the Drainas group. This mutation seems irrelevant to flocculation as we did not observe any differences between the four strains in this group. Other single-nucleotide substitutions are group-specific (Z6 or CP4) and not related to flocculation. The diguanylate cyclase genes were more variable; we found that in Drainas group ZMO1019 was split into two pseudogenes by insertion of a transposase. In NCIMB 11163, this locus acquired a stop codon and is annotated as a pseudogene. We also found a V430I substitution in flocculating strains CP1, B-1960 and PROIMI A1 (not shown). ZMO1055 showed even more variability within *Z. mobilis mobilis*. In the Z6 group and CP1 it is a pseudogene. We confirmed that the A525V substitution observed by Jeon et. al*.* [56] is unique for ZM401 as is A571T in PROIMI A1, both located in the N-terminus of the protein (not shown). Some diguanylate cyclases can substitute for each other, but recent findings show that only some regulate BcsA [58]. Our analysis shows that all flocculating strains have ZMO0919 while ZMO1055 is carrying mutations in PROIMI A1 and ZMO401 and is non-functional in the CP1 group (pseudogene). We speculate that the ZMO0919 diguanylate cyclase is more active in regulating BcsA and it is also possible that mutated versions of ZMO1055 acquired higher activities. We expect that future functional studies will shed additional light on the regulation of flocculation by diguanylate cyclases/phosphodiesterases.

## Conclusion

We described the present *Zymomonas* genomic diversity and compared bioenergy-relevant traits of 15 *Zymomonas* strains which revealed that strain ZM4 had the highest isobutanol tolerance, hydrolysate tolerance, and conjugation efficiency. Considering the low overall diversity observed within the genus, we propose that future efforts should focus on further developing one or a few strains via synthetic biology methods, rather than exploration of additional *Zymomonas* isolates. For example, several DNA defense systems were present in all strains, suggesting that low natural competence is a trait common to the entire genus. Based on our physiological characterization, we propose that ZM4 should be further developed into the model organism and platform strain for the genus. Some other strains had promising traits that should be studied further for potential transfer to ZM4, particularly the capability of the *pomaceae* strain to consume xylose in lignocellulosic hydrolysate.

## Methods

### Sequencing of *Zymomonas* genomes

The draft genomes of *Zymomonas mobilis* strains sequenced for this study were generated at the DOE Joint Genome Institute (JGI) using the Pacific Biosciences (PacBio) sequencing technology [59]. A > 10 kbp Pacbio SMRTbellTM library was constructed and sequenced on the PacBio Sequel platform. All general aspects of library construction and sequencing performed at the JGI can be found at http://www.jgi.doe.gov. The raw reads were assembled using HGAP [smrtlink/6.0.0.47835, HGAP 4 (0.2.1)] [60]. Additional file 1: Table S1 contains sequencing information for each genome, including filtered subreads, total bp, number of contigs and scaffolds in the final draft assembly, and genome size and input read coverage. DNA modification detection and motif analysis were performed using PacBio SMRT analysis platform (pbsmrtpipe.pipelines.ds modification motif analysis 0.1.0). Briefly, raw reads were filtered using SFilter, to remove short reads and reads derived from sequencing adapters. Filtered reads were aligned to the reference genome using BLASR (v5.3) [61]. Modified sites were then identified through kinetic analysis of the aligned DNA sequence data [42]. Modified sites were then grouped into motifs using MotifFinder2. These motifs represent the recognition sequences of methyltransferase genes active in the genome [62].

### Phylogenetics of *Zymomonas* genomes

#### Marker gene trees

A concatenated marker gene tree was constuctued using all 17 Zymomonas genomes together with 5 Sphingomonas genomes used as outgroup. The marker gene tree consists of 56 universal single copy genes and was produced by extracting these marker genes from each genome using hmmsearch (version 3.1b2). For each marker, alignments were constructed with MAFFT [63], trimmed with trimAl 1.4 [64], then concatenated into a single alignment. Maximum likelihood phylogenies were constructed with IQ-TREE [65], using the WAG substitution model and 1000 bootstraps. Trees were visualized with ggtree [66].

#### SNP trees

To generate the SNP tree, we followed a similar pipeline to the one found in [16], where each genome was aligned to a reference genome (highest quality genome in the set). SNPs were identified based on the Nucmer alignment output from the MUMmer package [67], concentated, followed by neighbor joinging tree construction. Trees were rooted on the two outlying genomes: *francensis* and *pomaceae*, and visualized with ggtree [66].

#### Genome clustering

Genome clustering was performed with two different approaches, the hierBAPS bayesian clustering analysis and clustering based on 99% average nucleotide identity (ANI). First for hierBAPS clustering, we employed a package integrated into R called RhierBAPS, Tonkin-Hill et al. 2018 which is an R implementation of the BAPS algorithm (Bayesian Analysis of Population Structure) [18], designed to identify subspecies genome clusters. Input for this software was the same whole genome alignment used in the SNP tree. Annother approach to unravel genetic relationships from the 17 Zymomonas genomes was to perform pairwise ANI clustering of all genomes at 99% similarity. To do this we used fastANI [68], filtered to an alignment fraction > 70%, then clustered into > 99% ANI clusters using mcl [69].

#### Orthologue clustering and analysis of gene content

Genes were called and annotated based on the Integrated Microbial Genomes (IMG) system at the DOE Joint Genome Instittute [70]. Naming of genes and contigs follows the IMG nomenclature. To cluster genes into gene families/orthologue groups, all genomes were passed into OrthoFinder [71]. Following clustering, genes were identified as belonging to the Core, Accessory or Singleton genomic components. For a gene to be considered core, it had to be identified in all 17 of the analyzed genomes. We merged the orthologue data together with the annotation data and provide as a supplemental file (Additional file 5: Table S6). A single rarefaction curve was generated based on the random resampling of the pool of unique gene family/orthologue groups, and a Venn diagram was created to assess shared and unique orthologues, both plotted in R.

### Phenotypic characterization of *Zymomonas* strains

#### Growth media and chemicals

LB medium (Miller, Accumedia) was used to grow *E. coli* strains. ZRMG medium (1% yeast extract, 2% glucose, and 15 mM KH_2_PO_4_) or ZMMG medium (1.0 g KH_2_PO_4,_ 1.0 g K_2_HPO_4,_ 0.5 g NaCl, 1.0 g (NH_4_)_2_SO_4_, 0.2 g MgSO_4_ × 7 H_2_O, 0.025 g Na_2_MoO_4_ × 2 H_2_O, 0.025 g FeSO_4_ × 7 H_2_O, 0.010 g CaCl_2_ × 2 H_2_O, 0.001 g calcium pantothenate, 20.0 g D-glucose per 1 L) was used to grow *Z. mobilis.* ZMMG medium was prepared from pre-autoclaved 10 × base solution (KH_2_PO_4_, K_2_HPO_4_, NaCl, (NH_4_)_2_SO_4_), 1000 × solutions of metal supplements and 10 × D-glucose. 1000 × concentrated calcium pantothenate was sterilized by filtration. All components were mixed, let stand for 45 min and filtered through a 0.22-μm filtering device. Solid media also contained 15 g of agar (Difco) per 1 L. Zymomonas recovery medium (ZRecM) contained 0.5% yeast extract, 1% tryptone, 0.025% MgSO_4_, 0.25% (NH_4_)_2_SO_4_, 0.5% glucose and 1.5 mM KH_2_PO_4_. When indicated, diaminopimelic acid (DAP) was added to final concentration of 0.1 mM. Spectinomycin was added to a final concentration of 100 μg/mL or 50 μg/mL for Z. *mobilis* and *E. coli* cultures, respectively. Ammonia fiber expansion-treated hydrolysate (6% Glucan AFEX Switchgrass Hydrolysate (ASGH) year 2010) was from Great Lakes Bioenergy Research Center, Madison, Wisconsin, and isobutanol was from Sigma-Aldrich.

#### Bacteria and plasmids

*Zymomonas mobilis* ATCC 31821 (ZM4) was from American Type Culture Collection (ATCC). NRRL B-1960, NRRL B-4286, NRRL B-4490, NRRL B-4492, NRRL B-14022, NRRL B-14023, NRRL B-23394, and NRRL B-23393 were obtained from USDA Agricultural Research Service Culture Collection (ARS). DSM 424, DSM 12495, DSM 12494, DSM 12497, DSM 14017, and DSM 18599 were from German Collection of Microorganisms and Cell Cultures (DSMZ). *E. coli* WM6026 (*lacIq rrnB3* ∆*lacZ*4787 *hsdR*514 ∆*araBAD*567 ∆*rhaBAD*568 *rph*- ∆*att*∆::pAE12 (∆*ori*R6K-*cat*:: Frt5), ∆ ∆*endA*::Frt *uidA*(∆MluI)::pir *att*HK::pJK1006(∆*ori*R6K-*cat*::Frt5; *trfA*::Frt) ∆*dapA*::Frt), [72] and broad host range plasmid pRL814 containing a spectinomycin resistance gene (*aadA1*) [46] were gifts from Dr. Patricia Kiley and Dr. Robert Landick (University of Wisconsin, Madison), respectively. pRL814 was propagated and purified from *E. coli* Mach1 (ThermoFisher Scientific).

#### Growth conditions

Growth kinetics was performed using a plate reader (BioTek Synergy H1) in anaerobic chamber (Coy Laboratory Products). Overnight cultures in 5 mL of ZRMG were inoculated from single colonies grown on ZRMG plates anaerobically. The cultures were grown statically to early stationary phase (OD_600_ around 1.0). The starter cultures were diluted to OD_600_ 0.1 with fresh ZRMG and wells of 96-well microtiter plates were filled with 150 μl of each culture in triplicates. The side wells of the plate were filled with ZRMG to minimize evaporation. Cells were grown at 30 °C with shaking for 20 h, and OD_600_ measurements were taken every 15 min. Doubling times were calculated from growth curves using the package “growthcurver” in R [28].

#### Conjugation efficiency

Conjugation was performed as described in Felczak et al. [47] except that the *E. coli* strain WM6026 was used as a donor of pRL814. Briefly, *Z. mobilis* strains were inoculated from a single colony and grown in 5 ml of ZRMG media statically in 14-ml tubes at 30 °C. WM6026 was grown overnight in LB supplemented with diaminopimelic acid (DAP) and spectinomycin at 37 °C. In the morning WM6026 culture was diluted to OD_600_ 0.2 in LB with DAP but without the antibiotic and grown at 30 °C to OD_600_ 1.0. Overnight culture of *Z. mobilis* was diluted to OD_600_ 1.0 and 0.9 ml was mixed with 0.9 ml of WM6026 at OD_600_ 1.0 in 2.0 ml Eppendorf tubes. Bacteria were centrifuged at 17,000 × g for 30 s in microcentrifuge at room temperature. Supernatant was decanted and the pellet let stand for 3 min. Loosened pellet was placed as a one drop in the center of plate containing solid ZRMG supplemented with 0.1 mM DAP and tryptone (10 g/ L). Plates were incubated overnight at 30 °C. Next day cells were scraped into 1.5-ml Eppendorf tube, 1 ml of ZRMG was added and cultures incubated for 5 h at 30 °C. After this time, 100 μl of undiluted or diluted (10 or 100 times) culture was spread on ZRMG with spectinomycin. 100 μl from serial dilutions in duplicate were spread on ZRMG plates without spectinomycin. Plates were incubated at 30 °C for 48 h for CFU or 4 days for exconjugants. Efficiency of conjugation was calculated as ratio of exconjugants to CFU/ ml.

#### Electroporation efficiency

pRL814 was introduced to *Z. mobilis* by electroporation**.** Electrocompetent cells were prepared as follows. 125 ml of ZRMG was inoculated from a single colony and cells were grown overnight in 125 ml bottles, loosely covered, at 30 °C until OD_600_ 0.4 (microaerobic conditions). Bottles were chilled on ice for over 30 min and bacteria were spun at 4000 × g for 15 min at 4 °C. Pellets were resuspended in equal volume of ice-cold MQ H_2_O and left on ice for 10 min. After this time, bacteria were spun as above. Washing was repeated one more time with half of the initial volume of ice-cold MQ H_2_O. After spinning as above cells were resuspended in 10 ml of ice-cold 10% glycerol and centrifuged at 4000 × g for 10 min at 4 °C. Final pellets were resuspended gently in 150 μl of ice-cold 10% glycerol and used immediately for electroporation as follows. 0.5–1 μg of plasmid DNA was mixed with 40 μl of electrocompetent cells. Electroporation was in 0.1-mm cuvettes in Micro Pulser (BioRad) set at EC-1. Cells were immediately resuspended in 1 ml of pre-warmed ZRecM and incubated statically at 30 °C for 5 h. 100 μl from undiluted or 10 times diluted electroporations was spread on ZRMG plates supplemented with spectinomycin. Plates were protected with parafilm from drying and incubated at 30 °C aerobically, for 4 days or longer until colonies appeared. Serial dilutions were plated on ZRMG without antibiotic to measure survival after electroporation. Efficiency of electroporation was calculated per 1 μg of plasmid DNA.

#### Growth on AFEX hydrolysate

pH of AFEX hydrolysate was adjusted to 5.8 with 10 M NaOH. The hydrolysate was filtered through 22-μm filter device (VWR) and stored in anaerobic chamber. *Z. mobilis* cultures were inoculated from fresh ZRMG plates and grown anaerobically in 10 mL of ZRMG overnight to OD 1–2 in anaerobic chamber. OD_600_ was measured and cells were concentrated to OD_600_ 10 by centrifuging cells at 8,000 rpm for 10 min (Sorval ST8, Thermo Scientific) at room temperature, followed by resuspending in appropriate volume of the hydrolysate. 5 mL of hydrolysate in Hungate tubes was inoculated to OD_600_ 0.1 or 0.5 under anaerobic chamber and tubes were capped and sealed. Tubes were incubated outside the chamber at 30 °C with shaking for 50 h. After this time, OD_600_ of 10 times diluted cultures was measured and samples were saved and stored at − 20 °C for analysis by HPLC**.**

#### Isobutanol tolerance

On cultures in 5 ml of ZRMG were inoculated from single colonies and grown overnight in anaerobic chamber to stationary phase. Cultures were diluted to OD_600_ 0.1 in fresh anaerobic ZRMG. Isobutanol was added to indicated final concentration, mixed and aliquots of 150 μl were loaded in triplicate to 96-well plate. Incubation was performed with shaking at 30 °C until growth reached saturation [18 h]. IC 50 for ZM4 was calculated from % growth inhibition by 0.25–10% IBA calculated as area under curve (AUC) after 8 h of incubation using online IC 50 calculator (https://www.aatbio.com/tools/ic50-calculator). Tolerance of *Z. mobilis* strains to 0.5% and 1% IBA was calculated similarly from AUC after 8 h.

#### Flocculation

Single colony from ZRMG plate grown anaerobically for 48 h was suspended in 200 μl of ZMMG. 40 μl was used to inoculate 5 ml of ZMMG or ZRMG in duplicate for subsequent aerobic or anaerobic growth. For static conditions, cultures were grown in 14-mL plastic tubes in an aerobic environment or an anaerobic chamber. For shaking conditions, cultures were grown with shaking at 275 rpm in Hungate tubes closed with a butyl rubber stopper or loosely covered with aluminum foil for anaerobic and aerobic growth, respectively. For the anaerobic condition, anoxic medium was dispensed into the tubes in an anaerobic chamber and the tubes were capped before removal to ensure an anoxic environment. Cultures were grown for 48 h. Cultures were vortexed until visible flocs disappeared (usually 5–10 s) and OD_600_ was measured immediately, and again after standing for 45 min on a bench. Each time 100 μl from the top of the culture was removed to 0.9 ml of media for measurement.

#### Chromosomal DNA purification

For the novo genome sequencing, *Z. mobilis* strains were grown to stationary phase in 100 ml of ZRMG at 30 °C, statically in anaerobic chamber. Cells were harvested by centrifugation at 6,000 rpm for 10 min at 4 °C (Sorval ST8, Thermo Scientific). Genomic DNA was purified as described by Neumann, et al*.* [73] with some modifications. Briefly, pelleted cells were washed with 100 ml of 0.1 M NaCl, centrifuged as above and resuspended in 5 ml of SET buffer (75 mM NaCl, 25 mM EDTA, 20 mM Tris pH 7.5). Lysozyme was added to final concentration of 1.0 mg/ml and incubated at 37 °C for 20 min. RNase A (Sigma), was added to a final concentration of 0.3 mg/ml and mixture was incubated for 20 min at 37 °C. After this time, SDS and Proteinase K were added to final concentrations of 1% and 1.0 mg/ml, respectively, and incubated at 55 °C for 2 h. After this time, 0.4 volume of 5.0 M NaCl was added to a clear, viscous cell lysate and mixed gently. An equal volume of chloroform was added and the mixture was incubated at RT for 30 min, on a swinging platform. After this time, phases were separated by spinning for 15 min at 8000 rpm at room temperature (Sorval ST8, Thermo Scientific). Clear water phase was collected into a new 15-ml conical tube and equal volume of isopropanol was added. The precipitated DNA was collected by wrapping around Pasteur pipette and submerged in 70% ethanol to wash out the residual water. DNA was transferred to Eppendorf tube, let dry at room temperature, resuspended in 200 μl of TE buffer (10 mM Tris HCl pH 8.0, 1 mM EDTA) and left in 4 °C overnight. Quality of the DNA was checked by electrophoresis on 0.7% agarose alongside lambda DNA standard (Thermo Scientific), and RNase A treatment was repeated when necessary followed by ethanol precipitation as described [74]. A_260_/A_280_ and A_260_/A_230_ was measured by nanodrop assay and the final concentration of DNA was determined by fluorometric method (Qubit). Finally, identity of the isolated DNA was confirmed by16S rRNA gene PCR using 27F and 1492R primers followed by Sanger-based sequencing of the PCR product [75].

## Supplementary Information

**Additional file 1**: **Figure S1**. Confirming the phenotypes of Drainas group strains CU1, CU1rif2 and uvs51. A. Glucose sensitivity was measured by growing strains ATCC10988 and its derivatives CU1, CU1rif2 and uvs51 statically in aerobic conditions at 30oC in ZRMG (2% glucose) or ZRMG supplemented with 10% glucose for 32 or 48 hours, respectively. B. Rifampicin resistance of strains CU1rif2 and uvs51 was confirmed by growing respective strains aerobically at 30oC on ZRMG plates without and with 10 µg/ml of rifampicin. **Figure S2**. 5 ml of ZRMG were inoculated from single colonies of each strain and grown overnight in anaerobic chamber to stationary phase. Cultures were diluted to OD600 0.1 in fresh anaerobic ZRMG. Isobutanol (IBA) was added to diluted cultures to final concentrations of 0.0, 0.5 and 1.0%, mixed and aliquots of 150 µl were loaded in triplicate onto 96-well plate. Incubation was performed with shaking at **30**°**C** until growth reached saturation (18 hours). Only the first 10 hours of the growth is shown because IC50 for ZM4 and percent of inhibition for all the strains were calculated from area under the curve after 8 hours of growth. **Figure S3**. Repeated growth of strains ZM4 and Z6 with isobutanol. Normalized OD600 after 9 hours of growth is shown for cultures grown with 0%, 0.5%, or 1% v/v isobutanol. **Figure S4**. Initial OD600 of cultures used for flocculation experiment described in Figure 7. Identical volume of the same colony resuspended in ZMMG was used to inoculate 5 ml of ZMMG or ZRMG. Cultures were grown for 48 hours statically in 14 ml plastic tubes in aerobic environment or in anaerobic chamber, or with shaking at 275 rpm in Hungate tubes closed or loosely covered with aluminum foil for anaerobic and aerobic growth, respectively. **Figure S5**. The promoter region of the cellulose synthase (bcs) operon. A. Z. mobilis ZM4 promoter consensus sequence. Reproduced exactly as shown in: (Vera et al., 2020). B. Regulatory elements of the bcs operon: TSS -23 and -26, and -10 and -35 elements are highlighted in yellow, the ZMO1080 start codon is highlighted in green. C. Clustal Ω alignment of the region upstream of the ZMO1080 start codon of 16 Z. mobilis mobilis strains. Pomaceae and francensis are not included for clarity. ZM4 regulatory elements are colored as in B and nucleotides different from consensus are highlighted in blue. A single nucleotide deletion is highlighted in red. **Table S1**. Genome statistics for Zymomonas strains sequenced for this study. (see separate Excel file). **Table S2**. Information on Zymomonas strains and genomes. A list of all strains that were used for physiological and/or genomic comparisons. Common names and reference numbers for the American Type Culture Collection (ATCC), USDA Agricultural Research Service Culture Collection (NRRL), and the German Collection of Microorganisms and Cell Cultures (DSMZ), and the original source of isolation are provided. Strains were grouped based on genome similarity (see text). Highlights indicate the strain designation used to refer to each strain throughout the text, figures, and subsequent tables. **Table S3**. Names, accession numbers and topology determination of plasmids analyzed in Table 2. (see separate Excel file). **Table S4**. Loci and predicted functions for 41 genes present in ATCC10988 but without detectable homologs in any of the 3 derivative strains in the Drainas group. **Table S5**. Full list of methylated sequences found in 9 de-novo sequenced Zymomonas mobilis genomes.

**Additional file 2**: **Figure S6**.Z. mobilis BcsA alignment (see figure in separate PDF). BcsA (ZMO1083) protein alignment was performed by Clustal Ω and visualized by ESPript 3.0 (Robert & Gouet, 2014). Pomaceae and ZM4 are not included as they were not annotated or annotated as pseudogene, respectively. In CP4 bcsA G629A substitution results in a stop codon (TAG); Met 42 is a start codon and protein is truncated from N-terminus. C-terminal truncation of PROIMIA1 BcsA is discussed in text. Residues with strict identity are in white on red. Threshold for high similarity was set at 0.7 and similar residues are shown in red and framed in blue. Weakly similar residues are shown in black.

**Additional file 3**: **Figure S7**. Z. mobilis BcsA alignment with secondary structures from Rhodobacter sphaeroides 4P02_A (see figure in separate PDF). BcsA (ZMO1083) was aligned with BcsA from R. sphaeroides using Clustal Ω and visualized by ESPript 3.0 (1). Secondary structures derived from R. sp. 3D structure (PDB 4P02) are shown at the top. α and π helices are shown in medium and small squiggles, β sheets as arrows and α or β turns as TT or TTT, respectively. Color code is as in Figure S5. β-16 and β-17 of R. sphaeroids are part of a PilZ domain binding c-di GMP.

**Additional file 4**: **Figure S8**. Z. mobilis mobilis BcsC alignment (see figure in separate PDF). BcsC (ZMO1085) alignment was performed as described in Figure S5 (BcsA). BcsC from pomaceae is a pseudogene and BcsC from francensis was omitted for clarity. BcsC of NCIMB11163 is fused in-frame with bcsZ. Only first residue of fused BcsCZ protein is shown. All colors are as in Figure S5.

**Additional file 5**: **Table S6**. Merged orthologue and annotation data.

## Data Availability

All raw and assembled genome sequences are publicly available under the accession numbers listed in Table 1. Methylation data can be found on JGI’s Genome Portal pages (https://genome.jgi.doe.gov/portal/). Raw phenotypic data are available upon request to the authors.
