# Supplementary material for: Zymomonas diversity and potential for biofuel production
Source: Biotechnol Biofuels. 2021 May 1;14:112. doi: 10.1186/s13068-021-01958-2 (PMC8088579; doi:10.1186/s13068-021-01958-2)
Supplement: Supplementary file 1 — Additional file 1: Figure S1. Confirming the phenotypes of Drainas group strains CU1, CU1rif2 and uvs51. A. Glucose sensitivity was measured by growing strains ATCC10988 and its derivatives CU1, CU1rif2 and uvs51 statically in aerobic conditions at 30oC in ZRMG (2% glucose) or ZRMG supplemented with 10% glucose for 32 or 48 hours, respectively. B. Rifampicin resistance of strains CU1rif2 and uvs51 was confirmed by growing respective strains aerobically at 30oC on ZRMG plates without and with 10 µg/ml of rifampicin. Figure S2. 5 ml of ZRMG were inoculated from single colonies of each strain and grown overnight in anaerobic chamber to stationary phase. Cultures were diluted to OD600 0.1 in fresh anaerobic ZRMG. Isobutanol (IBA) was added to diluted cultures to final concentrations of 0.0, 0.5 and 1.0%, mixed and aliquots of 150 µl were loaded in triplicate onto 96-well plate. Incubation was performed with shaking at 30°C until growth reached saturation (18 hours). Only the first 10 hours of the growth is shown because IC50 for ZM4 and percent of inhibition for all the strains were calculated from area under the curve after 8 hours of growth. Figure S3. Repeated growth of strains ZM4 and Z6 with isobutanol. Normalized OD600 after 9 hours of growth is shown for cultures grown with 0%, 0.5%, or 1% v/v isobutanol. Figure S4. Initial OD600 of cultures used for flocculation experiment described in Figure 7. Identical volume of the same colony resuspended in ZMMG was used to inoculate 5 ml of ZMMG or ZRMG. Cultures were grown for 48 hours statically in 14 ml plastic tubes in aerobic environment or in anaerobic chamber, or with shaking at 275 rpm in Hungate tubes closed or loosely covered with aluminum foil for anaerobic and aerobic growth, respectively. Figure S5. The promoter region of the cellulose synthase (bcs) operon. A. Z. mobilis ZM4 promoter consensus sequence. Reproduced exactly as shown in: (Vera et al., 2020). B. Regulatory elements of the bcs operon: TSS -23 and [file 13068_2021_1958_MOESM1_ESM.docx]

***Zymomonas* diversity and potential for biofuel production**

Magdalena M. Felczak^1^, Robert M. Bowers^2^, Tanja Woyke^2^, Michaela A. TerAvest^1^*

^1^Department of Biochemistry and Molecular Biology, Michigan State University, East Lansing, MI, USA, 48824

^2^U.S. Department of Energy Joint Genome Institute, Lawrence Berkeley National Laboratory,

Berkeley, CA, 94720, USA

Supplementary Figures

A.

B.


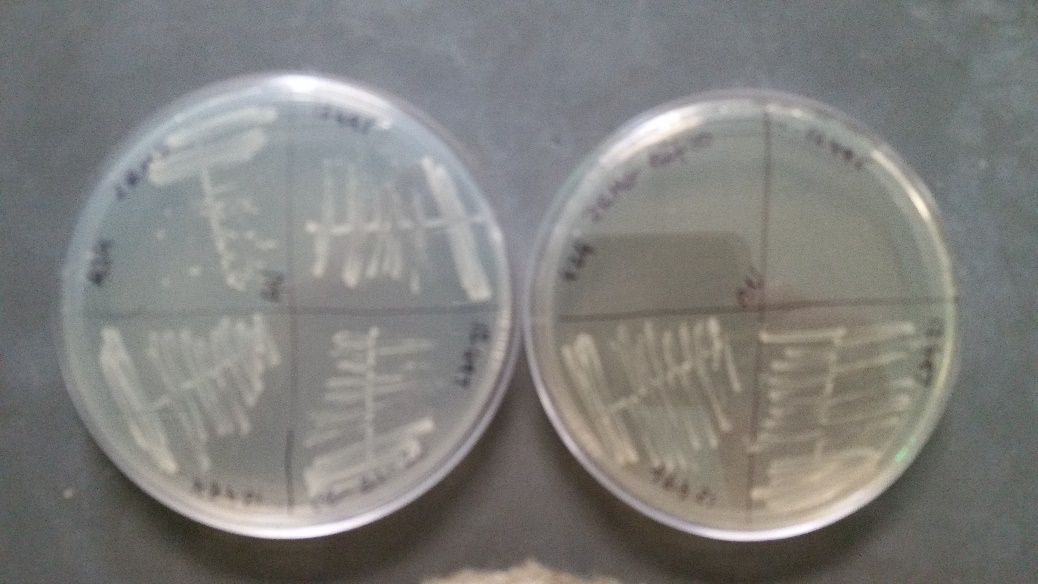


**CU1*rif*2**

***uvs*51**

**CU1**

**ATCC10988**

**ATCC10988**

**CU1**

***uvs*51**

**CU1*rif*2**

**-rif**

**+rif**

**Figure S1.** Confirming the phenotypes of Drainas group strains CU1, CU1rif2 and uvs51. A. Glucose sensitivity was measured by growing strains ATCC10988 and its derivatives CU1, CU1rif2 and uvs51 statically in aerobic conditions at 30^o^C in ZRMG (2% glucose) or ZRMG supplemented with 10% glucose for 32 or 48 hours, respectively. B. Rifampicin resistance of strains CU1*rif2* and *uvs*51 was confirmed by growing respective strains aerobically at 30^o^C on ZRMG plates without and with 10 µg/ml of rifampicin.


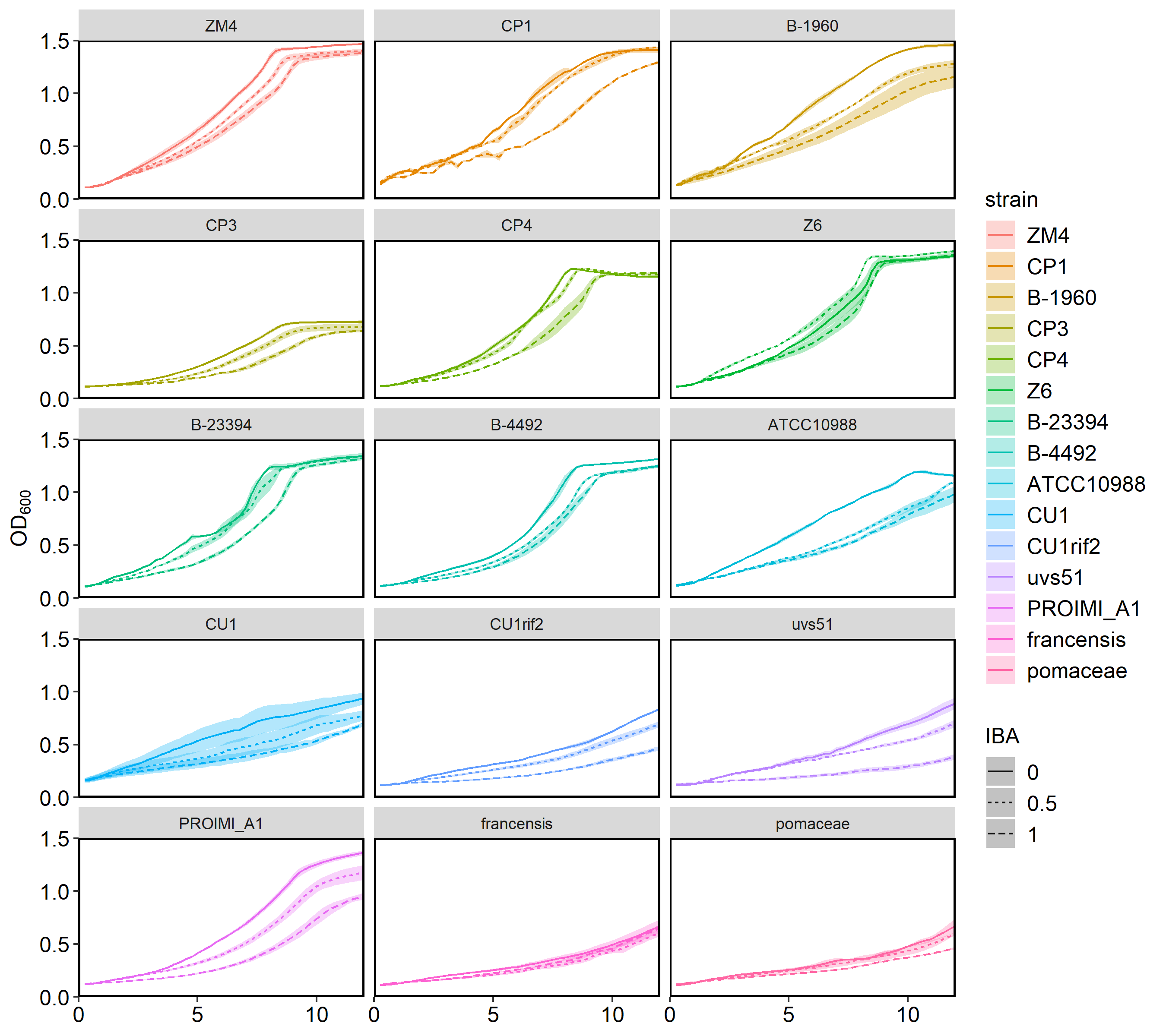


**Figure S2.** 5 ml of ZRMG were inoculated from single colonies of each strain and grown overnight in anaerobic chamber to stationary phase. Cultures were diluted to OD_600_ 0.1 in fresh anaerobic ZRMG. Isobutanol (IBA) was added to diluted cultures to final concentrations of 0.0, 0.5 and 1.0%, mixed and aliquots of 150 µl were loaded in triplicate onto 96-well plate. Incubation was performed with shaking at 30**°**C until growth reached saturation (18 hours). Only the first 10 hours of the growth is shown because IC50 for ZM4 and percent of inhibition for all the strains were calculated from area under the curve after 8 hours of growth.

**Figure S3.** Repeated growth of strains ZM4 and Z6 with isobutanol. Normalized OD_600_ after 9 hours of growth is shown for cultures grown with 0%, 0.5%, or 1% v/v isobutanol.


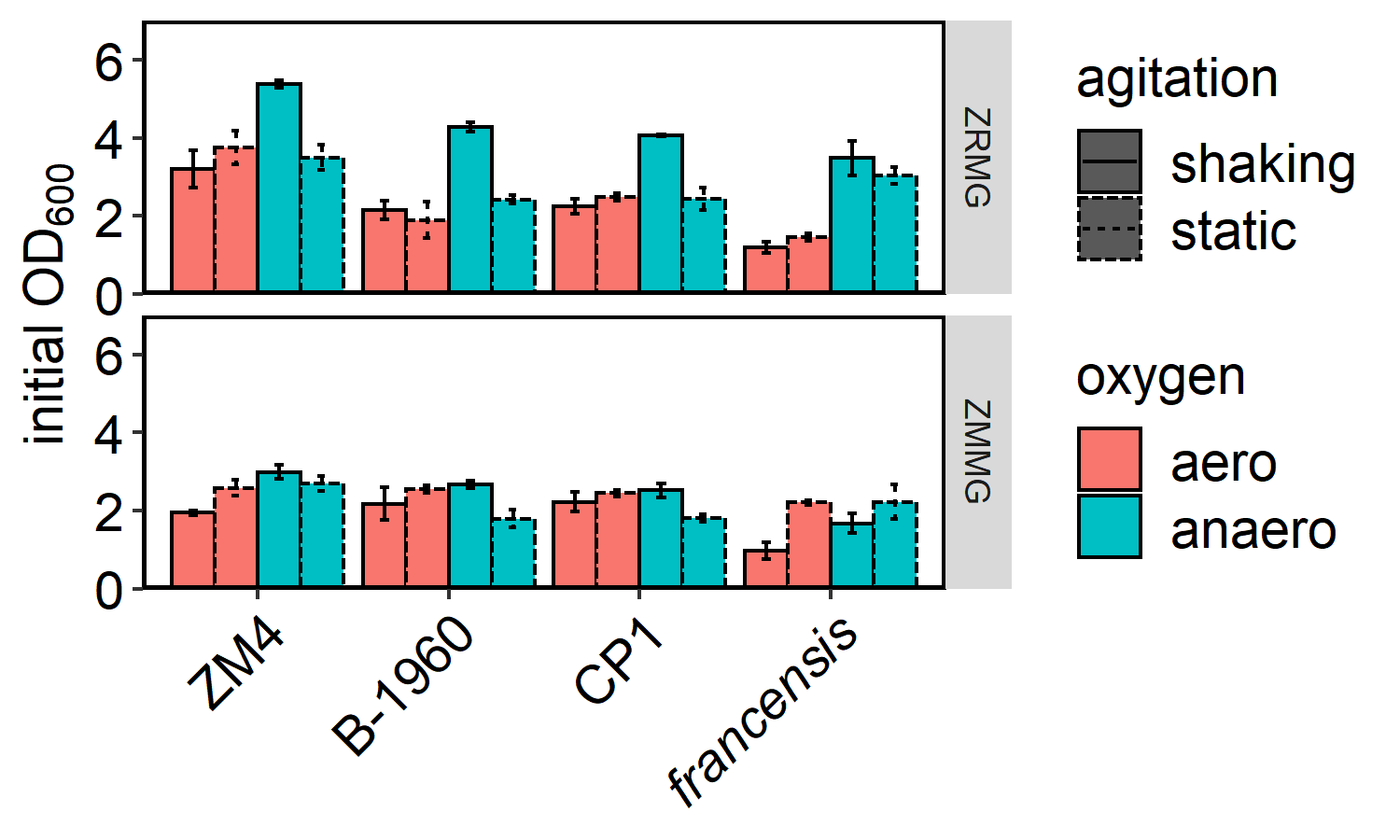


**Figure S4.** Initial OD_600_ of cultures used for flocculation experiment described in Figure 7. Identical volume of the same colony resuspended in ZMMG was used to inoculate 5 ml of ZMMG or ZRMG. Cultures were grown for 48 hours statically in 14 ml plastic tubes in aerobic environment or in anaerobic chamber, or with shaking at 275 rpm in Hungate tubes closed or loosely covered with aluminum foil for anaerobic and aerobic growth, respectively.


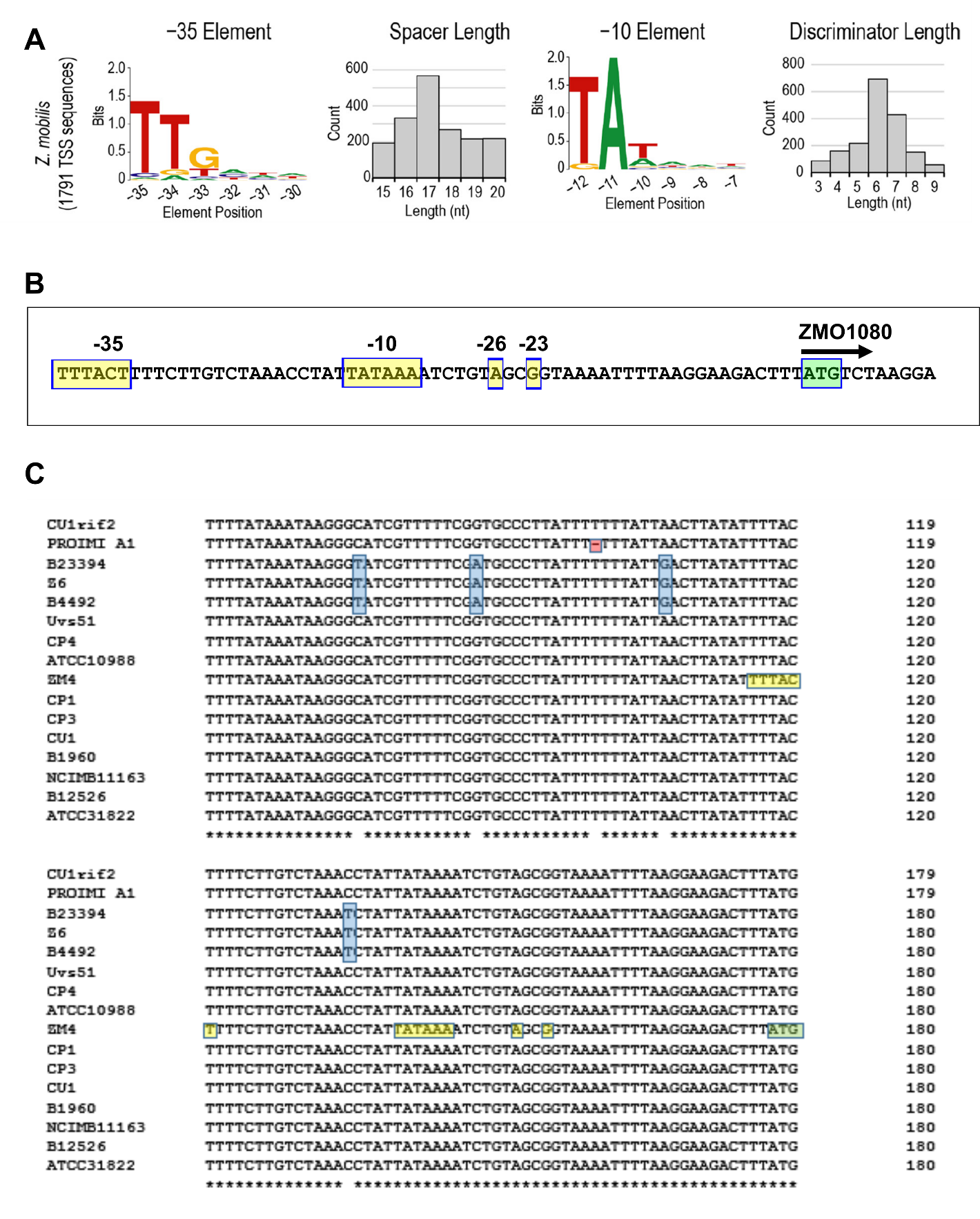


**Figure S5.** The promoter region of the cellulose synthase (*bcs*) operon. A. *Z. mobilis* ZM4 promoter consensus sequence. Reproduced exactly as shown in: (Vera et al., 2020). B. Regulatory elements of the *bcs* operon: TSS -23 and -26, and -10 and -35 elements are highlighted in yellow, the ZMO1080 start codon is highlighted in green. C. Clustal Ω alignment of the region upstream of the ZMO1080 start codon of 16 *Z. mobilis mobilis* strains. *Pomaceae* and *francensis* are not included for clarity. ZM4 regulatory elements are colored as in B and nucleotides different from consensus are highlighted in blue. A single nucleotide deletion is highlighted in red.

**Legend S6. *Z. mobilis* BcsA alignment (see figure in separate PDF).** BcsA (ZMO1083) protein alignment was performed by Clustal Ω and visualized by ESPript 3.0 (Robert & Gouet, 2014). Pomaceae and ZM4 are not included as they were not annotated or annotated as pseudogene, respectively. In CP4 *bcsA* G629A substitution results in a stop codon (TAG); Met 42 is a start codon and protein is truncated from N-terminus. C-terminal truncation of PROIMIA1 BcsA is discussed in text. Residues with strict identity are in white on red. Threshold for high similarity was set at 0.7 and similar residues are shown in red and framed in blue. Weakly similar residues are shown in black.

**Legend S7. *Z. mobilis* BcsA alignment with secondary structures from *Rhodobacter sphaeroides* 4P02_A (see figure in separate PDF).** BcsA (ZMO1083) was aligned with BcsA from *R. sphaeroides* using Clustal Ω and visualized by ESPript 3.0 (1). Secondary structures derived from R. sp. 3D structure (PDB 4P02) are shown at the top. α and π helices are shown in medium and small squiggles, β sheets as arrows and α or β turns as TT or TTT, respectively. Color code is as in Figure S5. β-16 and β-17 of *R. sphaeroids* are part of a PilZ domain binding c-di GMP.

**Legend S8.** ***Z. mobilis* *mobilis* BcsC alignment (see figure in separate PDF).** BcsC (ZMO1085) alignment was performed as described in Figure S5 (BcsA). *BcsC* from *pomaceae* is a pseudogene and BcsC from *francensis* was omitted for clarity. *BcsC* of NCIMB11163 is fused in-frame with *bcsZ*. Only first residue of fused BcsCZ protein is shown. All colors are as in Figure S5.

**Table S1. Genome statistics for *Zymomonas* strains sequenced for this study. (see separate Excel file)**

| **common** | **strain** | **IMG taxID** | **filtered subreads** | **total bp sequenced** | **contigs** | **scaffolds** | **genome size** | **read coverage** | **completeness** | **contamination** | **sequencing status** |
| --- | --- | --- | --- | --- | --- | --- | --- | --- | --- | --- | --- |
| uvs51 | DSM12494 | 2811994963 | 448,031 | 1,606,906,646 | 5 | 5 | 2,110,151 | 663.8X | 99.77 | 0.45 | permanent draft |
| CU1 | DSM12495 | 2811994958 | 380,528 | 1,427,626,539 | 3 | 3 | 2,074,423 | 571.8X | 99.55 | 0.00 | permanent draft |
| CU1rif2 | DSM12497 | 2811994957 | 239,289 | 1,104,643,071 | 4 | 4 | 2,112,663 | 457.8X | 100.00 | 0.45 | permanent draft |
| PROIMI A1 | DSM14017 | 2811994956 | 294,377 | 1,200,589,003 | 4 | 4 | 2,094,321 | 444.4X | 100.00 | 0.00 | permanent draft |
| CP1 | B-4286 | 2811994965 | 420,333 | 1,792,479,352 | 4 | 4 | 2,066,920 | 693.4X | 100.00 | 0.00 | permanent draft |
| CP3 | B-14022 | 2818991419 | 545,644 | 2,502,873,835 | 22 | 22 | 1,858,913 | 697.4X | 88.91 | 0.00 | permanent draft |
| francensis | DSM18599 | 2811994955 | 208,251 | 724,802,834 | 7 | 7 | 2,103,871 | 300.8X | 100.00 | 0.00 | permanent draft |
|  | B-23394 | 2811994876 | 346,953 | 1,446,870,957 | 4 | 4 | 2,023,540 | 615.5X | 100.00 | 0.00 | permanent draft |
|  | B-4492 | 2811994895 | 434,503 | 1,782,484,558 | 5 | 5 | 2,037,465 | 752.9X | 100.00 | 0.00 | permanent draft |

**Table S2. Information on *Zymomonas* strains and genomes.** A list of all strains that were used for physiological and/or genomic comparisons. Common names and reference numbers for the American Type Culture Collection (ATCC), USDA Agricultural Research Service Culture Collection (NRRL), and the German Collection of Microorganisms and Cell Cultures (DSMZ), and the original source of isolation are provided. Strains were grouped based on genome similarity (see text). Highlights indicate the strain designation used to refer to each strain throughout the text, figures, and subsequent tables.

| **group name** | **common** | **subsp.** | **ATCC** | **NRRL** | **DSMZ** | **original source** |
| --- | --- | --- | --- | --- | --- | --- |
| ZM4 | ZM4 | *mobilis* | ATCC31821 | B-14234 | NA | derivative or contaminant of CP4 |
| CP1 | none | *mobilis* | ATCC29501 | B-1960 | DSM473 | spoiled beer, Scotland |
|  | CP1 | *mobilis* | NA | B-4286 | NA | fermenting sugarcane juice, Brazil |
| CP4 | CP3 | *mobilis* | NA | B-14022 | NA | fermenting sugarcane juice, Brazil |
|  | CP4 | *mobilis* | NA | B-14023 | NA | fermenting sugarcane juice, Brazil |
|  | none | *mobilis* | NA | B-12526 | NA | mutant of CP4 (flocculating) |
| Z6 | Z6 | *mobilis* | ATCC29191 | B-4490 | DSM3580 | fermenting palm sap, Zaire |
|  | none | *mobilis* | NA | B-23394 | NA | fermenting palm sap, Zaire |
|  | none | *mobilis* | NA | B-4492 | NA | listed as identical to B-1960 |
| Drainas | type strain | *mobilis* | ATCC10988 | B-806 | DSM424 | fermenting agave sap, Mexico |
|  | CU1 | *mobilis* | NA | NA | DSM12495 | mutant of ATCC10988 |
|  | CU1rif2 | *mobilis* | NA | NA | DSM12497 | mutant of CU1 |
|  | uvs51 | *mobilis* | NA | NA | DSM12494 | mutant of CU1rif2 |
| none | PROIMI A1 | *mobilis* | NA | NA | DSM14017 | fermenting sugarcane juice, Argentina |
|  | NCIMB11163 | *mobilis* | NA | NA | NA | ale, England |
|  | AN0101 | *francensis* | NA | NA | DSM18599 | spoiled cider, France |
|  | none | *pomaceae* | ATCC29192 | B-23393 | DSM22645 | spoiled cider, England |

**Table S3.** **Names, accession numbers and topology determination of plasmids analyzed in Table 2.** (**see separate** **Excel file**)

| **Strain** | **Plasmid name** | **Size(kbp)** | **NCBI Accession #** | **Bridging Reads** | **Topology** | **Author/ NCBI update** |
| --- | --- | --- | --- | --- | --- | --- |
| ATCC 31821 (ZM4) | pZM32 | 32.8 | NZ_CP023716 |  |  | Yang 2018/ Jan 2020 |
|  | pZM33 | 33.0 | NZ_CP023717 |  |  | Yang 2018/ Jan 2021 |
|  | pZM36 | 36.5 | NZ_CP023718 |  |  | Yang 2018/ Jan 2022 |
|  | pZM39 | 39.3 | NZ_CP023719 |  |  | Yang 2018/ Jan 2023 |
| NRRL B-12526 | pZM1252601 | 33.9 | NZ_CP003710.1 |  |  | Chen 2012/Nov 2019 |
|  | pZM1252602 | 30.9 | NZ_CP003711.1 |  |  | Chen 2012/Nov 2019 |
|  | pZM1252603 | 37.1 | NZ_CP003712.1 |  |  | Chen 2012/Nov 2019 |
|  | pZM1252604 | 32.4 | NZ_CP003713.1 |  |  | Chen 2012/Nov 2019 |
|  | pZM1252605 | 32.8 | NZ_CP003714.1 |  |  | Chen 2012/Nov 2019 |
| NRRL B-14023 (CP4) | unnamed | 36.9 | NC_022901.1 |  |  | Kouvelis 2014/Feb 2020 |
|  | unnamed | 32.4 | NC_022902.1 |  |  | Kouvelis 2014/Feb 2020 |
|  | unnamed | 33.9 | NC_022913.1 |  |  | Kouvelis 2014/Feb 2020 |
|  | unnamed | 30.9 | NC_022903.1 |  |  | Kouvelis 2014/Feb 2020 |
|  | unnamed | 30.4 | NC_022910.1 |  |  | Kouvelis 2014/Feb 2020 |
| NCIMB11163 | pZA1001 | 53.4 | NC_013356.1 |  |  | Kouvelis 2009/Feb 2020 |
|  | pZA1002 | 40.8 | NC_013357.1 |  |  | Kouvelis 2009/Feb 2020 |
|  | pZA1003 | 4.5 | NC_013358.1 |  |  | Kouvelis 2009/Feb 2020 |
| ATCC 10988 | pZMOB01 | 32.5 | NC_017180.1 |  |  | Pappas 2011/Feb 2020 |
|  | pZMOB02 | 32.3 | NC_017183.1 |  |  | Pappas 2011/Feb 2020 |
|  | pZMOB03 | 31.7 | NC_017181.1 |  |  | Pappas 2011/Feb 2020 |
|  | pZMOB04 | 18.5 | NC_017184.1 |  |  | Pappas 2011/Feb 2020 |
|  | pZMOB05 | 4.0 | NC_017182.1 |  |  | Pappas 2011/Feb 2020 |
|  | pZMOB06 | 2.7 | NC_017185.1 |  |  | Pappas 2011/Feb 2020 |
| ATCC 29191 (Z6) | pZZ6.01 | 18.3 | NC_018146.1 |  |  | Desiniotis 2012/Aug 2019 |
|  | pZZ6.02 | 14.9 | NC_018147.1 |  |  | Desiniotis 2012/Aug 2019 |
|  | pZZ6.03 | 13.7 | NC_018148.1 |  |  | Desiniotis 2012/Aug 2019 |
| ATCC 29192 (*pomaceae*) | pZYMOP01 | 37.4 | NC_015715.1 |  |  | Kouvelis 2011/Feb 2020 |
|  | pZYMOP02 | 34.2 | NC_015716.1 |  |  | Kouvelis 2011/Feb 2020 |
| NRRL B-1960 | pZMO1960_1 | 34.5 | CP021791 |  |  | Chacon-Vargas /June 2017 |
|  | pZMO1960-1A | 1.7 | CP021792 |  |  | Chacon-Vargas /June 2017 |
| B-4286 (CP1) | pZMCP1 01 | 7.7 | NZ_VIVG01000003.1 | 0 | linear | TerAvest/ 2019 |
|  | pZMCP1 02 | 6.7 | NZ_VIVG01000002.1 | 0 | linear | TerAvest/ 2019 |
|  | pZMCP1 03 | 6.5 | NZ_VIVG01000004.1 | 0 | linear | TerAvest/ 2019 |
| B-14022 (CP3) | No | many | NZ_VIVT01000001.1 to NZ_VIVT01000022.1 | 0 | linear | TerAvest/ 2019 |
| B-23394 | pZM23394 01 | 33.5 | NZ_VFNW01000003.1 | 28 | circular | TerAvest/ 2019 |
|  | pZM23394 02 | 14.9 | NZ_VFNW01000004.1 | 4 | linear | TerAvest/ 2019 |
|  | pZM23394 03 | 13.7 | NZ_VFNW01000002.1 | 8 | circular | TerAvest/ 2019 |
| B-4492 | pZM4492 01 | 33.5 | NZ_VFOD01000003.1 | 19 | circular | TerAvest/ 2019 |
|  | pZM4492 02 | 14.9 | NZ_VFOD01000004.1 | 11 | circular | TerAvest/ 2019 |
|  | pZM4492 03 | 13.7 | NZ_VFOD01000005.1 | 11 | circular | TerAvest/ 2019 |
| DSM 12495 (CU1) | pZMCU1 01 | 25.1 | NZ_VFOH01000003.1 | 1 | linear | TerAvest/ 2019 |
|  | pZMCU1 02 | 24.7 | NZ_VFOH01000002.1 | 0 | linear | TerAvest/ 2019 |
| DSM 12497 (CU1*rif2)* | pZMCU1rif2 01 | 33.3 | NZ_VIVF01000002.1 | 53 | circular | TerAvest/ 2019 |
|  | pZMCU1rif2 02 | 32.3 | NZ_VIVF01000004.1 | 62 | circular | TerAvest/ 2019 |
|  | pZMCU1rif2 03 | 31.7 | NZ_VIVF01000003.1 | 40 | circular | TerAvest/ 2019 |
| DSM 12494 *(Uvs*51) | pZMUvs51 01 | 32.4 | NZ_VFOI01000003.1 | 58 | circular | TerAvest/ 2019 |
|  | pZMUvs51 02 | 32.3 | NZ_VFOI01000004.1 | 55 | circular | TerAvest/ 2019 |
|  | pZMUvs51 03 | 31.7 | NZ_VFOI01000005.1 | 52 | circular | TerAvest/ 2019 |
| DSM 14017 (PROIMIA1) | pZMproimiA1 01 | 35.9 | NZ_VFOG01000002.1 | 100 | circular | TerAvest/ 2019 |
|  | pZMproimiA1 02 | 29.0 | NZ_VFOG01000003.1 | 18 | circular | TerAvest/ 2019 |
|  | pZMproimiA1 03 | 20.5 | NZ_VFOG01000004.1 | 23 | circular | TerAvest/ 2019 |
| AN0101 (*francensis*) | pZMAN0101 01 | 31.9 | NZ_VFOF01000007.1 | 71 | circular | TerAvest/ 2019 |
|  | pZMAN0101 02 | 28.4 | NZ_VFOF01000006.1 | 53 | circular | TerAvest/ 2019 |
|  | pZMAN0101 03 | 27.6 | NZ_VFOF01000002.1 | 39 | circular | TerAvest/ 2019 |
|  | pZMAN0101 04 | 15.4 | NZ_VFOF01000003.1 | 7 | circular | TerAvest/ 2019 |
|  | pZMAN0101 05 | 13.7 | NZ_VFOF01000004.1 | 0 | linear | TerAvest/ 2019 |
|  | pZMAN0101 06 | 4.5 | NZ_VFOF01000005.1 | 4 | linear | TerAvest/ 2019 |

**Table S4. Loci and predicted functions for 41 genes present in ATCC10988 but without detectable homologs in any of the 3 derivative strains in the Drainas group.**

| **ATCC10988 locus** | **function** |
| --- | --- |
| ZMOB_RS00255 | hypothetical protein |
| ZMOB_RS00580 | hypothetical protein |
| ZMOB_RS00895 | hypothetical protein |
| ZMOB_RS01035 | CinA family protein |
| ZMOB_RS02115 | hypothetical protein |
| ZMOB_RS02450 | hypothetical protein |
| ZMOB_RS02480 | hypothetical protein |
| ZMOB_RS02545 | hypothetical protein |
| ZMOB_RS02895 | hypothetical protein |
| ZMOB_RS03930 | hypothetical protein |
| ZMOB_RS04130 | hypothetical protein |
| ZMOB_RS04550 | hypothetical protein |
| ZMOB_RS05250 | hypothetical protein |
| ZMOB_RS05725 | hypothetical protein |
| ZMOB_RS05895 | hypothetical protein |
| ZMOB_RS06320 | hypothetical protein |
| ZMOB_RS06340 | hypothetical protein |
| ZMOB_RS07915 | hypothetical protein |
| ZMOB_RS08000 | hypothetical protein |
| ZMOB_RS08145 | hypothetical protein |
| ZMOB_RS08415 | hypothetical protein |
| ZMOB_RS08525 | hypothetical protein |
| ZMOB_RS08815 | hypothetical protein |
| ZMOB_RS09080 | hypothetical protein |
| ZMOB_RS09220 | hypothetical protein |
| ZMOB_RS09225 | hypothetical protein |
| ZMOB_RS09340 | hypothetical protein |
| ZMOB_RS09465 | hypothetical protein |
| ZMOB_RS09620 | hypothetical protein |
| ZMOB_RS09655 | type III toxin-antitoxin system ToxN/AbiQ family toxin |
| ZMOB_RS09660 | hypothetical protein |
| ZMOB_RS09665 | hypothetical protein |
| ZMOB_RS09780 | hypothetical protein |
| ZMOB_RS09785 | hypothetical protein |
| ZMOB_RS09790 | hypothetical protein |
| ZMOB_RS09810 | hypothetical protein |
| ZMOB_RS09850 | hypothetical protein |
| ZMOB_RS09945 | membrane protein insertion efficiency factor YidD |
| ZMOB_RS09950 | 50S ribosomal protein L34 |
| ZMOB_RS09970 | hypothetical protein |
| ZMOB_RS10055 | hypothetical protein |

**Table S5. Full list of methylated sequences found in 9 de-novo sequenced *Zymomonas mobilis* genomes**.

| Sequence | Type | Strains | group | % mod |
| --- | --- | --- | --- | --- |
| gAnTc | m6A | all |  | ≥99.3 |
| cCwGg | m4C | CP1 |  | 99.6 |
| cAcnnnnnaTya | m6A | CP1 |  | 100 |
| rgAtcy | m6A | CP3 |  | 98.6 |
| craAnnnnnncTc | m6A | CP3 |  | 100 |
| rcgcAg | m6A | B-23394, B-4492 | Z6 | ≥99.9 |
| gcAnnnnnncTga | m6A | B-23394, B-4492 | Z6 | 100 |
| gcAgnnnnnnrta | m6A | CU1, CU1rif2, *uvs*51 | Drainas | 100 |
| tAynwnnnnctgc | m6A | CU1, CU1rif2, *uvs*51 | Drainas | 100 |
| bgcAnnwnnntgct | m6A | CU1, CU1rif2, *uvs*51 | Drainas | 100 |
| agcAnnnnnntgc | m6A | CU1, CU1rif2, | Drainas | 100 |
| tAynstnnnctgc | m6A | CU1rif2 |  | 100 |
| tAbanvnnnctgc | m6A | CU1rif2 |  | 56.7 |
| agcAnasnnntgc | m6A | uvs51 |  | 100 |
| agcAnnwnnntgc | m6A | uvs51 |  | 100 |
| Ccggtgncar | m4C | PROIMI A1 |  | 39.1 |
| gagntCcnntnnnnnaw | m4C | PROIMI A1 |  | 92.9 |
| cTgcAg | m6A | *francensis* |  | 100 |

**Table S6.** **Merged orthologue and annotation data.** (**see separate** **Excel file**)

**References**

Robert, X., & Gouet, P. (2014). Deciphering key features in protein structures with the new ENDscript server. *Nucleic Acids Research*, *42*(Web Server issue), W320-4. https://doi.org/10.1093/nar/gku316

Vera, J. M., Ghosh, I. N., Zhang, Y., Hebert, A. S., Coon, J. J., & Landick, R. (2020). Genome-scale transcription-translation mapping reveals features of *Zymomonas mobilis* transcription units and promoters. *MSystems*, *5*(4), e00250-20. https://doi.org/10.1128/mSystems.00250-20
