# Supplementary material for: Zymomonas diversity and potential for biofuel production
Source: Biotechnol Biofuels. 2021 May 1;14:112. doi: 10.1186/s13068-021-01958-2 (PMC8088579; doi:10.1186/s13068-021-01958-2)
Supplement: Supplementary file 2 — Additional file 2: Figure S6.Z. mobilis BcsA alignment (see figure in separate PDF). BcsA (ZMO1083) protein alignment was performed by Clustal Ω and visualized by ESPript 3.0 (Robert & Gouet, 2014). Pomaceae and ZM4 are not included as they were not annotated or annotated as pseudogene, respectively. In CP4 bcsA G629A substitution results in a stop codon (TAG); Met 42 is a start codon and protein is truncated from N-terminus. C-terminal truncation of PROIMIA1 BcsA is discussed in text. Residues with strict identity are in white on red. Threshold for high similarity was set at 0.7 and similar residues are shown in red and framed in blue. Weakly similar residues are shown in black. [file 13068_2021_1958_MOESM2_ESM.pdf]

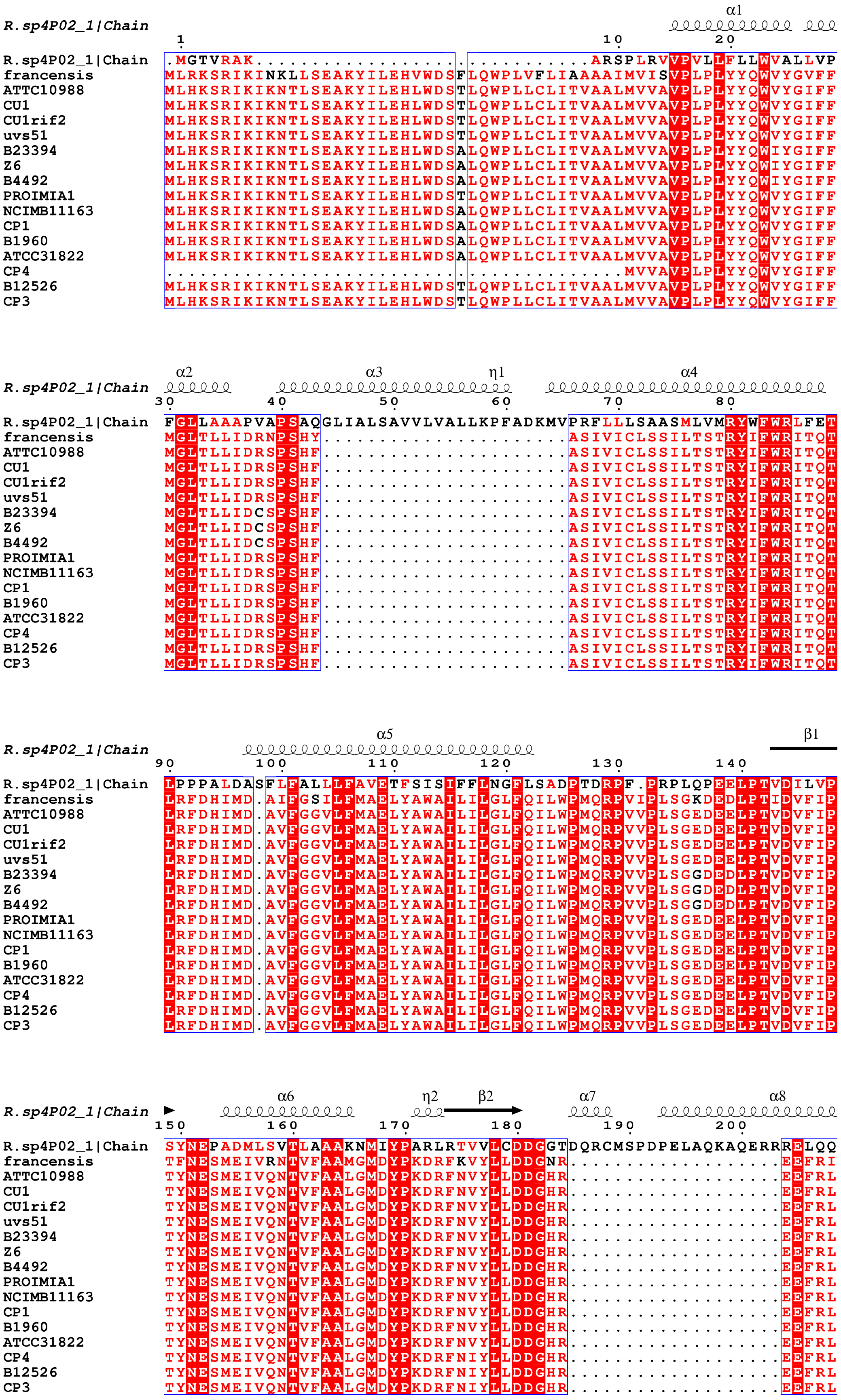
R.sp4P02_1|Chain
α1
1 10 20
R.sp4P02_1|Chain .MGTVRAK...................................ARSPLRVVPVLLFLLWVALLVP
francensis MLRKSRIKINKLLSEAKYILEHVWDSFLQWPLVFLIAAAIMVISVPLPLYYQWVYGVFF
ATTC10988 MLHKSRIKIKNTLSEAKYILEHLWDSTLQWPLLCLITVAALMVVAVPLPLYYQWVYGIFF
CU1 MLHKSRIKIKNTLSEAKYILEHLWDSTLQWPLLCLITVAALMVVAVPLPLYYQWVYGIFF
CU1rif2 MLHKSRIKIKNTLSEAKYILEHLWDSTLQWPLLCLITVAALMVVAVPLPLYYQWVYGIFF
uvs51 MLHKSRIKIKNTLSEAKYILEHLWDSTLQWPLLCLITVAALMVVAVPLPLYYQWVYGIFF
B23394 MLHKSRIKIKNTLSEAKYILEHLWDSALQWPLLCLITVAALMVVAVPLPLYYQWIYGIFF
Z6 MLHKSRIKIKNTLSEAKYILEHLWDSALQWPLLCLITVAALMVVAVPLPLYYQWIYGIFF
B4492 MLHKSRIKIKNTLSEAKYILEHLWDSALQWPLLCLITVAALMVVAVPLPLYYQWIYGIFF
PROIMIA1 MLHKSRIKIKNTLSEAKYILEHLWDSTLQWPLLCLITVAALMVVAVPLPLYYQWVYGIFF
NCIMB11163 MLHKSRIKIKNTLSEAKYILEHLWDSALQWPLLCLITVAALMVVAVPLPLYYQWVYGIFF
CP1 MLHKSRIKIKNTLSEAKYILEHLWDSALQWPLLCLITVAALMVVAVPLPLYYQWVYGIFF
B1960 MLHKSRIKIKNTLSEAKYILEHLWDSALQWPLLCLITVAALMVVAVPLPLYYQWVYGIFF
ATCC31822 MLHKSRIKIKNTLSEAKYILEHLWDSALQWPLLCLITVAALMVVAVPLPLYYQWVYGIFF
CP4 ........................................MVVAVPLPLYYQWVYGIFF
B12526 MLHKSRIKIKNTLSEAKYILEHLWDSTLQWPLLCLITVAALMVVAVPLPLYYQWVYGIFF
CP3 MLHKSRIKIKNTLSEAKYILEHLWDSTLQWPLLCLITVAALMVVAVPLPLYYQWVYGIFF
R.sp4P02_1|Chain
α2 α3 η1 α4
30 40 50 60 70 80
R.sp4P02_1|Chain FGLLAAAPVAPSAQGLIALSAVVLVALLKPFADKMVPRFLLSAASMLVMRYWFWRLFET
francensis MGLTLLIDRNPSHY.....................ASIVICLSSILTSTRYIFWRITQT
ATTC10988 MGLTLLIDRSPSHF.....................ASIVICLSSILTSTRYIFWRITQT
CU1 MGLTLLIDRSPSHF.....................ASIVICLSSILTSTRYIFWRITQT
CU1rif2 MGLTLLIDRSPSHF.....................ASIVICLSSILTSTRYIFWRITQT
uvs51 MGLTLLIDRSPSHF.....................ASIVICLSSILTSTRYIFWRITQT
B23394 MGLTLLIDCSPSHF.....................ASIVICLSSILTSTRYIFWRITQT
Z6 MGLTLLIDCSPSHF.....................ASIVICLSSILTSTRYIFWRITQT
B4492 MGLTLLIDCSPSHF.....................ASIVICLSSILTSTRYIFWRITQT
PROIMIA1 MGLTLLIDRSPSHF.....................ASIVICLSSILTSTRYIFWRITQT
NCIMB11163 MGLTLLIDRSPSHF.....................ASIVICLSSILTSTRYIFWRITQT
CP1 MGLTLLIDRSPSHF.....................ASIVICLSSILTSTRYIFWRITQT
B1960 MGLTLLIDRSPSHF.....................ASIVICLSSILTSTRYIFWRITQT
ATCC31822 MGLTLLIDRSPSHF.....................ASIVICLSSILTSTRYIFWRITQT
CP4 MGLTLLIDRSPSHF.....................ASIVICLSSILTSTRYIFWRITQT
B12526 MGLTLLIDRSPSHF.....................ASIVICLSSILTSTRYIFWRITQT
CP3 MGLTLLIDRSPSHF.....................ASIVICLSSILTSTRYIFWRITQT
R.sp4P02_1|Chain
α5 β1
90 100 110 120 130 140
R.sp4P02_1|Chain LPPPALDASFLFALLLFAVETFSISIFFLNGFLSADPTDRPF.PRPLQPEELPTVDILVP
francensis LRFDHIMD.AIFGSILFMAELYAWAILILGLFQILWPMQRPVIPLSGKDEDLPTIDVFIP
ATTC10988 LRFDHIMD.AVFGGVLFMAELYAWAILILGLFQILWPMQRPVVPLSGEDEELPTVDVFIP
CU1 LRFDHIMD.AVFGGVLFMAELYAWAILILGLFQILWPMQRPVVPLSGEDEELPTVDVFIP
CU1rif2 LRFDHIMD.AVFGGVLFMAELYAWAILILGLFQILWPMQRPVVPLSGEDEELPTVDVFIP
uvs51 LRFDHIMD.AVFGGVLFMAELYAWAILILGLFQILWPMQRPVVPLSGEDEELPTVDVFIP
B23394 LRFDHIMD.AVFGGVLFMAELYAWAILILGLFQILWPMQRPVVPLSGGDEDLPTVDVFIP
Z6 LRFDHIMD.AVFGGVLFMAELYAWAILILGLFQILWPMQRPVVPLSGGDEDLPTVDVFIP
B4492 LRFDHIMD.AVFGGVLFMAELYAWAILILGLFQILWPMQRPVVPLSGGDEDLPTVDVFIP
PROIMIA1 LRFDHIMD.AVFGGVLFMAELYAWAILILGLFQILWPMQRPVVPLSGEDEELPTVDVFIP
NCIMB11163 LRFDHIMD.AVFGGVLFMAELYAWAILILGLFQILWPMQRPVVPLSGEDEELPTVDVFIP
CP1 LRFDHIMD.AVFGGVLFMAELYAWAILILGLFQILWPMQRPVVPLSGEDEELPTVDVFIP
B1960 LRFDHIMD.AVFGGVLFMAELYAWAILILGLFQILWPMQRPVVPLSGEDEELPTVDVFIP
ATCC31822 LRFDHIMD.AVFGGVLFMAELYAWAILILGLFQILWPMQRPVVPLSGEDEELPTVDVFIP
CP4 LRFDHIMD.AVFGGVLFMAELYAWAILILGLFQILWPMQRPVVPLSGEDEELPTVDVFIP
B12526 LRFDHIMD.AVFGGVLFMAELYAWAILILGLFQILWPMQRPVVPLSGEDEELPTVDVFIP
CP3 LRFDHIMD.AVFGGVLFMAELYAWAILILGLFQILWPMQRPVVPLSGEDEELPTVDVFIP
R.sp4P02_1|Chain
α6 η2 β2 α7 α8
150 160 170 180 190 200
R.sp4P02_1|Chain SYNEPADMLSVTLAAAKNMIYPARLRTVVLCDDGGTDQRCMSPDPELAQKAQERRRELQQ
francensis TFNESMEIVRNTVFAAMGMDYPKDRFKVYLLDDGNR...................EEFRI
ATTC10988 TYNESMEIVQNTVFAALGMDYPKDRFNVYLLDDGHR...................EEFRL
CU1 TYNESMEIVQNTVFAALGMDYPKDRFNVYLLDDGHR...................EEFRL
CU1rif2 TYNESMEIVQNTVFAALGMDYPKDRFNVYLLDDGHR...................EEFRL
uvs51 TYNESMEIVQNTVFAALGMDYPKDRFNVYLLDDGHR...................EEFRL
B23394 TYNESMEIVQNTVFAALGMDYPKDRFNVYLLDDGHR...................EEFRL
Z6 TYNESMEIVQNTVFAALGMDYPKDRFNVYLLDDGHR...................EEFRL
B4492 TYNESMEIVQNTVFAALGMDYPKDRFNVYLLDDGHR...................EEFRL
PROIMIA1 TYNESMEIVQNTVFAALGMDYPKDRFNVYLLDDGHR...................EEFRL
NCIMB11163 TYNESMEIVQNTVFAALGMDYPKDRFNVYLLDDGHR...................EEFRL
CP1 TYNESMEIVQNTVFAALGMDYPKDRFNVYLLDDGHR...................EEFRL
B1960 TYNESMEIVQNTVFAALGMDYPKDRFNVYLLDDGHR...................EEFRL
ATCC31822 TYNESMEIVQNTVFAALGMDYPKDRFNVYLLDDGHR...................EEFRL
CP4 TYNESMEIVQNTVFAALGMDYPKDRFNIYLLDDGHR...................EEFRL
B12526 TYNESMEIVQNTVFAALGMDYPKDRFNIYLLDDGHR...................EEFRL
CP3 TYNESMEIVQNTVFAALGMDYPKDRFNIYLLDDGHR...................EEFRL

```
                      β3         α9          β4   TT β5     α10
R.sp4P02_1|Chain
                  210       220       230       240       250       260
R.sp4P02_1|Chain  LCRELGVVYSTRERNEHAKAGNMSAALERLKGELVVVFDADHVPSRDFLARTVGYFVEDP
francensis        FAEQAGCNYLTRTDNLNAKAGNLNAALKKTSGELVCIFDCDHVPTRAFLQLTVGWLQKEP
ATTC10988         FAEEAGCHYLTRNDNLNAKAGNLNAALKKTKGELVCIFDCDHVPTRAFLQLTVGWLQKEP
CU1               FAEEAGCHYLTRNDNLNAKAGNLNAALKKTKGELVCIFDCDHVPTRAFLQLTVGWLQKEP
CU1rif2           FAEEAGCHYLTRNDNLNAKAGNLNAALKKTKGELVCIFDCDHVPTRAFLQLTVGWLQKEP
uvs51             FAEEAGCHYLTRNDNLNAKAGNLNAALKKTKGELVCIFDCDHVPTRAFLQLTVGWLQKEP
B23394            FAEEAGCHYLTRNDNLNAKAGNLNAALKKTKGELVCIFDCDHVPTRAFLQLTVGWLQKEP
Z6                FAEEAGCHYLTRNDNLNAKAGNLNAALKKTKGELVCIFDCDHVPTRAFLQLTVGWLQKEP
B4492             FAEEAGCHYLTRNDNLNAKAGNLNAALKKTKGELVCIFDCDHVPTRAFLQLTVGWLQKEP
PROIMIA1          FAEEAGCHYLTRNDNLNAKAGNLNAALKKTKGELVCIFDCDHVPTRAFLQLTVGWLQKEP
NCIMB11163        FAEEAGCHYLTRNDNLNAKAGNLNAALRKTKGELVCIFDCDHVPTRAFLQLTVGWLQKEP
CP1               FAEEAGCHYLTRNDNLNAKAGNLNAALRKTKGELVCIFDCDHVPTRAFLQLTVGWLQKEP
B1960             FAEEAGCHYLTRNDNLNAKAGNLNAALRKTKGELVCIFDCDHVPTRAFLQLTVGWLQKEP
ATCC31822         FAEEAGCHYLTRNDNLNAKAGNLNAALKKTKGELVCIFDCDHVPTRAFLQLTVGWLQKEP
CP4               FAEEAGCHYLTRNDNLNAKAGNLNAALKKTKGELVCIFDCDHVPTRAFLQLTVGWLQKEP
B12526            FAEEAGCHYLTRNDNLNAKAGNLNAALKKTKGELVCIFDCDHVPTRAFLQLTVGWLQKEP
CP3               FAEEAGCHYLTRNDNLNAKAGNLNAALKKTKGELVCIFDCDHVPTRAFLQLTVGWLQKEP
```

```
                     β6            α11          α12    α13          β7
R.sp4P02_1|Chain
                  270       280       290       300       310       320
R.sp4P02_1|Chain  DLFLVQTPHFFINPDPIQRNLALGDRCPPENEMFYGKIHRGLDRWGGAFFCGSAAVLRRR
francensis        NLALVQTPHFFYSPDPIQKNVPGGSELPGDNELFYGTVQRGNDLWDATFFCGSCAILRRA
ATTC10988         NLALVQTPHFFYSPDPIQRNVPGGDELPGDNELFYGTVQRGNDLWDATFFCGSCAILRRE
CU1               NLALVQTPHFFYSPDPIQRNVPGGDELPGDNELFYGTVQRGNDLWDATFFCGSCAILRRE
CU1rif2           NLALVQTPHFFYSPDPIQRNVPGGDELPGDNELFYGTVQRGNDLWDATFFCGSCAILRRE
uvs51             NLALVQTPHFFYSPDPIQRNVPGGDELPGDNELFYGTVQRGNDLWDATFFCGSCAILRRE
B23394            NLALVQTPHFFYSPDPIQRNVPGGDELPGDNELFYGTVQRGNDLWDATFFCGSCAILRRE
Z6                NLALVQTPHFFYSPDPIQRNVPGGDELPGDNELFYGTVQRGNDLWDATFFCGSCAILRRE
B4492             NLALVQTPHFFYSPDPIQRNVPGGDELPGDNELFYGTVQRGNDLWDATFFCGSCAILRRE
PROIMIA1          NLALVQTPHFFYSPDPIQRNVPGGDELPGDNELFYGTVQRGNDLWDATFFCGSCAILRRE
NCIMB11163        NLALVQTPHFFYSPDPIQRNVPGGDELPGDNELFYGTVQRGNDLRDATFFCGSCAILRRE
CP1               NLALVQTPHFFYSPDPIQRNVPGGDELPGDNELFYGTVQRGNDLWDATFFCGSCAILRRE
B1960             NLALVQTPHFFYSPDPIQRNVPGGDELPGDNELFYGTVQRGNDLWDATFFCGSCAILRRE
ATCC31822         NLALVQTPHFFYSPDPIQRNVPGGDELPGDNELFYGTVQRGNDLWDATFFCGSCAILRRE
CP4               NLALVQTPHFFYSPDPIQRNVPGGDELPGDNELFYGTVQRGNDLWDATFFCGSCAILRRE
B12526            NLALVQTPHFFYSPDPIQRNVPGGDELPGDNELFYGTVQRGNDLWDATFFCGSCAILRRE
CP3               NLALVQTPHFFYSPDPIQRNVPGGDELPGDNELFYGTVQRGNDLWDATFFCGSCAILRRE
```

```
                  α14              α15            β8       β9          α16
R.sp4P02_1|Chain
                  330       340       350       360       370       380
R.sp4P02_1|Chain  ALDEAGGFAGETITEDAETALEIHSRGWKSLYIDRAMIAGLQPETFASFIQQRGRWATGM
francensis        ALEENDGFSGETVTEDAHTALRIQRRGWDTAYINIRLSAGLATDTLLAHIKQRARWARGM
ATTC10988         ALEENDGFSGETVTEDAHTALKLQRRGWDTAYINIRLSAGLATDTLLAHIKQRARWARGM
CU1               ALEENDGFSGETVTEDAHTALKLQRRGWDTAYINIRLSAGLATDTLLAHIKQRARWARGM
CU1rif2           ALEENDGFSGETVTEDAHTALKLQRRGWDTAYINIRLSAGLATDTLLAHIKQRARWARGM
uvs51             ALEENDGFSGETVTEDAHTALKLQRRGWDTAYINIRLSAGLATDTLLAHIKQRARWARGM
B23394            ALEENNGFSGETVTEDAHTALKLQRRGWDTAYINIRLSAGLATDTLLAHIKQRARWARGM
Z6                ALEENNGFSGETVTEDAHTALKLQRRGWDTAYINIRLSAGLATDTLLAHIKQRARWARGM
B4492             ALEENNGFSGETVTEDAHTALKLQRRGWDTAYINIRLSAGLATDTLLAHIKQRARWARGM
PROIMIA1          ALEENDGFSGETVTEDAHTALKLQRRGWDTAYINIRLSAGLATDTLLAHIKQRARWARGM
NCIMB11163        ALEENNGFSGETVTEDAHTALKLQRRGWDTAYINIRLSAGLATDTLLAHIKQRARWARGM
CP1               ALEENNGFSGETVTEDAHTALKLQRRGWDTAYINIRLSAGLATDTLLAHIKQRARWARGM
B1960             ALEENNGFSGETVTEDAHTALKLQRRGWDTAYINIRLSAGLATDTLLAHIKQRARWARGM
ATCC31822         ALEENNGFSGETVTEDAHTALKLQRRGWDTAYINIRLSAGLATDTLLAHIKQRARWARGM
CP4               ALEENNGFSGETVTEDAHTALKLQRRGWDTAYINIRLSAGLATDTLLAHIKQRARWARGM
B12526            ALEENNGFSGETVTEDAHTALKLQRRGWDTAYINIRLSAGLATDTLLAHIKQRARWARGM
CP3               ALEENNGFSGETVTEDAHTALKLQRRGWDTAYINIRLSAGLATDTLLAHIKQRARWARGM
```

```
                                           α17       η3         α18
R.sp4P02_1|Chain               TT TT                       *
                  390       400       410       420       430       440
R.sp4P02_1|Chain  MQMLLLKNPLFRRGLGIAQRLCYLNSMSFWFFPLVRMMFLVAPLIYLFFGIEIFVATFEE
francensis        TQILRVDNPLWGKGLTIAQRLCYMNALLHYQFALPRVIFLISPLAFILFNSSIIHSSAVM
ATTC10988         TQILRIDNPLWGRGLTIAQRLCYMNALLHYQFALPRVIFLISPLAFILFNSSIIHASAIM
CU1               TQILRIDNPLWGRGLTIAQRLCYMNALLHYQFALPRVIFLISPLAFILFNSSIIHASAIM
CU1rif2           TQILRIDNPLWGRGLTIAQRLCYMNALLHYQFALPRVIFLISPLAFILFNSSIIHASAIM
uvs51             TQILRIDNPLWGRGLTIAQRLCYMNALLHYQFALPRVIFLISPLAFILFNSSIIHASAIM
B23394            TQILRIDNPLWGRGLTIAQRLCYMNALLHYQFALPRVIFLISPLAFILFNSSIIHASAVM
Z6                TQILRIDNPLWGRGLTIAQRLCYMNALLHYQFALPRVIFLISPLAFILFNSSIIHASAVM
B4492             TQILRIDNPLWGRGLTIAQRLCYMNALLHYQFALPRVIFLISPLAFILFNSSIIHASAVM
PROIMIA1          TQILRIDNPLWGRGLTIAQRLCYMNALLHYQFALPRVIFLISPLAFILFNSSIIHASAVM
NCIMB11163        TQILRIDNPLWGRGLTIAQRLCYMNALLHYQFALPRVIFLISPLAFILFNSSIIHASAVM
CP1               TQILRIDNPLWGRGLTIAQRLCYMNALLHHQFALPRVIFLISPLAFILFNSSIIHASAVM
B1960             TQILRIDNPLWGRGLTIAQRLCYMNALLHHQFALPRVIFLISPLAFILFNSSIIHASAVM
ATCC31822         TQILRIDNPLWGRGLTIAQRLCYMNALLHYQFALPRVIFLISPLAFILFNSSIIHASAVM
CP4               TQILRIDNPLWGRGLTIAQRLCYMNALLHYQFALPRVIFLISPLAFILFNSSIIHASAVM
B12526            TQILRIDNPLWGRGLTIAQRLCYMNALLHYQFALPRVIFLISPLAFILFNSSIIHASAVM
CP3               TQILRIDNPLWGRGLTIAQRLCYMNALLHYQFALPRVIFLISPLAFILFNSSIIHASAVM
```

```
                      α19                              α20
R.sp4P02_1|Chain                                                          TTT
                  450       460       470       480       490       500

R.sp4P02_1|Chain  VLAYMPGYLAVSFLVQNALFARQRWPLVSEVYEVAQAPYLARAIVTTLLRPRSARFAVTA
francensis        VFAYAIPHLFMSLWAHERLSSGRRQPFWGEINETLLAFHLIKPTLITIFNPKKGKFNVTD
ATTC10988         VFAYVVPHLFMSLWAHERLSNGRRQPFWGEINETLLAFHLIKPTLITFLNPKKGSFNVTD
CU1               VFAYVVPHLFMSLWAHERLSNGRRQPFWGEINETLLAFHLIKPTLITFLNPKKGSFNVTD
CU1rif2           VFAYVVPHLFMSLWAHERLSNGRRQPFWGEINETLLAFHLIKPTLITFLNPKKGSFNVTD
uvs51             VFAYVVPHLFMSLWAHERLSNGRRQPFWGEINETLLAFHLIKPTLITFLNPKKGSFNVTD
B23394            VFAYAIPHLFMSLWAHERLSNGRRQPFWSEINETLLAFHLIKPTLITFFNPKKGSFNVTD
Z6                VFAYAIPHLFMSLWAHERLSNGRRQPFWSEINETLLAFHLIKPTLITFFNPKKGSFNVTD
B4492             VFAYAIPHLFMSLWAHERLSNGRRQPFWSEINETLLAFHLIKPTLITFFNPKKGSFNVTD
PROIMIA1          VFAYAIPHLFMSLWAHERLSNGRRQPFWGEINETLLAFHLIKPTLITFLNPKKGSFNVTD
NCIMB11163        VFAYAIPHLFMSLWAHERLSNGRRQPFWGEINETLLAFHLIKPTLITLSYPKKGSFNVTD
CP1               VFAYAIPHLFMSLSAHERLSNGRRQPFWGEINETLLAFHLIKPTLITFLNPKKGSFNVTD
B1960             VFAYAIPHLFMSLSAHERLSNGRRQPFWGEINETLLAFHLIKPTLITFLNPKKGSFNVTD
ATCC31822         VFAYAIPHLFMSLWAHERLSNGRRQPFWGEINETLLAFHLIKPTLITFLNPKKGSFNVTD
CP4               VFAYAIPHLFMSLWAHERLSNGRRQPFWGEINETLLAFHLIKPTLITFLNPKKGSFNVTD
B12526            VFAYAIPHLFMSLWAHERLSNGRRQPFWGEINETLLAFHLIKPTLITFLNPKKGSFNVTD
CP3               VFAYAIPHLFMSLWAHERLSNGRRQPFWGEINETLLAFHLIKPTLITFLNPKKGSFNVTD

                        β10               α21                 η4      α22
R.sp4P02_1|Chain      TT
                  510       520       530       540       550       560

R.sp4P02_1|Chain  KDETLSENYISPI.YRPLLFTFLLCLSGVLATLV...RWVAFPGDRSVLLVVGGWAVLNV
francensis        KGERSEDDYFDLRSVRPHLITTAFLFLGLVVGTIKLIYADYFHIQSSVLVLNILWASFNF
ATTC10988         KGERMEDDYFDFQSARPHIITAGFLFFGLIVGIVKLIYSSYFHIQPSVLVLNVTWASFNF
CU1               KGERMEDDYFDFQSARPHIITAGFLFFGLIVGIVKLIYSSYFHIQPSVLVLNVTWASFNF
CU1rif2           KGERMEDDYFDFQSARPHIITAGFLFFGLIVGIVKLIYSSYFHIQPSVLVLNVTWASFNF
uvs51             KGERMEDDYFDFQSARPHIITAGFLFFGLIVGIVKLIYSSYFHIQPSVLVLNVTWASFNF
B23394            KGERMEDDYFDIRSVRPHLITAGFLFLGLVVGIVKLIYSSYFHIQPSVLVLNVTWASFNF
Z6                KGERMEDDYFDIRSVRPHLITAGFLFLGLVVGIVKLIYSSYFHIQPSVLVLNVTWASFNF
B4492             KGERMEDDYFDIRSVRPHLITAGFLFLGLVVGIVKLIYSSYFHIQPSVLVLNVTWASFNF
PROIMIA1          KGERMEDDYFDIRSVRPHLITAGFLFLGLVVGIVKLIYSSYFHIQPSVLVLNVTWASFNF
NCIMB11163        KGERMEDDYFDIRSVRPHLITAGFLFLGLVVGIVKLIYSSYFHIQPSVLVLNVTWASFNF
CP1               KGERMEDDYFDIRSVRPHLIIAGFLFLGLVVGIVKLIYSSYFHIQPSVLVLNVTWASFNF
B1960             KGERMEDDYFDIRSVRPHLIIAGFLFLGLVVGIVKLIYSSYFHIQPSVLVLNVTWASFNF
ATCC31822         KGERMEDDYFDIRSVRPHLITAGFLFLGLVVGIVKLIYSSYFHIQPSVLVLNVTWASFNF
CP4               KGERMEDDYFDIRSVRPHLITAGFLFLGLVVGIVKLIYSSYFHIQPSVLVLNVTWASFNF
B12526            KGERMEDDYFDIRSVRPHLITAGFLFLGLVVGIVKLIYSSYFHIQPSVLVLNVTWASFNF
CP3               KGERMEDDYFDIRSVRPHLITAGFLFLGLVVGIVKLIYSSYFHIQPSVLVLNVTWASFNF

                          η5 β11        β12        η6       β13       β14
R.sp4P02_1|Chain
                      570       580       590       600       610       620

R.sp4P02_1|Chain  LLVGFALRAVAEKQQRRAAPRVQMEVPAEAQIPAFGNRSLTATVLDASTSGVRLLVRLPG
francensis        IILLASVAVANESRQVRNTVRFPFQIPFSAYF...DDGH....VIDSVTDN.........
ATTC10988         IILLASIAVAHESRQIRNTVRFPFRIPFTAYF...EDGH....VIDSVTDN.........
CU1               IILLASIAVAHESRQIRNTVRFPFRIPFTAYF...EDGH....VIDSVTDN.........
CU1rif2           IILLASIAVAHESRQIRNTVRFPFRIPFTAYF...EDGH....VIDSVTDN.........
uvs51             IILLASIAVAHESRQIRNTVRFPFRIPFTAYF...EDGH....VIDSVTDN.........
B23394            IILLASIAVAHESRQIRNTVRFPFRIPFTAYF...EDGH....VIDSVTDN.........
Z6                IILLASIAVAHESRQIRNTVRFPFRIPFTAYF...EDGH....VIDSVTDN.........
B4492             IILLASIAVAHESRQIRNTVRFPFRIPFTAYF...EDGH....VIDSVTDN.........
PROIMIA1          IILLASIAVAHESRQIRNTVRFPFRIPFTAYF...EDGH....VIDSVTDN.........
NCIMB11163        IILLASIAVAHESRQIRNTVRFPFRIPFTAYF...EDGH....VIDSVTDN.........
CP1               IILLASIAVAHESRQIRNTVRFPFRIPFTAYF...EDGH....VIDSVTDN.........
B1960             IILLASIAVAHESRQIRNTVRFPFRIPFTAYF...EDGH....VIDSVTDN.........
ATCC31822         IILLASIAVAHESRQIRNTVRFPFRIPFTAYF...EDGH....VIDSVTDN.........
CP4               IILLASIAVAHESRQIRNTVRFPFRIPFTAYF...EDGH....VIDSVTDN.........
B12526            IILLASIAVAHESRQIRNTVRFPFRIPFTAYF...EDGH....VIDSVTDN.........
CP3               IILLASIAVAHESRQIRNTVRFPFRIPFTAYF...EDGH....VIDSVTDN.........

                               β15          η7              β16          β17
R.sp4P02_1|Chain            TT                                       TT
                        630       640       650       660       670

R.sp4P02_1|Chain  VGDPHPALEAGGLIQFQPKKFPDAP.....QLERMVRGR.....IRSARREGGTVMVGVIF
francensis        .......ISLGGLAFSLPKNYELADRDITEISMKVDNRSFALPVKIVSRSG..ALVRLQF
ATTC10988         .......ISLGGLAFSLPKNYELADREISEISMKVDNRSFALPTKIVSKSG..SLVRLQF
CU1               .......ISLGGLAFSLPKNYELADREISEISMKVDNRSFALPTKIVSKSG..SLVRLQF
CU1rif2           .......ISLGGLAFSLPKNYELADREISEISMKVDNRSFALPTKIVSKSG..SLVRLQF
uvs51             .......ISLGGLAFSLPKNYELADREISEISMKVDNRSFALPTKIVSKSG..SLVRLQF
B23394            .......ISLGGLAFSLPKNYELADRDISEISMKVDNRSFALPTKIVSKSG..SLVRLQF
Z6                .......ISLGGLAFSLPKNYELADRDISEISMKVDNRSFALPTKIVSKSG..SLVRLQF
B4492             .......ISLGGLAFSLPKNYELADREISEISMKVDNRSFALPTKIVSKSG..SLVRLQF
PROIMIA1          .......ISLGGLAFSLPKNYELADREISEISIFF.........................
NCIMB11163        .......ISLGGLAFSLPKNYELADREISEISMKVDNRSFALPTKIVSKSG..SLVRLQF
CP1               .......ISLGGLAFSLPKNYELADREISEISMKVDNRSVALPTKIVSKSG..SLVRLQF
B1960             .......ISLGGLAFSLPKNYELADREISEISMKVDNRSVALPTKIVSKSG..SLVRLQF
ATCC31822         .......ISLGGLAFSLPKNYELADREISEISMKVDNRSFALPTKIVSKSG..SLVRLQF
CP4               .......ISLGGLAFSLPKNYELADRDIPEISMKVDNRSFALPTKIVSKSG..SLVRLQF
B12526            .......ISLGGLAFSLPKNYELADRDIPEISMKVDNRSFALPTKIVSKSG..SLVRLQF
CP3               .......ISLGGLAFSLPKNYELADRDIPEISMKVDNRSFALPTKIVSKSG..SLVRLQF
```

```
                           α23           α24                  α25
R.sp4P02_1|Chain     TT
                     680       690       700       710       720       730
R.sp4P02_1|Chain   EAGQPIAVRETVAYLIFGESAHWRTMREATMRPIGLLHGMARILWMAAASLPKTARDFMD
francensis         ETASMS.EQKKLVAALMGQADAWQHYGPE.EKPVSTIASMKHILKIIFGLLRYR......
ATTC10988          EAASMP.EQKKLVAALMGQADAWLHYGPE.EKPVSTIASMKHILKIIFGLLRYR......
CU1                EAASMP.EQKKLVAALMGQADAWLHYGPE.EKPVSTIASMKHILKIIFGLLRYR......
CU1rif2            EAASMP.EQKKLVAALMGQADAWLHYGPE.EKPVSTIASMKHILKIIFGLLRYR......
uvs51              EAASMP.EQKKLVAALMGQADAWLHYGPE.EKPVSTIASMKHILKIIFGLLRYR......
B23394             EAASMP.EQKKLVAALMGQADAWLHYGPE.EKPVSTIASMKHILKIIFGLLRYR......
Z6                 EAASMP.EQKKLVAALMGQADAWLHYGPE.EKPVSTIASMKHILKIIFGLLRYR......
B4492              EAASMP.EQKKLVAALMGQADAWLHYGPE.EKPVSTIASMKHILKIIFGLLRYR......
PROIMIA1           ............................................................
NCIMB11163         EAASMP.EQKKLVAALMGQADAWLHYGPE.EKPVSTIASMKHILKIIFGLLRYR......
CP1                EAASMP.EQKKLVAALMGQADAWLHYGPE.EKPVSTIASMKHILKIIFGLLRYR......
B1960              EAASMP.EQKKLVAALMGQADAWLHYGPE.EKPVSTIASMKHILKIIFGLLRYR......
ATCC31822          EAASMP.EQKKLVAALMGQADAWLHYGPE.EKPVSTIASMKHILKIIFGLLRYR......
CP4                EAASMP.EQKKLVAALMGQADAWLHYGPE.EKPVSTIASMKHILKIIFGLLRYR......
B12526             EAASMP.EQKKLVAALMGQADAWLHYGPE.EKPVSTIASMKHILKIIFGLLRYR......
CP3                EAASMP.EQKKLVAALMGQADAWLHYGPE.EKPVSTIASMKHILKIIFGLLRYR......

R.sp4P02_1|Chain
                         740       750       760       770       780       790
R.sp4P02_1|Chain   EPARRRRRHEEPKEKQAHLLAFGTDFSTEPDWAGELLDPTAQVSARPNTVAWGSNHHHHH
francensis         .PQKRINKKAAIKNEKGAVQK.......................................
ATTC10988          .PQKRINKKAAIKNEKGAVKK.......................................
CU1                .PQKRINKKAAIKNEKGAVKK.......................................
CU1rif2            .PQKRINKKAAIKNEKGAVKK.......................................
uvs51              .PQKRINKKAAIKNEKGAVKK.......................................
B23394             .PQKRINKKAAIKNEKGAVKK.......................................
Z6                 .PQKRINKKAAIKNEKGAVKK.......................................
B4492              .PQKRINKKAAIKNEKGAVKK.......................................
PROIMIA1           ............................................................
NCIMB11163         .PQKRINKKAAIKNEKGAVKK.......................................
CP1                .PQKRINKKAAIKNEKGAVKK.......................................
B1960              .PQKRINKKAAIKNEKGAVKK.......................................
ATCC31822          .PQKRINKKAAIKNEKGAVKK.......................................
CP4                .PQKRINKKAAIKNEKGAVKK.......................................
B12526             .PQKRINKKAAIKNEKGAVKK.......................................
CP3                .PQKRINKKAAIKNEKGAVKK.......................................

R.sp4P02_1|Chain
                         800
R.sp4P02_1|Chain   HKLHHHHHH
francensis         .........
ATTC10988          .........
CU1                .........
CU1rif2            .........
uvs51              .........
B23394             .........
Z6                 .........
B4492              .........
PROIMIA1           .........
NCIMB11163         .........
CP1                .........
B1960              .........
ATCC31822          .........
CP4                .........
B12526             .........
CP3                .........
```
