# Supplementary material for: Zymomonas diversity and potential for biofuel production
Source: Biotechnol Biofuels. 2021 May 1;14:112. doi: 10.1186/s13068-021-01958-2 (PMC8088579; doi:10.1186/s13068-021-01958-2)
Supplement: Supplementary file 3 — Additional file 3: Figure S7. Z. mobilis BcsA alignment with secondary structures from Rhodobacter sphaeroides 4P02_A (see figure in separate PDF). BcsA (ZMO1083) was aligned with BcsA from R. sphaeroides using Clustal Ω and visualized by ESPript 3.0 (1). Secondary structures derived from R. sp. 3D structure (PDB 4P02) are shown at the top. α and π helices are shown in medium and small squiggles, β sheets as arrows and α or β turns as TT or TTT, respectively. Color code is as in Figure S5. β-16 and β-17 of R. sphaeroids are part of a PilZ domain binding c-di GMP. [file 13068_2021_1958_MOESM3_ESM.pdf]

```
             1        10        20        30        40        50        60
ZM4          MKRIKGITLASSLAGAMMLGSLAVDPLPASVSKNPTPDNKTGATQTGTTAGSGSAIQLLR
B23394       MKRIKGITLASSLAGAMMLGSLAVDPLPASVSKNPTPDNKTGATQTGTTAGSGSAIQLLR
Z6           MKRIKGITLASSLAGAMMLGSLAVDPLPASVSKNPTPDNKTGATQTGTTAGSGSAIQLLR
B4492        MKRIKGITLASSLAGAMMLGSLAVDPLPASVSKNPTPDNKTGATQTGTTAGSGSAIQLLR
ATCC10988    MKRITGITLASSLAGAMMLGSLAVDPLPASVSKNPTPDNKTGATQTGATAGSGSAIQLLR
CU1          MKRITGITLASSLAGAMMLGSLAVDPLPASVSKNPTPDNKTGATQTGATAGSGSAIQLLR
CU1rif2      MKRITGITLASSLAGAMMLGSLAVDPLPASVSKNPTPDNKTGATQTGATAGSGSAIQLLR
uvs51        MKRITGITLASSLAGAMMLGSLAVDPLPASVSKNPTPDNKTGATQTGATAGSGSAIQLLR
NCIMB11163   MKRITGITLASSLAGAMMLGSLAVDPLPASVSKNPTPDNKTGATQTGATAGSGSAIQLLR
PROIMIA1     MKRITGITLASSLAGAMMLGSLAVDPLPASVSKNPTPDNKTGATQTGATAGSGSAIQLLR
CP3          MKRITGITLASSLAGAMMLGSLAVDPLPASVSKNPTPDNKTGATQTGATAGSGSAIQLLR
B12526       MKRITGITLASSLAGAMMLGSLAVDPLPASVSKNPTPDNKTGATQTGATAGSGSAIQLLR
CP4          MKRITGITLASSLAGAMMLGSLAVDPLPASVSKNPTPDNKTGATQTGATAGSGSAIQLLR
CP1          MKRITGITLASSLAGAMMLGSLAVDPLPASVSKNPTPDNKTGATQTGATAGSGSAIQLLR
B1960        MKRITGITLASSLAGAMMLGSLAVDPLPASVSKNPTPDNKTGATQTGATAGSGSAIQLLR
ATCC31822    MKRITGITLASSLAGAMMLGSLAVDPLPASVSKNPTPDNKTGATQTGATAGSGSAIQLLR
```

```
                      70        80        90       100       110       120
ZM4          HQAIFWKNRGRNDLAANAWQRLLEIDPNNKMAKQALQESTFVPAAPESDSSPNNAPTKIV
B23394       HQAIFWKNRGRNDLAANAWQRLLEIDPNNKMAKQALQESTFVPAAPESDSSPNNAPTKIV
Z6           HQAIFWKNRGRNDLAANAWQRLLEIDPNNKMAKQALQESTFVPAAPESDSSPNNAPTKIV
B4492        HQAIFWKNRGRNDLAANAWQRLLEIDPNNKMAKQALQESTFVPAAPESDSSPNNAPTKIV
ATCC10988    HQAIFWKNRGRNDLAANAWQRLLEIDPNNKMAKQALQESTFVPAAPESDNSPNNAPTKIV
CU1          HQAIFWKNRGRNDLAANAWQRLLEIDPNNKMAKQALQESTFVPAAPESDNSPNNAPTKIV
CU1rif2      HQAIFWKNRGRNDLAANAWQRLLEIDPNNKMAKQALQESTFVPAAPESDNSPNNAPTKIV
uvs51        HQAIFWKNRGRNDLAANAWQRLLEIDPNNKMAKQALQESTFVPAAPESDNSPNNAPTKIV
NCIMB11163   HQAIFWKNRGRNDLAANAWQRLLEIDPNNKMAKQALQESTFVPAAPESDSSPNNAPTKIV
PROIMIA1     HQAIFWKNRGRNDLAANAWQRLLEIDPNNKMAKQALQESTFVPAAPESDSSPNNAPTKIV
CP3          HQAIFWKNRGRNDLAANAWQRLLEIDPNNKMAKQALQESTFVPAAPESDSSPNNAPTKIV
B12526       HQAIFWKNRGRNDLAANAWQRLLEIDPNNKMAKQALQESTFVPAAPESDSSPNNAPTKIV
CP4          HQAIFWKNRGRNDLAANAWQRLLEIDPNNKMAKQALQESTFVPAAPESDSSPNNAPTKIV
CP1          HQAIFWKNRGRNDLAANAWQRLLEIDPNNKMAKQALQESTFVPAAPESDSSPNNAPTKIV
B1960        HQAIFWKNRGRNDLAANAWQRLLEIDPNNKMAKQALQESTFVPAAPESDSSPNNAPTKIV
ATCC31822    HQAIFWKNRGRNDLAANAWQRLLEIDPNNKMAKQALQESTFVPAAPESDSSPNNAPTKIV
```

```
                     130       140       150       160       170       180
ZM4          TGQGGRSFPTGLTPSARAGRIRLEGFNAFKNNQLDIAEQRFRRALELYAHDKDALGGLGI
B23394       TGQGGRSFPTGLTPSARAGRIRLEGFNAFKNNQLDIAEQRFRRALELYAHDKDALGGLGI
Z6           TGQGGRSFPTGLTPSARAGRIRLEGFNAFKNNQLDIAEQRFRRALELYAHDKDALGGLGI
B4492        TGQGGRSFPTGLTPSARAGRIRLEGFNAFKNNQLDIAEQRFRRALELYAHDKDALGGLGI
ATCC10988    TGQGGRSFPTGLTPSARAGCIRLEGFNAFKNNQLDIAEQRFRRALALYAHDKDALGGLGI
CU1          TGQGGRSFPTGLTPSARAGCIRLEGFNAFKNNQLDIAEQRFRRALALYAHDKDALGGLGI
CU1rif2      TGQGGRSFPTGLTPSARAGCIRLEGFNAFKNNQLDIAEQRFRRALALYAHDKDALGGLGI
uvs51        TGQGGRSFPTGLTPSARAGCIRLEGFNAFKNNQLDIAEQRFRRALALYAHDKDALGGLGI
NCIMB11163   TGQGGRSFPTGLTPSARAGRIRLEGFNAFKNNQLDIAEQRFRRALALYAHDKDALGGLGI
PROIMIA1     TGQGGRSFPTGLTPSARAGRIRLEGFNAFKNNQLDIAEQRFRRALALYAHDKDALGGLGI
CP3          TGQGGRSFPTGLTPSARAGRIRLEGFNAFKNNQLDIAEQRFRRALALYAHDKDALGGLGI
B12526       TGQGGRSFPTGLTPSARAGRIRLEGFNAFKNNQLDIAEQRFRRALALYAHDKDALGGLGI
CP4          TGQGGRSFPTGLTPSARAGRIRLEGFNAFKNNQLDIAEQRFRRALALYAHDKDALGGLGI
CP1          TGQGGRSFPTGLTPSARAGRIRLEGFNAFKNNQLDIAEQRFRRALALYAHDKDALGGLGI
B1960        TGQGGRSFPTGLTPSARAGRIRLEGFNAFKNNQLDIAEQRFRRALALYAHDKDALGGLGI
ATCC31822    TGQGGRSFPTGLTPSARAGRIRLEGFNAFKNNQLDIAEQRFRRALALYAHDKDALGGLGI
```

```
                     190       200       210       220       230       240
ZM4          IRLKQHRYAEAIRYLEEASSDKDGYRWANALSSARFYGRFEEAQAAIRANQLDKAQSILE
B23394       IRLKQHRYAEAIRYLEEASSDKDGYRWANALSSARFYGRFEEAQAAIRANQLDKAQSILE
Z6           IRLKQHRYAEAIRYLEEASSDKDGYRWANALSSARFYGRFEEAQAAIRANQLDKAQSILE
B4492        IRLKQHRYAEAIRYLEEASSDKDGYRWANALSSARFYGRFEEAQAAIRANQLDKAQSILE
ATCC10988    IRLKQHRYAEAIRYLEEASSDKEGYHWANALSSARFYGRFEEAQAASRANQLDKAQSILE
CU1          IRLKQHRYAEAIRYLEEASSDKEGYHWANALSSARFYGRFEEAQAASRANQLDKAQSILE
CU1rif2      IRLKQHRYAEAIRYLEEASSDKEGYHWANALSSARFYGRFEEAQAASRANQLDKAQSILE
uvs51        IRLKQHRYAEAIRYLEEASSDKEGYHWANALSSARFYGRFEEAQAASRANQLDKAQSILE
NCIMB11163   IRLKQHRYAEAIRYLEEASSDKEGYRWANALSSARFYGRFEEAQAASRANQLDKAQSILE
PROIMIA1     IRLKQHRYAEAIRYLEEASSDKEGYRWANALSSARFYGRFEEAQAASRANQLDKAQSILE
CP3          IRLKQHRYAEAIRYLEEASSDKEGYRWANALSSARFYGRFEEAQAASRANQLDKAQSILE
B12526       IRLKQHRYAEAIRYLEEASSDKEGYRWANALSSARFYGRFEEAQAASRANQLDKAQSILE
CP4          IRLKQHRYAEAIRYLEEASSDKEGYRWANALSSARFYGRFEEAQAASRANQLDKAQSILE
CP1          IRLKQHRYAEAIRYLEEASSDKEGYRWANALSSARFYGRFEEAQAASRANQLDKAQSILE
B1960        IRLKQHRYAEAIRYLEEASSDKEGYRWANALSSARFYGRFEEAQAASRANQLDKAQSILE
ATCC31822    IRLKQHRYAEAIRYLEEASSDKEGYRWANALSSARFYGRFEEAQAASRANQLDKAQSILE
```

```
                  250       260       270       280       290       300
ZM4          QLQNQQGGDANVSNALLADIYARQGQFDKAMALYDKLGNSADAVTVARLRSQENSIKARR
B23394       QLQNQQGGDANVSNALLADIYARQGQFDKAMALYDKLGNSADAVTVARLRSQENSIKARR
Z6           QLQNQQGGDANVSNALLADIYARQGQFDKAMALYDKLGNSADAVTVARLRSQENSIKARR
B4492        QLQNQQGGDANVSNALLADIYARQGQFDKAMALYDKLGNSADAVTVARLRSQENSIKARR
ATCC10988    QLQNQQGGDANVSNALLADIYARQGQFDKAMALYDKLGNSADAVTVARLRSQENSIKARR
CU1          QLQNQQGGDANVSNALLADIYARQGQFDKAMALYDKLGNSADAVTVARLRSQENSIKARR
CU1rif2      QLQNQQGGDANVSNALLADIYARQGQFDKAMALYDKLGNSADAVTVARLRSQENSIKARR
uvs51        QLQNQQGGDANVSNALLADIYARQGQFDKAMALYDKLGNSADAVTVARLRSQENSIKARR
NCIMB11163   QLQNQQGGDANVSNALLADIYARQGQFDKAMALYDKLGNSADAVTVARLRSQENSIKARR
PROIMIA1     QLQNQQGGDANVSNALLADIYARQGQFDKAMALYDKLGNSADAVTVARLRSQENSIKARR
CP3          QLQNQQGGDANVSNALLADIYARQGQFDKAMALYDKLGNSADAVTVARLRSQENSIKARR
B12526       QLQNQQGGDANVSNALLADIYARQGQFDKAMALYDKLGNSADAVTVARLRSQENSIKARR
CP4          QLQNQQGGDANVSNALLADIYARQGQFDKAMALYDKLGNSADAVTVARLRSQENSIKARR
CP1          QLQNQQGGDANVSNALLADIYARQGQFDKAMALYDKLGNSADAVTVARLRSQENSIKARR
B1960        QLQNQQGGDANVSNALLADIYARQGQFDKAMALYDKLGNSADAVTVARLRSQENSIKARR
ATCC31822    QLQNQQGGDANVSNALLADIYARQGQFDKAMALYDKLGNSADAVTVARLRSQENSIKARR

                  310       320       330       340       350       360
ZM4          ALSQGDYAHAEQYFRTAIANDIANPWLRLDYARLLLKEGHRPEAVSLIRPLEDQASSEEA
B23394       ALSQGDYAHAEQYFRTAIANDIANPWLRLDYARLLLKEGHRPEAVSLIRPLEDQASSEEA
Z6           ALSQGDYAHAEQYFRTAIANDIANPWLRLDYARLLLKEGHRPEAVSLIRPLEDQASSEEA
B4492        ALSQGDYAHAEQYFRTAIANDIANPWLRLDYARLLLKEGHRPEAVSLIRPLEDQASSEEA
ATCC10988    ALSQGDYAHAEQYFRTAIANDIANPWLRLDYARLLLKEGHRPEAVSLIRPLEDQASSEEA
CU1          ALSQGDYAHAEQYFRTAIANDIANPWLRLDYARLLLKEGHRPEAVSLIRPLEDQASSEEA
CU1rif2      ALSQGDYAHAEQYFRTAIANDIANPWLRLDYARLLLKEGHRPEAVSLIRPLEDQASSEEA
uvs51        ALSQGDYAHAEQYFRTAIANDIANPWLRLDYARLLLKEGHRPEAVSLIRPLEDQASSEEA
NCIMB11163   ALSQGDYAHAEQYFRTAIANDIANPWLRLDYARLLLKEGHRPEAVSLIRPLEDQASSEEA
PROIMIA1     ALSQGDYAHAEQYFRTAIANDIANPWLRLDYARLLLKEGHRPEAVSLIRPLEDQASSEEA
CP3          ALSQGDYAHAEQYFRTAIANDIANPWLRLDYARLLLKEGHRPEAVSLIRPLEDQASSEEA
B12526       ALSQGDYAHAEQYFRTAIANDIANPWLRLDYARLLLKEGHRPEAVSLIRPLEDQASSEEA
CP4          ALSQGDYAHAEQYFRTAIANDIANPWLRLDYARLLLKEGHRPEAVSLIRPLEDQASSEEA
CP1          ALSQGDYAHAEQYFRTAIANDIANPWLRLDYARLLLKEGHRPEAVSLIRPLEDQASSEEA
B1960        ALSQGDYAHAEQYFRTAIANDIANPWLRLDYARLLLKEGHRPEAVSLIRPLEDQASSEEA
ATCC31822    ALSQGDYAHAEQYFRTAIANDIANPWLRLDYARLLLKEGHRPEAVSLIRPLEDQASSEEA

                  370       380       390       400       410       420
ZM4          IYAASLFWQEMGDNRHVISLIERIPDDARTPLINQIAESADINIALARAKRMSLYGRRGD
B23394       IYAASLFWQEMGDNRHVISLIERIPDDARTPLINQIAESADINIALARAKRMSLYGRRGD
Z6           IYAASLFWQEMGDNRHVISLIERIPDDARTPLINQIAESADINIALARAKRMSLYGRRGD
B4492        IYAASLFWQEMGDNRHVISLIERIPDDARTPLINQIAESADINIALARAKRMSLYGRRGD
ATCC10988    IYAASLFWQEMGDNRHVISLIERIPDDARTPLINQIAESADINIALARAKRMSLYGRRGD
CU1          IYAASLFWQEMGDNRHVISLIERIPDDARTPLINQIAESADINIALARAKRMSLYGRRGD
CU1rif2      IYAASLFWQEMGDNRHVISLIERIPDDARTPLINQIAESADINIALARAKRMSLYGRRGD
uvs51        IYAASLFWQEMGDNRHVISLIERIPDDARTPLINQIAESADINIALARAKRMSLYGRRGD
NCIMB11163   IYAASLFWQEMGDNRHVISLIERIPDDARTPLINQIAESADINIALARAKRMSLYGRRGD
PROIMIA1     IYAASLFWQEMGDNRHVISLIERIPDDARTPLINQIAESADINIALARAKRMSLYGRRGD
CP3          IYAASLFWQEMGDNRHVISLIERIPDDARTPLINQIAESADINIALARAKRMSLYGRRGD
B12526       IYAASLFWQEMGDNRHVISLIERIPDDARTPLINQIAESADINIALARAKRMSLYGRRGD
CP4          IYAASLFWQEMGDNRHVISLIERIPDDARTPLINQIAESADINIALARAKRMSLYGRRGD
CP1          IYAASLFWQEMGDNRHVISLIERIPDDARTPLINQIAESADINIALARAKRMSLYGRRGD
B1960        IYAASLFWQEMGDNRHVISLIERIPDDARTPLINQIAESADINIALARAKRMSLYGRRGD
ATCC31822    IYAASLFWQEMGDNRHVISLIERIPDDARTPLINQIAESADINIALARAKRMSLYGRRGD

                  430       440       450       460       470       480
ZM4          AIGLLRSLANDPNRSISQLGMIADGLLQLGDVEDAAFLAEHSLSLPLGEANDYQASLSVL
B23394       AIGLLRSLANDPNRSISQLGMIADGLLQLGDVEDAAFLAEHSLSLPLGEANDYQASLSVL
Z6           AIGLLRSLANDPNRSISQLGMIADGLLQLGDVEDAAFLAEHSLSLPLGEANDYQASLSVL
B4492        AIGLLRSLANDPNRSISQLGMIADGLLQLGDVEDAAFLAEHSLSLPLGEANDYQASLSVL
ATCC10988    AIGLLRSLANDPNRSIPQLGMIADGLLQLGDVEDAAFLAEHSLSLPLGEANDYQASLSVL
CU1          AIGLLRSLANDPNRSIPQLGMIADGLLQLGDVEDAAFLAEHSLSLPLGEANDYQASLSVL
CU1rif2      AIGLLRSLANDPNRSIPQLGMIADGLLQLGDVEDAAFLAEHSLSLPLGEANDYQASLSVL
uvs51        AIGLLRSLANDPNRSIPQLGMIADGLLQLGDVEDAAFLAEHSLSLPLGEANDYQASLSVL
NCIMB11163   AIGLLRSLANDPNRSISQLGMIADGLLQLGDVEDAAFLAEHSLSLPLGEANDYQASLSVL
PROIMIA1     AIGLLRSLANDPNRSISQLGMIADGLLQLGDVEDAAFLAEHSLSLPLGEANDYQASLSVL
CP3          AIGLLRSLANDPNRSISQLGMIADGLLQLGDVEDAAFLAEHSLSLPLGEANDYQASLSVL
B12526       AIGLLRSLANDPNRSISQLGMIADGLLQLGDVEDAAFLAEHSLSLPLGEANDYQASLSVL
CP4          AIGLLRSLANDPNRSISQLGMIADGLLQLGDVEDAAFLAEHSLSLPLGEANDYQASLSVL
CP1          AIGLLRSLANDPNRSISQLGMIADGLLQLGDVEDAAFLAEHSLSLPLGEANDYQASLSVL
B1960        AIGLLRSLANDPNRSISQLGMIADGLLQLGDVEDAAFLAEHSLSLPLGEANDYQASLSVL
ATCC31822    AIGLLRSLANDPNRSISQLGMIADGLLQLGDVEDAAFLAEHSLSLPLGEANDYQASLSVL
```

```
                     490       500       510       520       530       540
ZM4          IRTGHYDTASDFISQIASEMSRHDQNGYKMLNRTLAIARADMMRQKKLYMPAFDVLHQAW
B23394       IRTGHYDTASDFISQIASEMSRHDQNGYKMLNRTLAIARADMMRQKKLYMPAFDVLHQAW
Z6           IRTGHYDTASDFISQIASEMSRHDQNGYKMLNRTLAIARADMMRQKKLYMPAFDVLHQAW
B4492        IRTGHYDTASDFISQIASEMSRHDQNGYKMLNRTLAIARADMMRQKKLYMPAFDVLHQAW
ATCC10988    IRTGHYDTASDFISQIASEMSRHDQNGYKMLNRTLAIARADMMRQKKLYMPAFDVLHQAW
CU1          IRTGHYDTASDFISQIASEMSRHDQNGYKMLNRTLAIARADMMRQKKLYMPAFDVLHQAW
CU1rif2      IRTGHYDTASDFISQIASEMSRHDQNGYKMLNRTLAIARADMMRQKKLYMPAFDVLHQAW
uvs51        IRTGHYDTASDFISQIASEMSRHDQNGYKMLNRTLAIARADMMRQKKLYMPAFDVLHQAW
NCIMB11163   IRTGHYDTASDFISQIASEMSRHDQNGYKMLNRTLAIARADMMRQKKLYMPAFDVLHQAW
PROIMIA1     IRTGHYDTASDFISQIASEMSRHDQNGYKMLNRTLAIARADMMRQKKLYMPAFDVLHQAW
CP3          IRTGHYDTASDFISQIASEMSRHDQNGYKMLNRTLAIARADMMRQKKLYMPAFDVLHQAW
B12526       IRTGHYDTASDFISQIASEMSRHDQNGYKMLNRTLAIARADMMRQKKLYMPAFDVLHQAW
CP4          IRTGHYDTASDFISQIASEMSRHDQNGYKMLNRTLAIARADMMRQKKLYMPAFDVLHQAW
CP1          IRTGHYDTASDFISQIASEMSRHDQNGYKMLNRTLAIARADMMRQKKLYMPAFDVLHQAW
B1960        IRTGHYDTASDFISQIASEMSRHDQNGYKMLNRTLAIARADMMRQKKLYMPAFDVLHQAW
ATCC31822    IRTGHYDTASDFISQIASEMSRHDQNGYKMLNRTLAIARADMMRQKKLYMPAFDVLHQAW
```

```
                     550       560       570       580       590       600
ZM4          EETGGGDNILPVLARIYQDAGLTDKAQSLYHYMLRNHPRDASALLNSLTIAQQQGNDDRA
B23394       EETGGGDNILPVLARIYQDAGLTDKAQSLYHYMLRNHPRDASALLNSLTIAQQQGNDDRA
Z6           EETGGGDNILPVLARIYQDAGLTDKAQSLYHYMLRNHPRDASALLNSLTIAQQQGNDDRA
B4492        EETGGGDNILPVLARIYQDAGLTDKAQSLYHYMLRNHPRDASALLNSLTIAQQQGNDDRA
ATCC10988    EETGGGDNILPVLARIYQDAGLTDKAQSLYHYMLRNHPRDASALLNSLTIAQQQGNDDRA
CU1          EETGGGDNILPVLARIYQDAGLTDKAQSLYHYMLRNHPRDASALLNSLTIAQQQGNDDRA
CU1rif2      EETGGGDNILPVLARIYQDAGLTDKAQSLYHYMLRNHPRDASALLNSLTIAQQQGNDDRA
uvs51        EETGGGDNILPVLARIYQDAGLTDKAQSLYHYMLRNHPRDASALLNSLTIAQQQGNDDRA
NCIMB11163   EETGGGDNILPVLARIYQDAGLTDKAQSLYHYMLRNHPRDASALLNSLTIAQQQGNDDRA
PROIMIA1     EETGGGDNILPVLARIYQDAGLTDKAQSLYHYMLRNHPRDASALLNSLTIAQQQGNDDRA
CP3          EETGGGDNILPVLARIYQDAGLTDKAQSLYHYMLRNHPRDASALLNSLTIAQQQGNDDRA
B12526       EETGGGDNILPVLARIYQDAGLTDKAQSLYHYMLRNHPRDASALLNSLTIAQQQGNDDRA
CP4          EETGGGDNILPVLARIYQDAGLTDKAQSLYHYMLRNHPRDASALLNSLTIAQQQGNDDRA
CP1          EETGGGDNILPVLAKIYQDAGLTDKAQSLYHYMLRNHPRDASALLNSLTIAQQQGNDDRA
B1960        EETGGGDNILPVLAKIYQDAGLTDKAQSLYHYMLRNHPRDASALLNSLTIAQQQGNDDRA
ATCC31822    EETGGGDNILPVLARIYQDAGLTDKAQSLYHYMLRNHPRDASALLNSLTIAQQQGNDDRA
```

```
                     610       620       630       640       650       660
ZM4          EQALRRLKKVAPSNPYVYLASARFEKSKSDDEKALKDLKKAHRVYQRMVRQGRSADLPSD
B23394       EQALRRLKKVAPSNPYVYLASARFEKSKSDDEKALKDLKKAHRVYQRMVRQGRSADLPSD
Z6           EQALRRLKKVAPSNPYVYLASARFEKSKSDDEKALKDLKKAHRVYQRMVRQGRSADLPSD
B4492        EQALRRLKKVAPSNPYVYLASARFEKSKSDDEKALKDLKKAHRVYQRMVRQGRSADLPSD
ATCC10988    EQALRRLKKVAPGNPYVYLASARFEKSKSDDEQALKDLKKAHRVYQRMVRQGRSADLPSD
CU1          EQALRRLKKVAPGNPYVYLASARFEKSKSDDEQALKDLKKAHRVYQRMVRQGRSADLPSD
CU1rif2      EQALRRLKKVAPGNPYVYLASARFEKSKSDDEQALKDLKKAHRVYQRMVRQGRSADLPSD
uvs51        EQALRRLKKVAPGNPYVYLASARFEKSKSDDEQALKDLKKAHRVYQRMVRQGRSADLPSD
NCIMB11163   EQALRRLKKVAPGNPYVYLASARFEKSKSDDEQALKDLKKAHRVYQRMVRQGRSADLPSD
PROIMIA1     EQALRRLKKVAPGNPYVYLASARFEKSKSDDEQALKDLKKAHRVYQRMVRQGRSADLPSD
CP3          EQALRRLKKVAPGNPYVYLASARFEKSKSDDEQALKDLKKAHRVYQRMVRQGRSADLPSD
B12526       EQALRRLKKVAPGNPYVYLASARFEKSKSDDEQALKDLKKAHRVYQRMVRQGRSADLPSD
CP4          EQALRRLKKVAPGNPYVYLASARFEKSKSDDEQALKDLKKAHRVYQRMVRQGRSADLPSD
CP1          EQALRRLKKVAPGNPYVYLASARFEKSKSDDEQALKDLKKAHRVYQRMVRQGRSADLPSD
B1960        EQALRRLKKVAPGNPYVYLASARFEKSKSDDEQALKDLKKAHRVYQRMVRQGRSADLPSD
ATCC31822    EQALRRLKKVAPGNPYVYLASARFEKSKSDDEQALKDLKKAHRVYQRMVRQGRSADLPSD
```

```
                     670       680       690       700       710       720
ZM4          AANDDNSSIVVDAPNLLRDNYHPFALNEEDFRRLQTRSQVEMASSDSDADMDRDDYHYNR
B23394       AANDDNSSIVVDAPNLLRDNYHPFALNEEDFRRLQTRSQVEMASSDSDADMDRDDYHYNR
Z6           AANDDNSSIVVDAPNLLRDNYHPFALNEEDFRRLQTRSQVEMASSDSDADMDRDDYHYNR
B4492        AANDDNSSIVVDAPNLLRDNYHPFALNEEDFRRLQTRSQVEMASSDSDADMDRDDYHYNR
ATCC10988    AANDDNSPIVVDAPNLPRDNYNPFALNEEDFRRLQTRSQVEMASSDSDADIDRDDYHYNR
CU1          AANDDNSPIVVDAPNLPRDNYNPFALNEEDFRRLQTRSQVEMASSDSDADIDRDDYHYNR
CU1rif2      AANDDNSPIVVDAPNLPRDNYNPFALNEEDFRRLQTRSQVEMASSDSDADIDRDDYHYNR
uvs51        AANDDNSPIVVDAPNLPRDNYNPFALNEEDFRRLQTRSQVEMASSDSDADIDRDDYHYNR
NCIMB11163   VANDDNSPIVVDAPNLPRDNYNPFALNEEDFRRLQTRSQVEMASSDSDADIDRDDYHYNR
PROIMIA1     AANDDNSPIVVDAPNLPRDNYNPFALNEEDFRRLQTRSQVEMASSDSDADIDRDDYHYNR
CP3          AANDDNSPIVVDAPNLPRDNYNPFALNEEDFRRLQTRSQVEMASSDSDADIDRDDYHYNR
B12526       AANDDNSPIVVDAPNLPRDNYNPFALNEEDFRRLQTRSQVEMASSDSDADIDRDDYHYNR
CP4          AANDDNSPIVVDAPNLPRDNYNPFALNEEDFRRLQTRSQVEMASSDSDADIDRDDYHYNR
CP1          AANDDNSPIVVDAPNLPRDNYNPFALNEEDFRRLQTRSQVEMASSDSDADIDRDDYHYNR
B1960        AANDDNSPIVVDAPNLPRDNYNPFALNEEDFRRLQTRSQVEMASSDSDADIDRDDYHYNR
ATCC31822    AANDDNSPIVVDAPNLPRDNYNPFALNEEDFRRLQTRSQVEMASSDSDADIDRDDYHYNR
```

```
                   730       740       750       760       770       780
ZM4          DDYDDGFGQTSSYHSARSSGGNNDRFANGTSYQKSQQSPVKQPSGFSSHPLFASSDEENF
B23394       DDYDDGFGQTSSYHSARSSGGNNDRFANGTSYQKSQQSPVKQPSGFSSHPLFASSDEENF
Z6           DDYDDGFGQTSSYHSARSSGGNNDRFANGTSYQKSQQSPVKQPSGFSSHPLFASSDEENF
B4492        DDYDDGFGQTSSYHSARSSGGNNDRFANGTSYQKSQQSPVKQPSGFSSHPLFASSDEENF
ATCC10988    DDYDDGFGQTSSYHSARSSGGNNDRFANGTSYQKPQQPAVKQPSGFSSHPLFASSDEENF
CU1          DDYDDGFGQTSSYHSARSSGGNNDRFANGTSYQKPQQPAVKQPSGFSSHPLFASSDEENF
CU1rif2      DDYDDGFGQTSSYHSARSSGGNNDRFANGTSYQKPQQPAVKQPSGFSSHPLFASSDEENF
uvs51        DDYDDGFGQTSSYHSARSSGGNNDRFANGTSYQKPQQPAVKQPSGFSSHPLFASSDEENF
NCIMB11163   DDYDDGFGQTSSYHSARSSGGNNDRFANGTSYQKPQQSAVKQPSGFSSHPLFASSDEENF
PROIMIA1     DDYDDGFGQTSSYHSARSSGGNNDRFANGTSYQKPQQSAVKQPSGFSSHPLFASSDEENF
CP3          DDYDDGFGQTSSYHSARSSGGNNDRFANGTSYQKPQQSAVKQPSGFSSHPLFASSDEENF
B12526       DDYDDGFGQTSSYHSARSSGGNNDRFANGTSYQKPQQSAVKQPSGFSSHPLFASSDEENF
CP4          DDYDDGFGQTSSYHSARSSGGNNDRFANGTSYQKPQQSAVKQPSGFSSHPLFASSDEENF
CP1          DDYDDGFGQTSSYHSARSSGGNNDRFANGTSYQKPQQPAVKQPSGFSSHPLFASSDEENF
B1960        DDYDDGFGQTSSYHSARSSGGNNDRFANGTSYQKPQQPAVKQPSGFSSHPLFASSDEENF
ATCC31822    DDYDDGFGQTSSYHSARSSGGNNDRFANGTSYQKPQQPAVKQPSGFSSHPLFASSDEENF
```

```
                   790       800        810            820       830
ZM4          HNRSEEKPASHHSYSSRQALA.ADTTDYNEGRKAAHTVSS...SSRKTQTSQPLLANNFT
B23394       HNRSEEKPASHHSYSSRQALA.ADTTDYNEGRKAAHTVSS...SSRKTQTSQPLLANNFT
Z6           HNRSEEKPASHHSYSSRQALA.ADTTDYNEGRKAAHTVSS...SSRKTQTSQPLLANNFT
B4492        HNRSEEKPASHHSYSSRQALA.ADTTDYNEGRKAAHTVSS...SSRKTQTSQPLLANNFT
ATCC10988    HNRSEEKSASRHSYSSRQGLAAADNTDYNEGRKAAHTVSSS..SSKKTQTSQPLLANNFT
CU1          HNRSEEKSASRHSYSSRQGLAAADNTDYNEGRKAAHTVSSS..SSKKTQTSQPLLANNFT
CU1rif2      HNRSEEKSASRHSYSSRQGLAAADNTDYNEGRKAAHTVSSS..SSKKTQTSQPLLANNFT
uvs51        HNRSEEKSASRHSYSSRQGLAAADNTDYNEGRKAAHTVSSS..SSKKTQTSQPLLANNFT
NCIMB11163   HNRSEEKPASRHSYSSRQGLAAADNTDYNEGRKAAHTVSSS..SSKKTQTSQPLLANNFT
PROIMIA1     HNRSEEKPASRHSYSSRQGLAAADNTDYNEGRKAAHTVSSS..SSKKTQTSQPLLANNFT
CP3          HNRSEEKPASRHSYSRRQGLAAADNTDYNEGRKAAHTVSSS..SSKKTQTSQPLLANNFT
B12526       HNRSEEKPASRHSYSRRQGLAAADNTDYNEGRKAAHTVSSS..SSKKTQTSQPLLANNFT
CP4          HNRSEEKPASRHSYSRRQGLAAADNTDYNEGRKAAHTVSSS..SSKKTQTSQPLLANNFT
CP1          HNRSEEKLASRHSYSSRQGLAAADNTDYNEGRKAAHTVSSSSSSSKKTQTSQPLLANNFT
B1960        HNRSEEKLASRHSYSSRQGLAAADNTDYNEGRKAAHTVSSSSSSSKKTQTSQPLLANNFT
ATCC31822    HNRSEEKPASRHSYSSRQGLAAADNTDYNEGRKAAHTVSSSSSSSKKTQTSQPLLANNFT
```

```
               840       850       860       870       880       890
ZM4          STQNALPFPNQTTGSRSSGNGLDSLNTRVFPDRNLSNDYDDEDERQEKIKRDMAEQRKYD
B23394       STQNALPFPNQTTGSRSSGNGLDSLNTRVFPDRNLSNDYDDEDERQEKIKRDMAEQRKYD
Z6           STQNALPFPNQTTGSRSSGNGLDSLNTRVFPDRNLSNDYDDEDERQEKIKRDMAEQRKYD
B4492        STQNALPFPNQTTGSRSSGNGLDSLNTRVFPDRNLSNDYDDEDERQEKIKRDMAEQRKYD
ATCC10988    STQNALPFPNQTTGSRSSGNGLDSLNTRVFPDRNLSNDYDDEDERQEKIKRDMAEQRKYD
CU1          STQNALPFPNQTTGSRSSGNGLDSLNTRVFPDRNLSNDYDDEDERQEKIKRDMAEQRKYD
CU1rif2      STQNALPFPNQTTGSRSSGNGLDSLNTRVFPDRNLSNDYDDEDERQEKIKRDMAEQRKYD
uvs51        STQNALPFPNQTTGSRSSGNGLDSLNTRVFPDRNLSNDYDDEDERQEKIKRDMAEQRKYD
NCIMB11163   STQNALPFPNQTTGSRSSGNGLDSLNTRVFPDRNLSNDYDDEDERQEKIKRDMAEQRKYD
PROIMIA1     STQNALPFPNQTTGSRSSGNGLDSLNTRVFPDRNLSNDYDDEDERQEKIKRDMAEQRKYD
CP3          STQNALPFPNQTTGSRSSGNGLDSLNTRVFPDRNLSNDYDDEDERQEKIKRDMAEQRKYD
B12526       STQNALPFPNQTTGSRSSGNGLDSLNTRVFPDRNLSNDYDDEDERQEKIKRDMAEQRKYD
CP4          STQNALPFPNQTTGSRSSGNGLDSLNTRVFPDRNLSNDYDDEDERQEKIKRDMAEQRKYD
CP1          STQNALPFPNQTTGSRSSGNGLDSLNTRVFPDRNLSNDYDDEDERQEKIKRDMAEQRKYD
B1960        STQNALPFPNQTTGSRSSGNGLDSLNTRVFPDRNLSNDYDDEDERQEKIKRDMAEQRKYD
ATCC31822    STQNALPFPNQTTGSRSSGNGLDSLNTRVFPDRNLSNDYDDEDERQEKIKRDMAEQRKYD
```

```
               900       910       920       930       940       950
ZM4          SWNIFAPGSAFGQSTMLEAPSYTSIAVADPINYPAAARLHGGSHDPLLRDINDGIDHLSL
B23394       SWNIFAPGSAFGQSTMLEAPSYTSIAVADPINYPAAARLHGGSHDPLLRDINDGIDHLSL
Z6           SWNIFAPGSAFGQSTMLEAPSYTSIAVADPINYPAAARLHGGSHDPLLRDINDGIDHLSL
B4492        SWNIFAPGSAFGQSTMLEAPSYTSIAVADPINYPAAARLHGGSHDPLLRDINDGIDHLSL
ATCC10988    SWNIFSPGSAFGQSTMLEAPSYTSIAVADPINYPAAARLHGGSHDPLLRDINDGIDHLSL
CU1          SWNIFSPGSAFGQSTMLEAPSYTSIAVADPINYPAAARLHGGSHDPLLRDINDGIDHLSL
CU1rif2      SWNIFSPGSAFGQSTMLEAPSYTSIAVADPINYPAAARLHGGSHDPLLRDINDGIDHLSL
uvs51        SWNIFSPGSAFGQSTMLEAPSYTSIAVADPINYPAAARLHGGSHDPLLRDINDGIDHLSL
NCIMB11163   SWNIFAPGSAFGQSTMLEAPSYTSIAVADPINYPAAARLHGGSHDPLLRDINDGIDHLSL
PROIMIA1     SWNIFAPGSAFGQSTMLEAPSYTSIAVADPINYPAAARLHGGSHDPLLRDINDGIDHLSL
CP3          SWNIFSPGSAFGQSTMLEAPSYTSIAVADPINYPAAARLHGGSHDPLLRDINDGIDHLSL
B12526       SWNIFSPGSAFGQSTMLEAPSYTSIAVADPINYPAAARLHGGSHDPLLRDINDGIDHLSL
CP4          SWNIFSPGSAFGQSTMLEAPSYTSIAVADPINYPAAARLHGGSHDPLLRDINDGIDHLSL
CP1          SWNIFSPGSAFGQSTMLEAPSYTSIAVADPINYPAAARLHGGSHDPLLRDINDGIDHLSL
B1960        SWNIFSPGSAFGQSTMLEAPSYTSIAVADPINYPAAARLHGGSHDPLLRDINDGIDHLSL
ATCC31822    SWNIFSPGSAFGQSTMLEAPSYTSIAVADPINYPAAARLHGGSHDPLLRDINDGIDHLSL
```

```
             960       970       980       990       1000      1010
ZM4          NTGTVLEGTPEFRQHSGQNGLSRLNEYAFNAKVSTNLGSRVRGFFSVTPVYLTAGQPDQY
B23394       NTGTVLEGTPEFRQHSGQNGLSRLNEYAFNAKVSTNLGSRVRGFFSVTPVYLTAGQPDQY
Z6           NTGTVLEGTPEFRQHSGQNGLSRLNEYAFNAKVSTNLGSRVRGFFSVTPVYLTAGQPDQY
B4492        NTGTVLEGTPEFRQHSGQNGLSRLNEYAFNAKVSTNLGSRVRGFFSVTPVYLTAGQPDQY
ATCC10988    NTGTVLEGTPEFRQHSGQNGLSRLNEYAFNAKVSTNLGSRVRGFFSVTPVYLTAGQPDQY
CU1          NTGTVLEGTPEFRQHSGQNGLSRLNEYAFNAKVSTNLGSRVRGFFSVTPVYLTAGQPDQY
CU1rif2      NTGTVLEGTPEFRQHSGQNGLSRLNEYAFNAKVSTNLGSRVRGFFSVTPVYLTAGQPDQY
uvs51        NTGTVLEGTPEFRQHSGQNGLSRLNEYAFNAKVSTNLGSRVRGFFSVTPVYLTAGQPDQY
NCIMB11163   NTGTVLEGTPEFRQHSGQNGLSRLNEYAFNAKVSTNLGSRVRGFFSVTPVYLTAGQPDKY
PROIMIA1     NTGTVLEGTPEFRQHSGQNGLSRLNEYAFNAKVSTNLGSRVRGFFSVTPVYLTAGQPDQY
CP3          NTGTVLEGTPEFRQHSGQNGLSRLNEYAFNAKVSTNLGSRVRGFFSVTPVYLTAGQPDQY
B12526       NTGTVLEGTPEFRQHSGQNGLSRLNEYAFNAKVSTNLGSRVRGFFSVTPVYLTAGQPDQY
CP4          NTGTVLEGTPEFRQHSGQNGLSRLNEYAFNAKVSTNLGSRVRGFFSVTPVYLTAGQPDQY
CP1          NTGTVLEGTPEFRQHSGQNGLSRLNEYAFNAKVSTNLGSRVRGFFSVTPVYLTAGQPDQY
B1960        NTGTVLEGTPEFRQHSGQNGLSRLNEYAFNAKVSTNLGSRVRGFFSVTPVYLTAGQPDQY
ATCC31822    NTGTVLEGTPEFRQHSGQNGLSRLNEYAFNAKVSTNLGSRVRGFFSVTPVYLTAGQPDQY
```

```
             1020      1030      1040      1050      1060      1070
ZM4          AAPYFGINPLKSTSSIASSGGTAQYAPVKDQSASGVALDAGIKIKDIRLDIGTTPIGFRKT
B23394       AAPYFGINPLKSTSSIASSGGTAQYAPVKDQSASGVALDAGIKIKDIRLDIGTTPIGFRKT
Z6           AAPYFGINPLKSTSSIASSGGTAQYAPVKDQSASGVALDAGIKIKDIRLDIGTTPIGFRKT
B4492        AAPYFGINPLKSTSSIASSGGTAQYAPVKDQSASGVALDAGIKIKDIRLDIGTTPIGFRKT
ATCC10988    AAPYFGSNPLVSNASIAAGGKAMYAPVKDQSASGVALDAGIKIKDIRLDIGTTPIGFRKT
CU1          AAPYFGSNPLVSNASIAAGGKAMYAPVKDQSASGVALDAGIKIKDIRLDIGTTPIGFRKT
CU1rif2      AAPYFGSNPLVSNASIAAGGKAMYAPVKDQSASGVALDAGIKIKDIRLDIGTTPIGFRKT
uvs51        AAPYFGSNPLVSNASIAAGGKAMYAPVKDQSASGVALDAGIKIKDIRLDIGTTPIGFRKT
NCIMB11163   AAPYFGSNPLVSNASIAAGGKAMYAPVKDQSASGVALDAGIKIKDIRLDIGTTPIGFRKT
PROIMIA1     AAPYFGSNPLVSNASIAAGGKAMYAPVKDQSASGVALDAGIKIKDIRLDIGTTPIGFRKT
CP3          AAPYFGSNPLVSNASIAAGGKAMYAPVKDQSASGVALDAGIKIKDIRLDIGTTPIGFRKT
B12526       AAPYFGSNPLVSNASIAAGGKAMYAPVKDQSASGVALDAGIKIKDIRLDIGTTPIGFRKT
CP4          AAPYFGSNPLVSNASIAAGGKAMYAPVKDQSASGVALDAGIKIKDIRLDIGTTPIGFRKT
CP1          AAPYFGSNPLVSNASIAAGGKAMYAPVKDQSASGVALDAGIKIKDIRLDIGTTPIGFRKT
B1960        AAPYFGSNPLVSNASIAAGGKAMYAPVKDQSASGVALDAGIKIKDIRLDIGTTPIGFRKT
ATCC31822    AAPYFGSNPLVSNASIAAGGKAMYAPVKDQSASGVALDAGIKIKDIRLDIGTTPIGFRKT
```

```
             1080      1090      1100      1110      1120      1130
ZM4          NIQGGVSWSPQIIDHLTGHIFMERRPVMDSLIAYAGSVDPIMGLSWGSVMKTGGGGGLSY
B23394       NIQGGVSWSPQIIDHLTGHIFMERRPVMDSLIAYAGSVDPIMGLSWGSVMKTGGGGGLSY
Z6           NIQGGVSWSPQIIDHLTGHIFMERRPVMDSLIAYAGSVDPIMGLSWGSVMKTGGGGGLSY
B4492        NIQGGVSWSPQIIDHLTGHIFMERRPVMDSLIAYAGSVDPIMGLSWGSVMKTGGGGGLSY
ATCC10988    NIQGGVSWSPQIIDHLTGHIFMERRPVMDSLIAYAGTVDPVMGLTWGSVMKTGGGGGLSY
CU1          NIQGGVSWSPQIIDHLTGHIFMERRPVMDSLIAYAGTVDPVMGLTWGSVMKTGGGGGLSY
CU1rif2      NIQGGVSWSPQIIDHLTGHIFMERRPVMDSLIAYAGTVDPVMGLTWGSVMKTGGGGGLSY
uvs51        NIQGGVSWSPQIIDHLTGHIFMERRPVMDSLIAYAGTVDPVMGLTWGSVMKTGGGGGLSY
NCIMB11163   NIQGGVSWSPQIIDHLTGHIFMERRPVMDSLIAYAGTVDPVMGLTWGSVMKTGGGGGLSY
PROIMIA1     NIQGGVSWSPQIIDHLTGHIFMERRPVMDSLIAYAGTVDPVMGLTWGSVMKTGGGGGLSY
CP3          NIQGGVSWSPQIIDHLTGHIFMERRPVMDSLIAYAGTVDPVMGLTWGSVMKTGGGGGLSY
B12526       NIQGGVSWSPQIIDHLTGHIFMERRPVMDSLIAYAGTVDPVMGLTWGSVMKTGGGGGLSY
CP4          NIQGGVSWSPQIIDHLTGHIFMERRPVMDSLIAYAGTVDPVMGLTWGSVMKTGGGGGLSY
CP1          NIQGGVSWSPQIIDHLTGHIFMERRPVMDSLIAYAGTVDPVMGLTWGSVMKTGGGGGLSY
B1960        NIQGGVSWSPQIIDHLTGHIFMERRPVMDSLIAYAGTVDPVMGLTWGSVMKTGGGGGLSY
ATCC31822    NIQGGVSWSPQIIDHLTGHIFMERRPVMDSLIAYAGTVDPVMGLTWGSVMKTGGGGGLSY
```

```
             1140      1150      1160      1170      1180      1190
ZM4          DVNGSGLYAQGNYRVYNGTRVQKNHAVEANVGGYYSLINSQSANFSIGVNVNYQHYLNNQ
B23394       DVNGSGLYAQGNYRVYNGTRVQKNHAVEANVGGYYSLINSQSANFSIGVNVNYQHYLNNQ
Z6           DVNGSGLYAQGNYRVYNGTRVQKNHAVEANVGGYYSLINSQSANFSIGVNVNYQHYLNNQ
B4492        DVNGSGLYAQGNYRVYNGTRVQKNHAVEANVGGYYSLINSQSANFSIGVNVNYQHYLNNQ
ATCC10988    DVNGSGLYAQGNYRVYNGTRVQKNHAVEANVGGYYSLLNTPTSNFSIGVNVNYQHYLNNQ
CU1          DVNGSGLYAQGNYRVYNGTRVQKNHAVEANVGGYYSLLNTPTSNFSIGVNVNYQHYLNNQ
CU1rif2      DVNGSGLYAQGNYRVYNGTRVQKNHAVEANVGGYYSLLNTPTSNFSIGVNVNYQHYLNNQ
uvs51        DVNGSGLYAQGNYRVYNGTRVQKNHAVEANVGGYYSLLNTPTSNFSIGVNVNYQHYLNNQ
NCIMB11163   DVNGSGLYAQGNYRVYNGTRVQKNHAVEANVGGYYSLLNTPTSNFSIGVNVNYQHYLNNQ
PROIMIA1     DVNGSGLYAQGNYRVYNGTRVQKNHAVEANVGGYYSLLNTPTSNFSIGVNVNYQHYLNNQ
CP3          DVNGSGLYAQGNYRVYNGTRVQKNHAVEANVGGYYSLLNTPTSNFSIGVNVNYQHYLNNQ
B12526       DVNGSGLYAQGNYRVYNGTRVQKNHAVEANVGGYYSLLNTPTSNFSIGVNVNYQHYLNNQ
CP4          DVNGSGLYAQGNYRVYNGTRVQKNHAVEANVGGYYSLLNTPTSNFSIGVNVNYQHYLNNQ
CP1          DVNGSGLYAQGNYRVYNGTRVQKNHAVEANVGGYYSLLNTPTSNFSIGVNVNYQHYLNNQ
B1960        DVNGSGLYAQGNYRVYNGTRVQKNHAVEANVGGYYSLLNTPTSNFSIGVNVNYQHYLNNQ
ATCC31822    DVNGSGLYAQGNYRVYNGTRVQKNHAVEANVGGYYSLLNTPTSNFSIGVNVNYQHYLNNQ
```

```
            1200      1210      1220      1230      1240      1250
ZM4         YFFSFAQGGYFSPNHFISVAFPLRYTGTLGRWKLNAELAPGYQNFYERSNYIFANEHDLQ
B23394      YFFSFAQGGYFSPNHFISVAFPLRYTGTLGRWKLNAELAPGYQNFYERSNYIFANEHDLQ
Z6          YFFSFAQGGYFSPNHFISVAFPLRYTGTLGRWKLNAELAPGYQNFYERSNYIFANEHDLQ
B4492       YFFSFAQGGYFSPNHFISVAFPLRYTGTLGRWKLNAELAPGYQNFYERSNYIFANEHDLQ
ATCC10988   YFFSFAQGGYFSPNHFISVAFPLRYTGTLGRWKLNAELAPGYQNFYERSNYIFANEHDLQ
CU1         YFFSFAQGGYFSPNHFISVAFPLRYTGTLGRWKLNAELAPGYQNFYERSNYIFANEHDLQ
CU1rif2     YFFSFAQGGYFSPNHFISVAFPLRYTGTLGRWKLNAELAPGYQNFYERSNYIFANEHDLQ
uvs51       YFFSFAQGGYFSPNHFISVAFPLRYTGTLGRWKLNAELAPGYQNFYERSNYIFANEHDLQ
NCIMB11163  YFFSFAQGGYFSPNHFISVAFPLRYTGTLGRWKLNAELAPGYQNFYERSNYIFANEHDLQ
PROIMIA1    YFFSFAQGGYFSPNHFISVAFPLRYTGTLGRWKLNAELAPGYQNFYERSNYIFANEHDLQ
CP3         YFFSFAQGGYFSPNHFISVAFPLRYTGTLGRWKLNAELAPGYQNFYERSNYIFANEHDLQ
B12526      YFFSFAQGGYFSPNHFISVAFPLRYTGTLGRWKLNAELAPGYQNFYERSNYIFANEHDLQ
CP4         YFFSFAQGGYFSPNHFISVAFPLRYTGTLGRWKLNAELAPGYQNFYERSNYIFANEHDLQ
CP1         YFFSFAQGGYFSPNHFISVAFPLRYTGTLGRWKLNAELAPGYQNFYERSNYIFANEHDLQ
B1960       YFFSFAQGGYFSPNHFISVAFPLRYTGTLGRWKLNAELAPGYQNFYERSNYIFANEHDLQ
ATCC31822   YFFSFAQGGYFSPNHFISVAFPLRYTGTLGRWKLNAELAPGYQNFYERSNYIFANEHDLQ

            1260      1270      1280      1290      1300      1310
ZM4         NQMNKYNLANNSLANKFPGQHHSGAGYEGRLDVSYRLSRSAVLGGEIRATTFGPYSEYRE
B23394      NQMNKYNLANNSLANKFPGQHHSGAGYEGRLDVSYRLSRSAVLGGEIRATTFGPYSEYRE
Z6          NQMNKYNLANNSLANKFPGQHHSGAGYEGRLDVSYRLSRSAVLGGEIRATTFGPYSEYRE
B4492       NQMNKYNLANNSLANKFPGQHHSGAGYEGRLDVSYRLSRSAVLGGEIRATTFGPYSEYRE
ATCC10988   NQMNKFNLANNSLANKFPGQHHSGAGYEGRLAVSYRLSRSAVLGGEIRATTFGPYSEYRE
CU1         NQMNKFNLANNSLANKFPGQHHSGAGYEGRLAVSYRLSRSAVLGGEIRATTFGPYSEYRE
CU1rif2     NQMNKFNLANNSLANKFPGQHHSGAGYEGRLAVSYRLSRSAVLGGEIRATTFGPYSEYRE
uvs51       NQMNKFNLANNSLANKFPGQHHSGAGYEGRLAVSYRLSRSAVLGGEIRATTFGPYSEYRE
NCIMB11163  NQMNKFNLANNSLANKFPGQHHSGAGYEGRLAVSYRLSRSAVLGGEIRATTFGPYSEYRE
PROIMIA1    NQMNKFNLANNSLANKFPGQHHSGAGYEGRLAVSYRLSRSAVLGGEIRATTFGPYSEYRE
CP3         NQMNKFNLANNSLANKFPGQHHSGAGYEGRLAVSYRLSRSAVLGGEIRATTFGPYSEYRE
B12526      NQMNKFNLANNSLANKFPGQHHSGAGYEGRLAVSYRLSRSAVLGGEIRATTFGPYSEYRE
CP4         NQMNKFNLANNSLANKFPGQHHSGAGYEGRLAVSYRLSRSAVLGGEIRATTFGPYSEYRE
CP1         NQMNKFNLANNSLANKFPGQHHSGAGYEGRLAVSYRLSRSAVLGGKIRATTFGPYSEYRE
B1960       NQMNKFNLANNSLANKFPGQHHSGAGYEGRLAVSYRLSRSAVLGGKIRATTFGPYSEYRE
ATCC31822   NQMNKFNLANNSLANKFPGQHHSGAGYEGRLAVSYRLSRSAVLGGEIRATTFGPYSEYRE

            1320      1330
ZM4         LLSIHYAMDADKPDKH............................................
B23394      LLSIHYAMDADKPDKH............................................
Z6          LLSIHYAMDADKPDKH............................................
B4492       LLSIHYAMDADKPDKH............................................
ATCC10988   LLSIHYAMDADKTDKH............................................
CU1         LLSIHYAMDADKTDKH............................................
CU1rif2     LLSIHYAMDADKTDKH............................................
uvs51       LLSIHYAMDADKTDKH............................................
NCIMB11163  LLSIHYAMDADKTDKHFILSTLVSVPLLVACRKGKAAEADNWTLFKSRFFKDGRISDSGN
PROIMIA1    LLSIHYAMDADKTDKH............................................
CP3         LLSIHYAMDADKTDKH............................................
B12526      LLSIHYAMDADKTDKH............................................
CP4         LLSIHYAMDADKTDKH............................................
CP1         LLSIHYAMDADKTDKH............................................
B1960       LLSIHYAMDADKTDKH............................................
ATCC31822   LLSIHYAMDADKTDKH............................................

ZM4         ............................................................
B23394      ............................................................
Z6          ............................................................
B4492       ............................................................
ATCC10988   ............................................................
CU1         ............................................................
CU1rif2     ............................................................
uvs51       ............................................................
NCIMB11163  GNISHSEGQGYGMIQAEAAHDKATFDALWQWTKTHLMRPDMALFAWRFDPSQSNPVSDQN
PROIMIA1    ............................................................
CP3         ............................................................
B12526      ............................................................
CP4         ............................................................
CP1         ............................................................
B1960       ............................................................
ATCC31822   ............................................................
```

```
ZM4          ............................................................
B23394       ............................................................
Z6           ............................................................
B4492        ............................................................
ATCC10988    ............................................................
CU1          ............................................................
CU1rif2      ............................................................
uvs51        ............................................................
NCIMB11163   NATDGDILIAWALLRAEKRWPKNGYGQDSEAIRKSIGKKLVLSGGGETILLPGLQGFTGT
PROIMIA1     ............................................................
CP3          ............................................................
B12526       ............................................................
CP4          ............................................................
CP1          ............................................................
B1960        ............................................................
ATCC31822    ............................................................
```

```
ZM4          ............................................................
B23394       ............................................................
Z6           ............................................................
B4492        ............................................................
ATCC10988    ............................................................
CU1          ............................................................
CU1rif2      ............................................................
uvs51        ............................................................
NCIMB11163   DYVILNFSYYIWPALKAFNEADNGAWHNVIESGKKLLAKAKFGLPQLPTDWVAFKNNGNL
PROIMIA1     ............................................................
CP3          ............................................................
B12526       ............................................................
CP4          ............................................................
CP1          ............................................................
B1960        ............................................................
ATCC31822    ............................................................
```

```
ZM4          ............................................................
B23394       ............................................................
Z6           ............................................................
B4492        ............................................................
ATCC10988    ............................................................
CU1          ............................................................
CU1rif2      ............................................................
uvs51        ............................................................
NCIMB11163   EPAADKQPYFGFDAVRIPLYLIWGGEDALAAPFAIYWNSYLSHNQPVPAWVDVNSQAIAP
PROIMIA1     ............................................................
CP3          ............................................................
B12526       ............................................................
CP4          ............................................................
CP1          ............................................................
B1960        ............................................................
ATCC31822    ............................................................
```

```
ZM4          .................................................
B23394       .................................................
Z6           .................................................
B4492        .................................................
ATCC10988    .................................................
CU1          .................................................
CU1rif2      .................................................
uvs51        .................................................
NCIMB11163   YPLSKGGMAILDLAMNKPITAKIADQDDYYSSALLALSEIAAKERPHNR
PROIMIA1     .................................................
CP3          .................................................
B12526       .................................................
CP4          .................................................
CP1          .................................................
B1960        .................................................
ATCC31822    .................................................
```
