# Supplementary material for: Zymomonas diversity and potential for biofuel production
Source: Biotechnol Biofuels. 2021 May 1;14:112. doi: 10.1186/s13068-021-01958-2 (PMC8088579; doi:10.1186/s13068-021-01958-2)
Supplement: Supplementary file 4 — Additional file 4: Figure S8. Z. mobilis mobilis BcsC alignment (see figure in separate PDF). BcsC (ZMO1085) alignment was performed as described in Figure S5 (BcsA). BcsC from pomaceae is a pseudogene and BcsC from francensis was omitted for clarity. BcsC of NCIMB11163 is fused in-frame with bcsZ. Only first residue of fused BcsCZ protein is shown. All colors are as in Figure S5. [file 13068_2021_1958_MOESM4_ESM.pdf]

```
              1        10        20        30        40        50        60
francensis    MLRKSRIKINKLLSEAKYILEHVWDSFLQWPLVFLIAAAAIMVISVPLPLYYQWVYGVFF
ATTC10988     MLHKSRIKIKNTLSEAKYILEHLWDSTLQWPLLCLITVAALMVVAVPLPLYYQWVYGIFF
CU1           MLHKSRIKIKNTLSEAKYILEHLWDSTLQWPLLCLITVAALMVVAVPLPLYYQWVYGIFF
CU1rif2       MLHKSRIKIKNTLSEAKYILEHLWDSTLQWPLLCLITVAALMVVAVPLPLYYQWVYGIFF
uvs51         MLHKSRIKIKNTLSEAKYILEHLWDSTLQWPLLCLITVAALMVVAVPLPLYYQWVYGIFF
B23394        MLHKSRIKIKNTLSEAKYILEHLWDSALQWPLLCLITVAALMVVAVPLPLYYQWIYGIFF
Z6            MLHKSRIKIKNTLSEAKYILEHLWDSALQWPLLCLITVAALMVVAVPLPLYYQWIYGIFF
B4492         MLHKSRIKIKNTLSEAKYILEHLWDSALQWPLLCLITVAALMVVAVPLPLYYQWIYGIFF
PROIMIA1      MLHKSRIKIKNTLSEAKYILEHLWDSTLQWPLLCLITVAALMVVAVPLPLYYQWVYGIFF
NCIMB11163    MLHKSRIKIKNTLSEAKYILEHLWDSALQWPLLCLITVAALMVVAVPLPLYYQWVYGIFF
CP1           MLHKSRIKIKNTLSEAKYILEHLWDSALQWPLLCLITVAALMVVAVPLPLYYQWVYGIFF
B1960         MLHKSRIKIKNTLSEAKYILEHLWDSALQWPLLCLITVAALMVVAVPLPLYYQWVYGIFF
ATCC31822     MLHKSRIKIKNTLSEAKYILEHLWDSALQWPLLCLITVAALMVVAVPLPLYYQWVYGIFF
CP4           .........................................MVVAVPLPLYYQWVYGIFF
B12526        MLHKSRIKIKNTLSEAKYILEHLWDSTLQWPLLCLITVAALMVVAVPLPLYYQWVYGIFF
CP3           MLHKSRIKIKNTLSEAKYILEHLWDSTLQWPLLCLITVAALMVVAVPLPLYYQWVYGIFF

                       70        80        90       100       110       120
francensis    MGLTLLIDRNPSHYASIVICLSSILTSTRYIFWRITQTLRFDHIMDAIFGSILFMAELYA
ATTC10988     MGLTLLIDRSPSHFASIVICLSSILTSTRYIFWRITQTLRFDHIMDAVFGGVLFMAELYA
CU1           MGLTLLIDRSPSHFASIVICLSSILTSTRYIFWRITQTLRFDHIMDAVFGGVLFMAELYA
CU1rif2       MGLTLLIDRSPSHFASIVICLSSILTSTRYIFWRITQTLRFDHIMDAVFGGVLFMAELYA
uvs51         MGLTLLIDRSPSHFASIVICLSSILTSTRYIFWRITQTLRFDHIMDAVFGGVLFMAELYA
B23394        MGLTLLIDCSPSHFASIVICLSSILTSTRYIFWRITQTLRFDHIMDAVFGGVLFMAELYA
Z6            MGLTLLIDCSPSHFASIVICLSSILTSTRYIFWRITQTLRFDHIMDAVFGGVLFMAELYA
B4492         MGLTLLIDCSPSHFASIVICLSSILTSTRYIFWRITQTLRFDHIMDAVFGGVLFMAELYA
PROIMIA1      MGLTLLIDRSPSHFASIVICLSSILTSTRYIFWRITQTLRFDHIMDAVFGGVLFMAELYA
NCIMB11163    MGLTLLIDRSPSHFASIVICLSSILTSTRYIFWRITQTLRFDHIMDAVFGGVLFMAELYA
CP1           MGLTLLIDRSPSHFASIVICLSSILTSTRYIFWRITQTLRFDHIMDAVFGGVLFMAELYA
B1960         MGLTLLIDRSPSHFASIVICLSSILTSTRYIFWRITQTLRFDHIMDAVFGGVLFMAELYA
ATCC31822     MGLTLLIDRSPSHFASIVICLSSILTSTRYIFWRITQTLRFDHIMDAVFGGVLFMAELYA
CP4           MGLTLLIDRSPSHFASIVICLSSILTSTRYIFWRITQTLRFDHIMDAVFGGVLFMAELYA
B12526        MGLTLLIDRSPSHFASIVICLSSILTSTRYIFWRITQTLRFDHIMDAVFGGVLFMAELYA
CP3           MGLTLLIDRSPSHFASIVICLSSILTSTRYIFWRITQTLRFDHIMDAVFGGVLFMAELYA

                      130       140       150       160       170       180
francensis    WAILILGLFQILWPMQRPVIPLSGKDEDLPTIDVFIPTFNESMEIVRNTVFAAMGMDYPK
ATTC10988     WAILILGLFQILWPMQRPVVPLSGEDEELPTVDVFIPTYNESMEIVQNTVFAALGMDYPK
CU1           WAILILGLFQILWPMQRPVVPLSGEDEELPTVDVFIPTYNESMEIVQNTVFAALGMDYPK
CU1rif2       WAILILGLFQILWPMQRPVVPLSGEDEELPTVDVFIPTYNESMEIVQNTVFAALGMDYPK
uvs51         WAILILGLFQILWPMQRPVVPLSGEDEELPTVDVFIPTYNESMEIVQNTVFAALGMDYPK
B23394        WAILILGLFQILWPMQRPVVPLSGGDEDLPTVDVFIPTYNESMEIVQNTVFAALGMDYPK
Z6            WAILILGLFQILWPMQRPVVPLSGGDEDLPTVDVFIPTYNESMEIVQNTVFAALGMDYPK
B4492         WAILILGLFQILWPMQRPVVPLSGGDEDLPTVDVFIPTYNESMEIVQNTVFAALGMDYPK
PROIMIA1      WAILILGLFQILWPMQRPVVPLSGEDEELPTVDVFIPTYNESMEIVQNTVFAALGMDYPK
NCIMB11163    WAILILGLFQILWPMQRPVVPLSGEDEELPTVDVFIPTYNESMEIVQNTVFAALGMDYPK
CP1           WAILILGLFQILWPMQRPVVPLSGEDEELPTVDVFIPTYNESMEIVQNTVFAALGMDYPK
B1960         WAILILGLFQILWPMQRPVVPLSGEDEELPTVDVFIPTYNESMEIVQNTVFAALGMDYPK
ATCC31822     WAILILGLFQILWPMQRPVVPLSGEDEELPTVDVFIPTYNESMEIVQNTVFAALGMDYPK
CP4           WAILILGLFQILWPMQRPVVPLSGEDEELPTVDVFIPTYNESMEIVQNTVFAALGMDYPK
B12526        WAILILGLFQILWPMQRPVVPLSGEDEELPTVDVFIPTYNESMEIVQNTVFAALGMDYPK
CP3           WAILILGLFQILWPMQRPVVPLSGEDEELPTVDVFIPTYNESMEIVQNTVFAALGMDYPK

                      190       200       210       220       230       240
francensis    DRFKVYLLDDGNREEFRIFAEQAGCNYLTRTDNLNAKAGNLNAALKKTSGELVCIFDCDH
ATTC10988     DRFNVYLLDDGHREEFRLFAEEAGCHYLTRNDNLNAKAGNLNAALKKTKGELVCIFDCDH
CU1           DRFNVYLLDDGHREEFRLFAEEAGCHYLTRNDNLNAKAGNLNAALKKTKGELVCIFDCDH
CU1rif2       DRFNVYLLDDGHREEFRLFAEEAGCHYLTRNDNLNAKAGNLNAALKKTKGELVCIFDCDH
uvs51         DRFNVYLLDDGHREEFRLFAEEAGCHYLTRNDNLNAKAGNLNAALKKTKGELVCIFDCDH
B23394        DRFNVYLLDDGHREEFRLFAEEAGCHYLTRNDNLNAKAGNLNAALKKTKGELVCIFDCDH
Z6            DRFNVYLLDDGHREEFRLFAEEAGCHYLTRNDNLNAKAGNLNAALKKTKGELVCIFDCDH
B4492         DRFNVYLLDDGHREEFRLFAEEAGCHYLTRNDNLNAKAGNLNAALKKTKGELVCIFDCDH
PROIMIA1      DRFNVYLLDDGHREEFRLFAEEAGCHYLTRNDNLNAKAGNLNAALKKTKGELVCIFDCDH
NCIMB11163    DRFNVYLLDDGHREEFRLFAEEAGCHYLTRNDNLNAKAGNLNAALRKTKGELVCIFDCDH
CP1           DRFNVYLLDDGHREEFRLFAEEAGCHYLTRNDNLNAKAGNLNAALRKTKGELVCIFDCDH
B1960         DRFNVYLLDDGHREEFRLFAEEAGCHYLTRNDNLNAKAGNLNAALRKTKGELVCIFDCDH
ATCC31822     DRFNVYLLDDGHREEFRLFAEEAGCHYLTRNDNLNAKAGNLNAALKKTKGELVCIFDCDH
CP4           DRFNIYLLDDGHREEFRLFAEEAGCHYLTRNDNLNAKAGNLNAALKKTKGELVCIFDCDH
B12526        DRFNIYLLDDGHREEFRLFAEEAGCHYLTRNDNLNAKAGNLNAALKKTKGELVCIFDCDH
CP3           DRFNIYLLDDGHREEFRLFAEEAGCHYLTRNDNLNAKAGNLNAALKKTKGELVCIFDCDH
```

```
                           250       260       270       280       290       300
francensis   VPTRAFLQLTVGWLQKEPNLALVQTPHFFYSPDPIQKNVPGGSELPGDNELFYGTVQRGN
ATTC10988    VPTRAFLQLTVGWLQKEPNLALVQTPHFFYSPDPIQRNVPGGDELPGDNELFYGTVQRGN
CU1          VPTRAFLQLTVGWLQKEPNLALVQTPHFFYSPDPIQRNVPGGDELPGDNELFYGTVQRGN
CU1rif2      VPTRAFLQLTVGWLQKEPNLALVQTPHFFYSPDPIQRNVPGGDELPGDNELFYGTVQRGN
uvs51        VPTRAFLQLTVGWLQKEPNLALVQTPHFFYSPDPIQRNVPGGDELPGDNELFYGTVQRGN
B23394       VPTRAFLQLTVGWLQKEPNLALVQTPHFFYSPDPIQRNVPGGDELPGDNELFYGTVQRGN
Z6           VPTRAFLQLTVGWLQKEPNLALVQTPHFFYSPDPIQRNVPGGDELPGDNELFYGTVQRGN
B4492        VPTRAFLQLTVGWLQKEPNLALVQTPHFFYSPDPIQRNVPGGDELPGDNELFYGTVQRGN
PROIMIA1     VPTRAFLQLTVGWLQKEPNLALVQTPHFFYSPDPIQRNVPGGDELPGDNELFYGTVQRGN
NCIMB11163   VPTRAFLQLTVGWLQKEPNLALVQTPHFFYSPDPIQRNVPGGDELPGDNELFYGTVQRGN
CP1          VPTRAFLQLTVGWLQKEPNLALVQTPHFFYSPDPIQRNVPGGDELPGDNELFYGTVQRGN
B1960        VPTRAFLQLTVGWLQKEPNLALVQTPHFFYSPDPIQRNVPGGDELPGDNELFYGTVQRGN
ATCC31822    VPTRAFLQLTVGWLQKEPNLALVQTPHFFYSPDPIQRNVPGGDELPGDNELFYGTVQRGN
CP4          VPTRAFLQLTVGWLQKEPNLALVQTPHFFYSPDPIQRNVPGGDELPGDNELFYGTVQRGN
B12526       VPTRAFLQLTVGWLQKEPNLALVQTPHFFYSPDPIQRNVPGGDELPGDNELFYGTVQRGN
CP3          VPTRAFLQLTVGWLQKEPNLALVQTPHFFYSPDPIQRNVPGGDELPGDNELFYGTVQRGN
```

```
                           310       320       330       340       350       360
francensis   DLWDATFFCGSCAILRRAALEENDGFSGETVTEDAHTALRIQRRGWDTAYINIRLSAGLA
ATTC10988    DLWDATFFCGSCAILRREALEENDGFSGETVTEDAHTALKLQRRGWDTAYINIRLSAGLA
CU1          DLWDATFFCGSCAILRREALEENDGFSGETVTEDAHTALKLQRRGWDTAYINIRLSAGLA
CU1rif2      DLWDATFFCGSCAILRREALEENDGFSGETVTEDAHTALKLQRRGWDTAYINIRLSAGLA
uvs51        DLWDATFFCGSCAILRREALEENDGFSGETVTEDAHTALKLQRRGWDTAYINIRLSAGLA
B23394       DLWDATFFCGSCAILRREALEENNGFSGETVTEDAHTALKLQRRGWDTAYINIRLSAGLA
Z6           DLWDATFFCGSCAILRREALEENNGFSGETVTEDAHTALKLQRRGWDTAYINIRLSAGLA
B4492        DLWDATFFCGSCAILRREALEENNGFSGETVTEDAHTALKLQRRGWDTAYINIRLSAGLA
PROIMIA1     DLWDATFFCGSCAILRREALEENDGFSGETVTEDAHTALKLQRRGWDTAYINIRLSAGLA
NCIMB11163   DLRDATFFCGSCAILRREALEENNGFSGETVTEDAHTALKLQRRGWDTAYINIRLSAGLA
CP1          DLWDATFFCGSCAILRREALEENNGFSGETVTEDAHTALKLQRRGWDTAYINIRLSAGLA
B1960        DLWDATFFCGSCAILRREALEENNGFSGETVTEDAHTALKLQRRGWDTAYINIRLSAGLA
ATCC31822    DLWDATFFCGSCAILRREALEENNGFSGETVTEDAHTALKLQRRGWDTAYINIRLSAGLA
CP4          DLWDATFFCGSCAILRREALEENNGFSGETVTEDAHTALKLQRRGWDTAYINIRLSAGLA
B12526       DLWDATFFCGSCAILRREALEENNGFSGETVTEDAHTALKLQRRGWDTAYINIRLSAGLA
CP3          DLWDATFFCGSCAILRREALEENNGFSGETVTEDAHTALKLQRRGWDTAYINIRLSAGLA
```

```
                           370       380       390       400       410       420
francensis   TDTLLAHIKQRARWARGMTQILRVDNPLWGKGLTIAQRLCYMNALLHYQFALPRVIFLIS
ATTC10988    TDTLLAHIKQRARWARGMTQILRIDNPLWGRGLTIAQRLCYMNALLHYQFALPRVIFLIS
CU1          TDTLLAHIKQRARWARGMTQILRIDNPLWGRGLTIAQRLCYMNALLHYQFALPRVIFLIS
CU1rif2      TDTLLAHIKQRARWARGMTQILRIDNPLWGRGLTIAQRLCYMNALLHYQFALPRVIFLIS
uvs51        TDTLLAHIKQRARWARGMTQILRIDNPLWGRGLTIAQRLCYMNALLHYQFALPRVIFLIS
B23394       TDTLLAHIKQRARWARGMTQILRIDNPLWGRGLTIAQRLCYMNALLHYQFALPRVIFLIS
Z6           TDTLLAHIKQRARWARGMTQILRIDNPLWGRGLTIAQRLCYMNALLHYQFALPRVIFLIS
B4492        TDTLLAHIKQRARWARGMTQILRIDNPLWGRGLTIAQRLCYMNALLHYQFALPRVIFLIS
PROIMIA1     TDTLLAHIKQRARWARGMTQILRIDNPLWGRGLTIAQRLCYMNALLHYQFALPRVIFLIS
NCIMB11163   TDTLLAHIKQRARWARGMTQILRIDNPLWGRGLTIAQRLCYMNALLHYQFALPRVIFLIS
CP1          TDTLLAHIKQRARWARGMTQILRIDNPLWGRGLTIAQRLCYMNALLHHQFALPRVIFLIS
B1960        TDTLLAHIKQRARWARGMTQILRIDNPLWGRGLTIAQRLCYMNALLHHQFALPRVIFLIS
ATCC31822    TDTLLAHIKQRARWARGMTQILRIDNPLWGRGLTIAQRLCYMNALLHYQFALPRVIFLIS
CP4          TDTLLAHIKQRARWARGMTQILRIDNPLWGRGLTIAQRLCYMNALLHYQFALPRVIFLIS
B12526       TDTLLAHIKQRARWARGMTQILRIDNPLWGRGLTIAQRLCYMNALLHYQFALPRVIFLIS
CP3          TDTLLAHIKQRARWARGMTQILRIDNPLWGRGLTIAQRLCYMNALLHYQFALPRVIFLIS
```

```
                           430       440       450       460       470       480
francensis   PLAFILFNSSIIHSSAVMVFAYAIPHLFMSLWAHERLSSGRRQPFWGEINETLLAFHLIK
ATTC10988    PLAFILFNSSIIHASAIMVFAYVVPHLFMSLWAHERLSNGRRQPFWGEINETLLAFHLIK
CU1          PLAFILFNSSIIHASAIMVFAYVVPHLFMSLWAHERLSNGRRQPFWGEINETLLAFHLIK
CU1rif2      PLAFILFNSSIIHASAIMVFAYVVPHLFMSLWAHERLSNGRRQPFWGEINETLLAFHLIK
uvs51        PLAFILFNSSIIHASAIMVFAYVVPHLFMSLWAHERLSNGRRQPFWGEINETLLAFHLIK
B23394       PLAFILFNSSIIHASAVMVFAYAIPHLFMSLWAHERLSNGRRQPFWSEINETLLAFHLIK
Z6           PLAFILFNSSIIHASAVMVFAYAIPHLFMSLWAHERLSNGRRQPFWSEINETLLAFHLIK
B4492        PLAFILFNSSIIHASAVMVFAYAIPHLFMSLWAHERLSNGRRQPFWSEINETLLAFHLIK
PROIMIA1     PLAFILFNSSIIHASAVMVFAYAIPHLFMSLWAHERLSNGRRQPFWGEINETLLAFHLIK
NCIMB11163   PLAFILFNSSIIHASAVMVFAYAIPHLFMSLWAHERLSNGRRQPFWGEINETLLAFHLIK
CP1          PLAFILFNSSIIHASAVMVFAYAIPHLFMSLSAHERLSNGRRQPFWGEINETLLAFHLIK
B1960        PLAFILFNSSIIHASAVMVFAYAIPHLFMSLSAHERLSNGRRQPFWGEINETLLAFHLIK
ATCC31822    PLAFILFNSSIIHASAVMVFAYAIPHLFMSLWAHERLSNGRRQPFWGEINETLLAFHLIK
CP4          PLAFILFNSSIIHASAVMVFAYAIPHLFMSLWAHERLSNGRRQPFWGEINETLLAFHLIK
B12526       PLAFILFNSSIIHASAVMVFAYAIPHLFMSLWAHERLSNGRRQPFWGEINETLLAFHLIK
CP3          PLAFILFNSSIIHASAVMVFAYAIPHLFMSLWAHERLSNGRRQPFWGEINETLLAFHLIK
```

```
                                  490       500       510       520       530       540
francensis   PTLITIFNPKKGKFNVTDKGERSEDDYFDLRSVRPHLITTAFLFLGLVVGTIKLIYADYF
ATTC10988    PTLITFLNPKKGSFNVTDKGERMEDDYFDFQSARPHIITAGFLFFGLIVGIVKLIYSSYF
CU1          PTLITFLNPKKGSFNVTDKGERMEDDYFDFQSARPHIITAGFLFFGLIVGIVKLIYSSYF
CU1rif2      PTLITFLNPKKGSFNVTDKGERMEDDYFDFQSARPHIITAGFLFFGLIVGIVKLIYSSYF
uvs51        PTLITFLNPKKGSFNVTDKGERMEDDYFDFQSARPHIITAGFLFFGLIVGIVKLIYSSYF
B23394       PTLITFFNPKKGSFNVTDKGERMEDDYFDIRSVRPHLITAGFLFLGLVVGIVKLIYSSYF
Z6           PTLITFFNPKKGSFNVTDKGERMEDDYFDIRSVRPHLITAGFLFLGLVVGIVKLIYSSYF
B4492        PTLITFFNPKKGSFNVTDKGERMEDDYFDIRSVRPHLITAGFLFLGLVVGIVKLIYSSYF
PROIMIA1     PTLITFLNPKKGSFNVTDKGERMEDDYFDIRSVRPHLITAGFLFLGLVVGIVKLIYSSYF
NCIMB11163   PTLITLSYPKKGSFNVTDKGERMEDDYFDIRSVRPHLITAGFLFLGLVVGIVKLIYSSYF
CP1          PTLITFLNPKKGSFNVTDKGERMEDDYFDIRSVRPHLITAGFLFLGLVVGIVKLIYSSYF
B1960        PTLITFLNPKKGSFNVTDKGERMEDDYFDIRSVRPHLIIAGFLFLGLVVGIVKLIYSSYF
ATCC31822    PTLITFLNPKKGSFNVTDKGERMEDDYFDIRSVRPHLITAGFLFLGLVVGIVKLIYSSYF
CP4          PTLITFLNPKKGSFNVTDKGERMEDDYFDIRSVRPHLITAGFLFLGLVVGIVKLIYSSYF
B12526       PTLITFLNPKKGSFNVTDKGERMEDDYFDIRSVRPHLITAGFLFLGLVVGIVKLIYSSYF
CP3          PTLITFLNPKKGSFNVTDKGERMEDDYFDIRSVRPHLITAGFLFLGLVVGIVKLIYSSYF

                                  550       560       570       580       590       600
francensis   HIQSSVLVLNILWASFNFIILLASVAVANESRQVRNTVRFPFQIPFSAYFDDGHVIDSVT
ATTC10988    HIQPSVLVLNVTWASFNFIILLASIAVAHESRQIRNTVRFPFRIPFTAYFEDGHVIDSVT
CU1          HIQPSVLVLNVTWASFNFIILLASIAVAHESRQIRNTVRFPFRIPFTAYFEDGHVIDSVT
CU1rif2      HIQPSVLVLNVTWASFNFIILLASIAVAHESRQIRNTVRFPFRIPFTAYFEDGHVIDSVT
uvs51        HIQPSVLVLNVTWASFNFIILLASIAVAHESRQIRNTVRFPFRIPFTAYFEDGHVIDSVT
B23394       HIQPSVLVLNVTWASFNFIILLASIAVAHESRQIRNTVRFPFRIPFTAYFEDGHVIDSVT
Z6           HIQPSVLVLNVTWASFNFIILLASIAVAHESRQIRNTVRFPFRIPFTAYFEDGHVIDSVT
B4492        HIQPSVLVLNVTWASFNFIILLASIAVAHESRQIRNTVRFPFRIPFTAYFEDGHVIDSVT
PROIMIA1     HIQPSVLVLNVTWASFNFIILLASIAVAHESRQIRNTVRFPFRIPFTAYFEDGHVIDSVT
NCIMB11163   HIQPSVLVLNVTWASFNFIILLASIAVAHESRQIRNTVRFPFRIPFTAYFEDGHVIDSVT
CP1          HIQPSVLVLNVTWASFNFIILLASIAVAHESRQIRNTVRFPFRIPFTAYFEDGHVIDSVT
B1960        HIQPSVLVLNVTWASFNFIILLASIAVAHESRQIRNTVRFPFRIPFTAYFEDGHVIDSVT
ATCC31822    HIQPSVLVLNVTWASFNFIILLASIAVAHESRQIRNTVRFPFRIPFTAYFEDGHVIDSVT
CP4          HIQPSVLVLNVTWASFNFIILLASIAVAHESRQIRNTVRFPFRIPFTAYFEDGHVIDSVT
B12526       HIQPSVLVLNVTWASFNFIILLASIAVAHESRQIRNTVRFPFRIPFTAYFEDGHVIDSVT
CP3          HIQPSVLVLNVTWASFNFIILLASIAVAHESRQIRNTVRFPFRIPFTAYFEDGHVIDSVT

                                  610       620       630       640       650       660
francensis   DNISLGGLAFSLPKNYELADRDITEISMKVDNRSFALPVKIVSRSGALVRLQFETASMSE
ATTC10988    DNISLGGLAFSLPKNYELADREISEISMKVDNRSFALPTKIVSKSGSLVRLQFEAASMPE
CU1          DNISLGGLAFSLPKNYELADREISEISMKVDNRSFALPTKIVSKSGSLVRLQFEAASMPE
CU1rif2      DNISLGGLAFSLPKNYELADREISEISMKVDNRSFALPTKIVSKSGSLVRLQFEAASMPE
uvs51        DNISLGGLAFSLPKNYELADREISEISMKVDNRSFALPTKIVSKSGSLVRLQFEAASMPE
B23394       DNISLGGLAFSLPKNYELADRDISEISMKVDNRSFALPTKIVSKSGSLVRLQFEAASMPE
Z6           DNISLGGLAFSLPKNYELADRDISEISMKVDNRSFALPTKIVSKSGSLVRLQFEAASMPE
B4492        DNISLGGLAFSLPKNYELADRDISEISMKVDNRSFALPTKIVSKSGSLVRLQFEAASMPE
PROIMIA1     DNISLGGLAFSLPKNYELADREISEISIFF..............................
NCIMB11163   DNISLGGLAFSLPKNYELADREISEISMKVDNRSFALPTKIVSKSGSLVRLQFEAASMPE
CP1          DNISLGGLAFSLPKNYELADREISEISMKVDNRSVALPTKIVSKSGSLVRLQFEAASMPE
B1960        DNISLGGLAFSLPKNYELADREISEISMKVDNRSVALPTKIVSKSGSLVRLQFEAASMPE
ATCC31822    DNISLGGLAFSLPKNYELADREISEISMKVDNRSFALPTKIVSKSGSLVRLQFEAASMPE
CP4          DNISLGGLAFSLPKNYELADRDIPEISMKVDNRSFALPTKIVSKSGSLVRLQFEAASMPE
B12526       DNISLGGLAFSLPKNYELADRDIPEISMKVDNRSFALPTKIVSKSGSLVRLQFEAASMPE
CP3          DNISLGGLAFSLPKNYELADRDIPEISMKVDNRSFALPTKIVSKSGSLVRLQFEAASMPE

                                  670       680       690       700       710       720
francensis   QKKLVAALMGQADAWQHYGPEEKPVSTIASMKHILKIIFGLLRYRPQKRINKKAAIKNEK
ATTC10988    QKKLVAALMGQADAWLHYGPEEKPVSTIASMKHILKIIFGLLRYRPQKRINKKAAIKNEK
CU1          QKKLVAALMGQADAWLHYGPEEKPVSTIASMKHILKIIFGLLRYRPQKRINKKAAIKNEK
CU1rif2      QKKLVAALMGQADAWLHYGPEEKPVSTIASMKHILKIIFGLLRYRPQKRINKKAAIKNEK
uvs51        QKKLVAALMGQADAWLHYGPEEKPVSTIASMKHILKIIFGLLRYRPQKRINKKAAIKNEK
B23394       QKKLVAALMGQADAWLHYGPEEKPVSTIASMKHILKIIFGLLRYRPQKRINKKAAIKNEK
Z6           QKKLVAALMGQADAWLHYGPEEKPVSTIASMKHILKIIFGLLRYRPQKRINKKAAIKNEK
B4492        QKKLVAALMGQADAWLHYGPEEKPVSTIASMKHILKIIFGLLRYRPQKRINKKAAIKNEK
PROIMIA1     ............................................................
NCIMB11163   QKKLVAALMGQADAWLHYGPEEKPVSTIASMKHILKIIFGLLRYRPQKRINKKAAIKNEK
CP1          QKKLVAALMGQADAWLHYGPEEKPVSTIASMKHILKIIFGLLRYRPQKRINKKAAIKNEK
B1960        QKKLVAALMGQADAWLHYGPEEKPVSTIASMKHILKIIFGLLRYRPQKRINKKAAIKNEK
ATCC31822    QKKLVAALMGQADAWLHYGPEEKPVSTIASMKHILKIIFGLLRYRPQKRINKKAAIKNEK
CP4          QKKLVAALMGQADAWLHYGPEEKPVSTIASMKHILKIIFGLLRYRPQKRINKKAAIKNEK
B12526       QKKLVAALMGQADAWLHYGPEEKPVSTIASMKHILKIIFGLLRYRPQKRINKKAAIKNEK
CP3          QKKLVAALMGQADAWLHYGPEEKPVSTIASMKHILKIIFGLLRYRPQKRINKKAAIKNEK
```

francensis	GAVQK
ATTC10988	GAVKK
CU1	GAVKK
CU1rif2	GAVKK
uvs51	GAVKK
B23394	GAVKK
Z6	GAVKK
B4492	GAVKK
PROIMIA1	.....
NCIMB11163	GAVKK
CP1	GAVKK
B1960	GAVKK
ATCC31822	GAVKK
CP4	GAVKK
B12526	GAVKK
CP3	GAVKK
